# Modeling the Spatial Dynamics of International Tuna Fleets

**DOI:** 10.1371/journal.pone.0159626

**Published:** 2016-08-18

**Authors:** Jenny Sun, Michael G. Hinton, D. G. Webster

**Affiliations:** 1 Gulf of Maine Research Institute, Portland, Maine, 04101, United States of America; 2 Inter-American Tropical Tuna Commission, La Jolla, California, 92037, United States of America; 3 Environmental Studies, Dartmouth College, Hanover, New Hampshire, 03755, United States of America; Sveriges lantbruksuniversitet, SWEDEN

## Abstract

We developed an iterative sequential random utility model to investigate the social and environmental determinants of the spatiotemporal decision process of tuna purse-seine fishery fishing effort in the eastern Pacific Ocean. Operations of the fishing gear mark checkpoints in a continuous complex decision-making process. Individual fisher behavior is modeled by identifying diversified choices over decision-space for an entire fishing trip, which allows inclusion of prior and current vessel locations and conditions among the explanatory variables. Among these factors are vessel capacity; departure and arrival port; duration of the fishing trip; daily and cumulative distance travelled, which provides a proxy for operation costs; expected revenue; oceanographic conditions; and tons of fish on board. The model uses a two-step decision process to capture the probability of a vessel choosing a specific fishing region for the first set and the probability of switching to (or staying in) a specific region to fish before returning to its landing port. The model provides a means to anticipate the success of marine resource management, and it can be used to evaluate fleet diversity in fisher behavior, the impact of climate variability, and the stability and resilience of complex coupled human and natural systems.

## Introduction

Space-based approaches provide key tools for marine resource management. Their use requires anticipating fishers’ response to management action, including their decisions about fishing location and effort to exert at a location. These are generally assumed due to the lack of suitable models anticipating fishermen’s future actions, and the success of resource management rests on the distance between assumption and realization. Methods to study stability and resilience in complex coupled-human-and-natural systems are sorely needed in the domain of decision-support tools for the management of marine fisheries resources. An abundance of methods applied to analyses of social and ecological systems has resulted in well studied system-specific dynamics and identified stability points, but the importance of variability and patchiness to ecosystem stability is not well understood, and inherent characteristics of patchiness may have a profound influence on system stability. Patchiness in human activities (e.g. fishing or pollution) results from maximizing benefit-cost ratios, often by exploitation of specific ecosystem patches (e.g. aggregations of spawning fish or discharging factory waste into a river). Mismatches in scales of patchiness of human activities have profound effects on the stability and governance of these complex coupled human-and-natural systems and lead to significant failures of resource management generally and of fisheries management specifically [1–5].

The problem of spatial complexity is particularly acute in large-scale high-seas fisheries, like those targeting tunas and tuna-like species, where an understanding of the spatiotemporal dynamics of both human and natural systems is lacking. To fill in gaps on the social side, we develop a method to model fleet dynamics in international fisheries and apply it to the purse-seine fishery for tropical tunas in the eastern Pacific Ocean (EPO). The fishery is managed by the Inter-American Tropical Tuna Commission (IATTC). Fishing occurs over an area of about 10^6^ km^2^ in the region from the coast of the Americas Mexico to Peru) to about 150°W [6]. This area is larger than the land mass of the United States or China and about the same size as Canada. Over 99 percent of the vessels in the tuna fishing fleet can carry more than 363 t of fish, and most have capacities between 1,000 to 1,500 t. Vessels this large can, and often do, make long fishing trips (median 45 days, maximum 172 days) and may travel from the coastal ports to the outer reaches of the EPO and back in a single trip. In spite of their mobility, these fleets face a tremendous challenge locating targeted species in such a vast area. In fact, costs of production would be prohibitively high in the purse-seine fishery if not for fishers’ ability to exploit knowledge of tuna behavior, their general distributions and habitat preferences, and their associations with dolphins and with floating objects as described below.

### Tuna Purse-Seine Fishery Fleet Dynamics in the eastern Pacific Ocean

**Fig 1:**
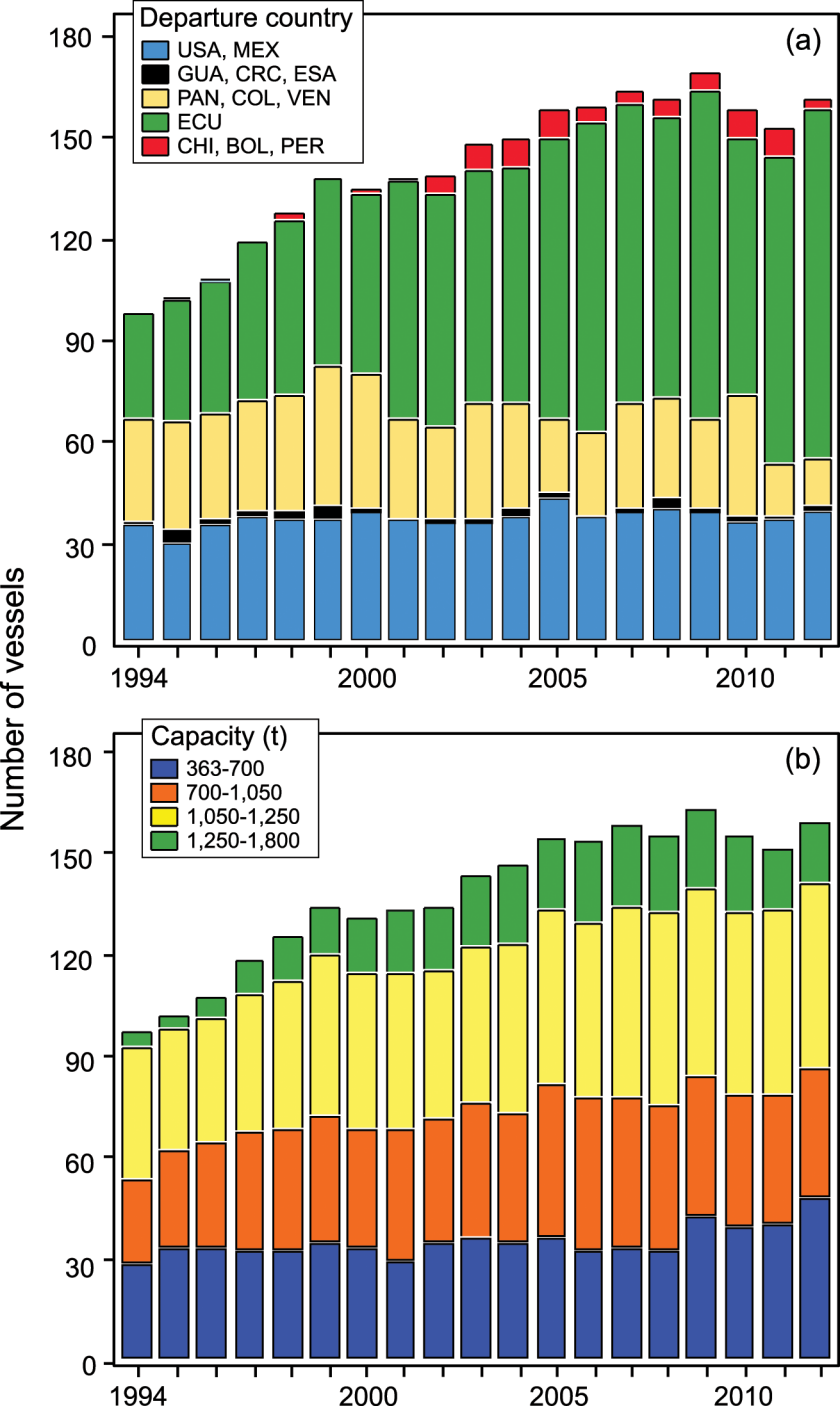
**Number of individual vessels by (a) country of departure and (b) carrying capacity (t) of fish by year.** (a) Country of departure is indicated by ISO-3166 International Standard Alpha-3 codes (http://www.iso.org/iso/country_codes). (b) Only vessels with carrying capacity of fish ≥ 363 t, which are required to carry observers, were included in the analysis. About 95% of the catch is taken by these vessels.

Fleets target different species and sizes of tunas using gear tuned to the particulars of individual vessel operations and fishing locations. Operations of these fishing fleets (Fig 1) are regulated by the 25 member states and three cooperating non-members of the IATTC. Combined, the regulatory control and the independent vessel operations result in a spatial patchwork of fishing effort and catch that can have profound effects on the resilience of the fishery as a whole [7–10]. Data documenting all activities related to fishing and vessel movements were compiled by onboard scientific observers for all trips by vessels included in this study. Dolphin mortalities occurring during fishing operations are strictly monitored and controlled. The IATTC serves as the secretariat for the Agreement on the International Dolphin Conservation Program, which ensures conservation of dolphin in the EPO by limiting annual dolphin mortalities to levels approaching zero. Vessels may target tunas by making sets around pods of dolphins with tunas swimming below (DEL, dolphin sets). In the early years of the fishery, this dolphin-associated fishing practice generated high levels of dolphin mortality and considerable political-economic conflict [11]. Today the dolphin conservation program places constraints on gear design and operation, requires training of skippers and crew, and restricts a vessel’s operations if it reaches or exceeds its assigned dolphin mortality limit (DML). If vessel operations result in dolphin mortalities greater than the limit, that vessel is prohibited from further operations targeting tunas with dolphins for the remainder of the calendar year. Vessels without a DML are allowed no incidental dolphin mortality and do not engage in dolphin-associated fishing.

Three métier, broadly distinguished by set type, exist in the purse-seine fishery. The length-distributions and species compositions of the tunas taken by each métier, and the principal regions of the EPO in which sets of each type are made, are different. The size of tunas taken in a set is governed by fish length. The maximum swimming velocity of tunas is determined by length, so though there may be a mix of species or cohorts in a school, the size of individuals will be similar. The smallest tunas taken by the purse-seine fishery are in sets targeting tunas associated with floating objects (OBJ) and are a mix of bigeye (BET; mean 53 cm, 8.2 kg), skipjack (SKJ; 45 cm, 1.8 kg) and yellowfin (YFT; 55 cm, 3.3 kg). Slightly larger SKJ (50 cm, 2.7 kg) and YFT (80 cm, 10.7 kg) are taken from free-swimming schools of tuna in non-associated (NOA) sets. Only the largest YFT (110 cm, 28 kg) are capable of maintaining sustained swimming speeds sufficient to keep up with and be taken in sets associated with pods of dolphin (DEL).

Prior to 1993, OBJ sets generally were made on flotsam or naturally-occurring objects, such as logs. Since 1993 OBJ sets have been made using man-made fish-aggregating devices (FADs) that have been placed by purse-seine vessels specifically to attract tunas for subsequent capture [12]. Large numbers of juvenile BET swim below schools of SKJ associated with FADs, and they are caught along with the SKJ, which are the target of these sets. Prior to the use of FADs, BET tunas of any size were rarely taken in purse-seine fisheries, and their capture in FAD sets was unexpected. The use of FADs was intended to reduce vessel search time and distance traveled in pursuit of tuna once dolphin fishing was limited by the dolphin protection agreement. By attracting schools of SKJ to the surface, this gear innovation allowed fishers to harvest SKJ that were previously unavailable to the fishery. This new métier was highly successful and by the early 1990s a fishery directed at exploiting the association of SKJ with floating objects had developed; by 1994 it was well established. OBJ set landings are now almost double DEL set landings. Technological improvements continue to make finding fish much easier. For instance, FADs now carry GPS tracking systems, echo sounders and sonar, and other electronics that can identify species composition and biomass of schools and fish size, which is transmitted to vessels using encrypted communications when a FAD is queried using the correct access codes.

Oceanographic features, fish habitat preferences, fisher behavior, and the proliferation of FADs have led to a spatial redistribution in operations of the purse-seine fleet. Regions of operation of vessels in each of the three métier overlap to a degree. Vessels that have DMLs tend to focus most of their effort on dolphin-associated schools, which tend to be found further north than schools associated with floating objects. There are areas where all three types of schools can be found, and fishers targeting adult YFT in association with dolphins will also set on OBJ and NOA schools when possible. Fishers without a DML do not have the option to shift to dolphin fishing, but they can switch targeting between object- and unassociated-schools. While fishers’ choice of search area will depend heavily on their preferred métier, the choice of making a set of a specific type depends on the size and composition of schools within the search area as well as regulatory restrictions on dolphin mortality (http://www.iattc.org/IDCPENG.htm) or restrictions for conservation of tunas (http://www.iattc.org/PDFFiles2/Resolutions/C-13-01-Tuna-conservation-in-the-EPO-2014-2016.pdf)

More recently, economic pressures have driven fishers to search for new ways to increase efficiency and reduce operating costs. The EPO is a very large area and some vessels shift between regions to maintain high catch levels, so distance traveled—and the related fuel costs—are important determinants of spatiotemporal decision-making in this fishery. Increasing costs associated with rising fuel prices were magnified by the economic downturn in 2008, and in spite of recent lower prices for fuel, fishers are still innovating to reduce fuel costs. In the short run, they can purchase larger vessels, which provide for economies of scale (reducing the price-per-ton to search for fish), and ships designed for more fuel efficient operations. Both increase the effectiveness of fishing fleets, allowing them to exploit larger areas or travel to distant areas more often. At the same time, fishers who cannot afford to purchase new vessels may make shorter but more frequent trips, increasing the intensity of effort in the coastal region.

We investigate the past distributions of fishing effort and provide a means to anticipate future changes in the distribution of fishing effort by purse-seine vessels in the EPO using a sequential random utility model. The process is sequential because a fisher’s choice to move from one search area to another is dependent on their current location. In open-ended interviews, tuna vessel captains and owners all stressed that location decisions during a trip in the EPO are hierarchical. Fishers make three first-tier decisions when they decide on the direction, duration, and métier, before departing on a trip. They then select specific fishing grounds based on real-time data on oceanographic conditions, current trip history, and information from other fishers. The first location decision is substantively different from all others, because fishers made the decision in port, rather than at sea, where current conditions for an area can be directly observed and used to guide future fishing decisions. We found that more than anything else, the location of the first set was a good indication of all three first-tier decisions.

This article is structured as follows. In the first section, we review the fleet dynamics literature and explain why methods used to date are not appropriate for large-scale fisheries. Next, we describe the data and methods that we used in our model, provide details on the specifications, and then present the results. We move from measures of fit and a close look at the first set models, and then examine the switching region choice model results. Finally, we relate these fishery-specific observations to the larger problems of modeling fleet dynamics and a broader understanding of patchiness in coupled human and natural systems.

### Literature Review of Individual Location Choice Model

Discrete choice models, such as RUMs, have been widely applied, including several applications to fisheries. McFadden [13,14] developed the first discrete choice random utility models in studies of urban travel and of investment in transportation infrastructure. Bockstael and Opaluch [15] were the first to apply these methods to fisheries. Their empirical work on New England fisheries focused on individual fisher choice to change fisheries, by switching species targeted and/or gear used. A number of similar papers followed [16–18]. Eales and Wilen [19] first applied RUMs to the spatial distribution of fishing effort. RUMs also have been used to study trawl and gillnet fisheries [20–23].

Most of the spatial and métier studies have focused on fisheries where trips visit one location in the fishing grounds and are of short (1–5 days) duration. Thus, the choice of location has been temporally independent. Schnier and Felthoven [24] warn that, even in these models, spatial autocorrelation in unobserved variables, such as habitat attributes, may violate the underlying assumption of independence across location choice options. They advocate the use of specific alternative constants to control for this missing variable bias. Smith [25] points out that it can be difficult to determine whether or not variations observed in discrete choice models are due to individual differences (heterogeneity) or to differences in individual experience (state dependence). He develops several models to distinguish between these often confounded variables and applies them to data from the sea urchin fishery off California. Like Schnier and Felthoven [24], Smith finds that state dependence is important and that autocorrelation between choices of location should not be ignored. If fisher experience was not included in his model, choices might otherwise appear independent. Swait et al. [26] reach similar conclusions in their study of recreational fishing around Perth, Australia.

In the purse-seine fishery studied here, an individual trip lasts from a few weeks to several months or longer. Over 20,000 sets are made annually by about 150 purse-seine vessels. This all complicates capturing individual fisher’s behavior. Fishers serially choose from multiple locations within a single trip, and it is unlikely that fishers can optimize over a trip lasting weeks or months. Decisions leading to location choice are strongly dependent on experience at previously visited and current locations. Curtis and Hicks [27] analyzed the economic impact of time-area closures intended to protect sea turtles encountered by the Hawaiian longline fishery for tuna and swordfish. Fishing trips were of about 2–4 weeks in this fishery. They included the current location and the previous experiences during the current trip in a RUM of location choice; however, their modeling approach assigned equal probability to all choices of location.

Hicks and Schnier [28] analyzed fisher response in the EPO to the “dolphin-safe” labeling campaign of the early 1990s. In their dynamic random utility model, fishermen chose their cruise path based on expected revenue. Their model was not appropriate for this study, because it is impossible for a fisher to know the optimal location and timing sequence of locations before beginning a trip. In addition, their data were limited to only those from U.S. vessels and from the period prior to 1993, which is a year before the development of the floating object fishery and prior to other fleets adjusting to the introduction of DML restrictions. Davies et al. [10] developed a binary choice model, but it was limited by using the aggregated fishing effort observed in a location without considering the constraint of the track of individual vessels. Their model was run on a monthly time scale. This was useful for tracking the seasonal movements they described, but it does not capture the daily determinants of the location of fishing effort presented in our analysis.

Random utility modeling requires the independence of alternative choices; however, this assumption is violated by serial correlation of decisions on multi-week trips. In the sequential model we addressed this violation by explicitly specifying prior and current vessel locations and conditions among the explanatory variables.

## Data and Methods

### Model Specification

We developed the full sequential random utility model using two sub-models. A fishing trip was divided into two parts based on the nature of a skipper’s decision process and inputs prior to putting to sea, in comparison to decision processes and inputs when at sea. The First set Model is applied for decisions made prior to vessel departure from port on a fishing trip. Once at sea, the dynamics of vessel operation and fishing changes the nature of information on which the skippers make decisions to change or maintain vessel activities and locations. The switching region choice model is applied for daily decisions on vessel operations after a vessel has left port. Although the definition of variables differs for each stage, the location-choice set used in each model is the same. First, consider a panel data set consisting of N vessel trips, where *i* is any individual trip that chooses region *r* among R alternative regions in time period t. Let *U*_*it*,*r*_ be the contemporaneous utility of choosing to set on a school of fish in location *r*, denoted as the *r*th element in the Rx1 vector *U*_*it*_, which represents the contemporaneous utility of all alternatives expected by the fisherman. However, we, the researchers, only observe a consistent unbiased estimate of vector *U*_*it*_, and we denote it as V_it_, through a set of measurable explanatory variables. The difference between *U*_*it*_ and *V*_*it*_ is captured by the random error component *ε*_*it*_ where *U*_*it*_ = *V*_*it*_ + *ε*_*it*_ and *ε*_*it*_ is independently and identically distributed with an extreme value distribution.

In general, the contemporaneous utility *V*_*it*_ across R alternatives is defined as follows,

$$\begin{array}{l} V_{it(Rx1)}=f(X_{it1\;(Rx1)},X_{it2(Rx1)},\ldots ,X_{itK(Rx1)},Z_{it1(RxR)},Z_{it2(RxR)},\ldots ,Z_{itJ(RxR)};\\ \;\beta_{1(1x1)},\beta_{2(1x1)}\ldots ,\beta_{k(1x1)},\theta_{1(Rx1)},\theta_{2(Rx1)},\ldots ,\theta_{j(Rx1)}) \end{array}$$ (1)

where there are two types of explanatory variables defined to explain the choice utility. Let *X*_*itk(Rx1)*_ be the kth feature in each alternative region for the ith trip at time t, where k = 1, 2, …, K, and *β*_*k*_ is the kth corresponding feature-specific parameter. The explanatory variables are assumed to be different values for the features of each alternative region. The impact of the X_*itk*_ variable on the utility of choosing the r^th^ region is derived from the differences in values across the alternative regions.

Environmental covariates, e.g. chlorophyll content and dissolved oxygen concentration, may be correlated. To avoid this potential problem, we transformed environmental data prior to including it in models (Table 1) as follows. Quantiles of the total number of 1° latitude by 1° longitude areas (1x1 areas) were computed for DEL sets, which are widely distributed across the region of the fishery, thus providing representative observations of environmental conditions that are associated with fishing trips in the data set. Those with values higher than the third quantile (or lower than the first quantile) of the distribution of each environmental variable in the selected 1x1 areas were identified. This is a way to evaluate the suitability of the environmental condition by region for DEL sets as a reference set to capture fishermen experience in response to the combination of the environmental condition variables without losing the dynamics of the environmental condition.

**Table 1:** Variable Definition and Description.

**Acronyms used in table: Arr** arrival; **BET** bigeye tuna; **Chloro** chlorophyll A concentration; **CPUE** catch-per-unit-effort (t/day); **DEL** dolphin; **Dep** departure; **DF** day fishing; **DML** dolphin mortality limit; **MEI** Multivariate El Niño-Southern Oscillation Index; **MLD** mixed layer depth; **NOA** unassociated; **O2** dissolved oxygen; **OBJ** floating object; **RPUE** revenue-per-unit-effort ($US/day); **SKJ** skipjack tuna; **SSH** sea surface height; **SST** sea surface temperature (°C); **YFT** yellowfin tuna;		
**Variable**	**Description and Formula Definition**	
**Dependent Variable by DML and Vessel Size Groupings:**		
• Decision	= 1 if selected, else = 0:: For each individual trip, a set of choices to select one fishing region from among the 12 regions.	
• DML	= 1 with dolphin mortality limit, else = 0:: Indicates if a vessel has a DML for a trip. DMLs are assigned in January and July of each year and are valid for a period of six months. However, if a vessel starts a trip with a DML, it will have a DML for that trip.	
• Vessel_Size	= 1 if in range, else = 0:: fish carrying capacity in t: small = 363–700 t; medium = 700–1,050; large = 1,050–1,250; extra-large = 1,250–1,800. (http://www.iattc.org/PDFFiles2/FisheryStatusReports/FisheryStatusReport13-2.pdf).	
**Independent Variables:** The natural logarithm of each variable is specified and prefixed with L in the table of estimation results.		
**Group 1. Trip-specific region characteristics:**		
• X1_CPUE_1	= lag1(log((YFT + BET + SKJ)/(DF_ALL+0.0001)+0.0001)). The regional average expectations of CPUE of YFT, SKJ and BET in the previous month. These serve as the average expectations of the regional CPUE in the current month.	
• X2_RPUE_1	= lag1(log((YFT_all*YFTP+BET_ALL*(SKJP+100)+SKJ_ALL*SKJP)/(DF_ALL+0.00001))). The regional average expectations of RPUE of YFT, SKJ and BET in the previous month. These serve as the average expectations of the regional RPUE in the current month. YFTP and SKJP are the monthly ex-vessel price by species. It was assumed that the ex-vessel price for juvenile BET tuna was $100 greater than SKJP.	
• X13_Dist_Exp	The expected geodistance (km) from home port (or current region) to each of the 12 regions for the first (or next) set.	
• X14_Dist_Arr	The expected geodistance (km) from an arriving port after travelling to each the 12 regions.	
**Group 2. Environmental Condition Variables:** Based on conditions observed on DEL sets (see text). Suffixes _L and _H are used to indicate the environmental conditions at a set relative to the daily total number of 1x1 squares in each region: _L = Environmental Condition (Observed) < first quartile (Environmental Condition); _H = Environmental Condition (Observed) > third quartile (Environmental Condition).		
• X3_SST_DEL_L	= total number of 1x1 squares with SST < 26.8	
• X4_SST_DEL_H	= total number of 1x1 squares with SST > = 28.7	
• X5_SSH_DEL_L	= total number of 1x1 squares with SSH < 0.6	
• X6_SSH_DEL_H	= total number of 1x1 squares with SSH > = 0.7	
• X7_MLD_DEL_L	= total number of 1x1 squares with MLD < 18.6 (lower than the first quartile of the distribution of MLD from DEL sets).	
• X8_MLD_DEL_H	= total number of 1x1 squares with MLD > = 38.8 (greater than the third quartile of the distribution of MLD from DEL sets).	
• X11_O2_DEL_L	= total number of 1x1 squares with O2 < 0.4	
• X12_O2_DEL_H	= total number of 1x1 squares with O2 > = 1.0	
**Group (3). Trip Performance Record Variables:** Each of the following variables is specified as a set of orthogonal dummy variables and indicated with subscript _Z1, _Z2, …, _Z11. A variable_Z12 is omitted to avoid the multicollinearity issues. These are specified to capture the impacts on the probability of next travelling to each of the 12 regions for the next set.		
• Z1_Dist_Dep	Geodistance (km) traveled since departure based on the track recorded in a daily basis.
• Z2_DF_Dep	Sum of hours (days) since departure
• Z3_DF_Travel_Dep	Sum of hours (days) recorded as travelling and drifting since departure.
• Z4_MEI	Monthly value of MEI. These are specified to capture MEI impact on the probability of travelling to each region for the next set in comparison to traveling to the base region, Region 12.
• Z5_DF_Search_Dep	Sum of hours (days) spent on searching since departure.
• Z6_SKJ_Dep	Total catch (t) of SKJ tuna since departure.
• Z7_YFT_Dep	Total catch (t) of bigeye tuna since departure.
• Z8_BET_Dep	Total catch (t) of bigeye tuna since departure.
• Z9_DF_Travel_Last	Sum of hours (days) recorded as travelling and drifting in the last fished region
• Z10_DF_Search_Last	Sum of hours (days) recorded as search time in the last fished region
• Z11_DEL_Last	Number of DEL sets fished in the last fished region
• Z12_OBJ_Last	Number of OBJ sets fished in the last fished region
• Z13_NOA_Last	Number of NOA sets fished in the last fished region
• Z14_SKJ_ Last	Total catch (t) of SKJ tuna since departure.
• Z15_YFT_ Last	Total catch (t) of YFT tuna since departure.
• Z16_BET_ Last	Total catch (t) of bigeye tuna since departure.

The other type of explanatory variable is uniform across regions during any given decision but varies across vessels (all trip preference record variables) or only varies temporally (general oceanographic conditions like the Multivariate El Niño-Southern Oscillation Index). To capture the effect of these variables, let *Z*_*itj(RxR)*_ represent the *jth* characteristics of individual trip *i for j = 1*,*2*, *…*, *J*. This is the jth R×R diagonal matrix in which the entries outside the diagonal are all zero and the diagonal entries themselves are the same as the jth group of spatially homogenous variable *Z*_*itj*_. Therefore *Z*_*itj*_ ⊗ I_r_ defined as *Z*_*itj*_ is the corresponding rth diagonal element in the R×R matrix, where I_R_ is the R×R identity matrix with ones on the main diagonal and zeros elsewhere. Columns in the R×R diagonal matrix *Z*_*itj(RxR)*_ are orthogonal, i.e. all elements are independent without possibility of collinearity.

Even though the variables *Z*_*itj*_ are constant across regions, for each of the rth region modes the impact of a unit change in *Z*_*itj*_ to the utility across alternative regions can be assumed to be different, because it captures a different state of the trip or the general oceanographic conditions. For example, distance travelled since departure for ith trip at point of time t could be alternative-specific, since distance travelled may not have the same impact on utility of choosing Region m verse choosing Region n. The coefficients ***θ***_j(Rx1)_ *=* [***θ***_j1,_ ***θ***_j2, …,_ ***θ***_jR_] represent an Rx1 vector of regression coefficients that capture the effects of the Z_itj_ variables on the utility of choosing the corresponding *r*th alternative region. By setting the last element of coefficients to null (that is, ***θ***_jR_ = 0), the coefficients ***θ***_jr_ represent the effects of the *Z*_*itj*_ variables on the probability of choosing the rth alternative over the last alternative to avoid collinearity across various Z_itj_ variables.

By combining the features of each alternative region and the characteristics of the individual vessel, the choice probabilities of choosing the *r*th alternative region, Π_*it*,*r*_, are specified in the mixed logit model that denotes the choice probabilities as follows,

$$\Pi_{it,r}=\frac{exp(V_{it,r})}{\sum_{r=1}^{R}exp(V_{it,r})}$$ (2)

where $\sum_{r=1}^{R}\Pi_{it,r}=1,$ ensuring that the sum of probabilities of all possible outcomes of any choice is always one and where the ith trip is assumed to select alternative region m whenever

$$V_{it}\;in\;region\;m>V_{it}\;in\;region\;n,\;i\in R,\;\forall k\in R$$ (3)

Each individual is allowed to choose one and only one of the possible alternatives. The variable *Decision* takes value 1 when a specific alternative is chosen; otherwise it takes value 0. This mixed logit model is similar to the conditional logit model [13,14] but allows for parameters that are randomly distributed and therefore captures variations in the preferences of individual decision makers.

As noted above, there is a general condition of independence of alternatives when using RUMs. Our model deals with this problem in two ways. First, we model the choice of first set separately, as this is most indicative of the fisher’s broader trip strategy ($\Pi_{it,r}^{First}$). We then embed trip experiences in a sequentially applied model of switching choice to estimate parameters for subsequent decisions ($\Pi_{it,r}^{Switch}$). Thus, the Rx1 vector of utility of fishing at the rth region location for the first set can be specified as

$$V_{it}^{First}(X_{it}^{First},Z_{it}^{First};\beta^{First},\Theta^{First})=\sum_{k=1}^{K}X_{itk}^{First}∙\beta_{k}^{First}+\sum_{j=1}^{J}Z_{itj}^{First}⊗I_{r}∙\theta_{j}^{First}$$ (4)

where the $X_{it(RxK)}^{First}=[X_{it1},X_{it2},\ldots ,X_{itK}]$ is an RxK matrix that contains all region-specific variables, including trip-specific region characteristics and oceanographic region characteristics during their first set, and *β*^*First*^_(Kx1)_ = [β_1_, β_2_…, β_K_]’ and **Θ**^*First*^_(JRx1)_ = [*θ*_1_,*θ*_2_,…*θ*_*j*_]′ is the RJx1 vector of parameter estimates corresponding to the group of spatially homogenous variable $Z_{it}^{First}$ during their first set. From this the probability of choosing the rth region for the first set can be specified and estimated using a mixed logit regression:

$$\Pi_{it,r}^{First}=\frac{exp(V_{it,r}^{First}(X_{it}^{First},Z_{it}^{First};\beta^{First},\Theta^{First}))}{\sum_{r=1}^{R}exp(V_{it,r}^{First}(X_{it}^{First},Z_{it}^{First};\beta^{First},\Theta^{First}))}$$ (5)

Similarly, the Rx1 vector of utility of switching region to fish in a different region can be specified as

$$\begin{array}{l} V_{it}^{Switch}(X_{it}^{Switch},Z_{it}^{Switch};\beta^{Switch},\Theta^{Switch})\\ \;=\sum_{k=1}^{K}X_{itk}^{Switch}∙\beta_{k}^{Switch}+\sum_{j=1}^{J}Z_{itj}^{Switch}⊗I_{r}∙\theta_{j}^{Switch}. \end{array}$$ (6)

The probability of choosing the rth region for the first set and subsequent sets in a different region can also be specified and estimated using a mixed logit regression. When predicting vessel choices, we expect them to select the region with the highest probability from the mixed logit model. Note that all regions are considered in the selection (switching) functions. It is possible that the region in which the vessel currently is located is preferred, in which case the vessel would be expected to remain in that region until a change in parameter values increases the probability of selecting an alternative region. Predictions are calculated sequentially starting with the first set model and thereafter iteratively applying the switching model until a vessel trip terminates due to exogenous restrictions on the home port and total distance (or days) that can be traveled by vessels of given size.

We estimated the two stages of the sequential model using the multinomial discrete choice procedure in Statistical Analysis Software Version 9.3 (http://www.sas.com/en_sg/software/analytics/stat.html). Because observations of the dependent variable, *Decision*, are broken down by whether or not a vessel has a dolphin mortality limit DML and by four vessel size classes (Vessel_Size), each stage in the model produces eight sets of results. Independent variables are the same for all DML and vessel size groups, though estimated parameters differ.

Measures of goodness of fit were obtained from the Aldrich-Nelson Index and the McFadden LRI. Measures of 0.6 or greater from the Aldrich-Nelson can be interpreted as an excellent fit, and the McFadden LRI on the range of 0.2–0.4 can be considered an indicator of excellent fit. We evaluate the predictive capacity of the model by calculating the percentage of location choices that are perfectly predicted.

### Data and Variable Definition

Model estimation was completed using the IATTC’s vessel log book and observer data, which is one of the most detailed fisheries datasets available. We incorporate satellite-based oceanographic data and outputs from general circulation models. The fishery dataset included over 1.4 million event-based, temporally sequenced records with 238 variables. In total, the dataset contains detailed information on 7,572 trips with 335,987 sets by three set types that travelled a cumulative 77 million km and spent a total of 194,678 days in fishing, searching and travelling to catch a total of 6.4 million tons of tunas. The set-by-set data were collected by scientific observers. The IATTC requires 100% observer coverage for all trips of large (capacity > 363 t) purse-seine vessels fishing on tuna in the EPO. Data were aggregated to 1° latitude by 1° longitude by day by trip to ensure confidentiality. During this period the annual average area (km^2^) in which fishing effort occurred was only 68 percent (range 63 to 73 percent) of the 10^6^ km^2^ EPO region over which the fishery operates.

**Fig 2:**
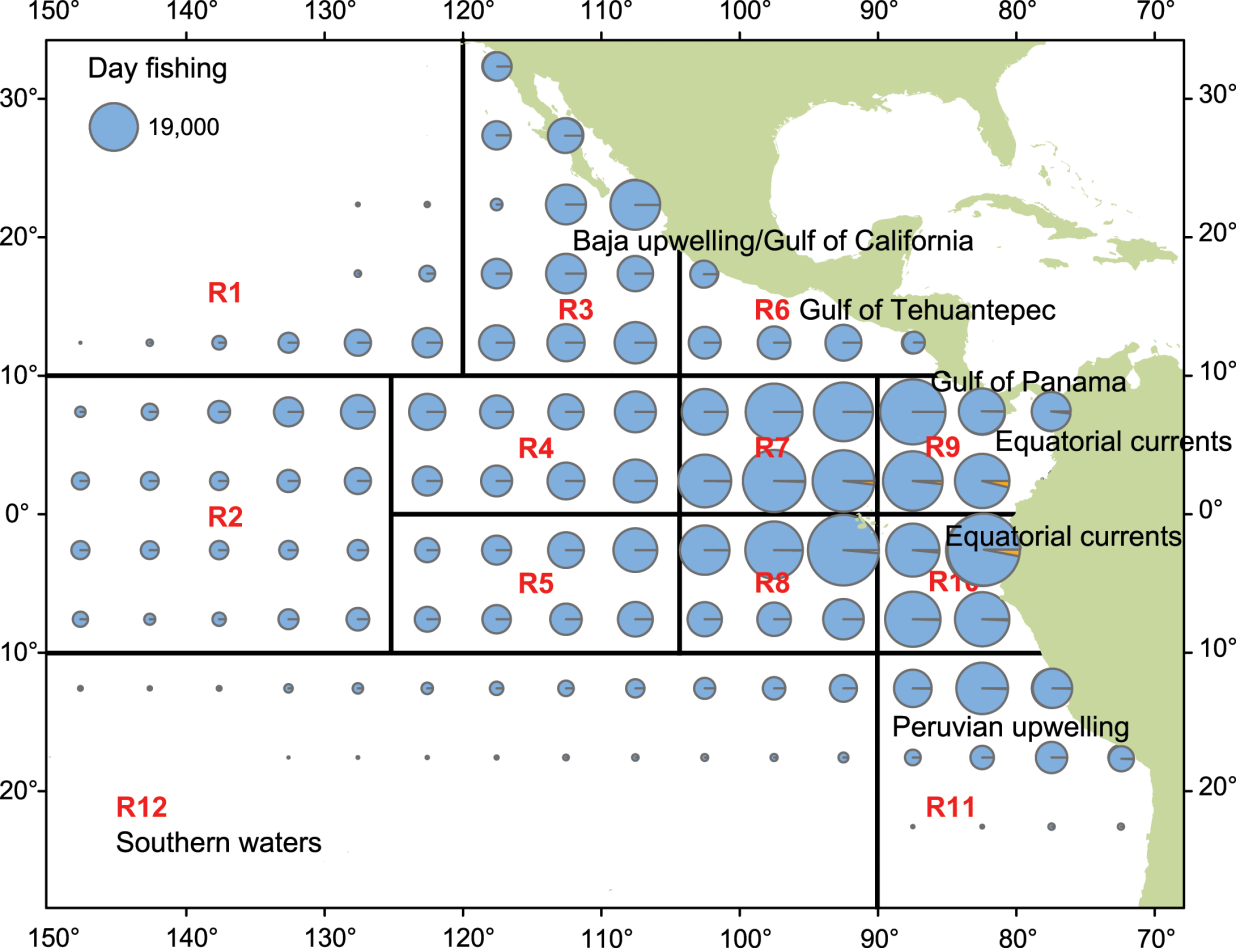
Twelve EPO regions used in the study and the number of days fishing by region, 1997–2012.

**Fig 3:**
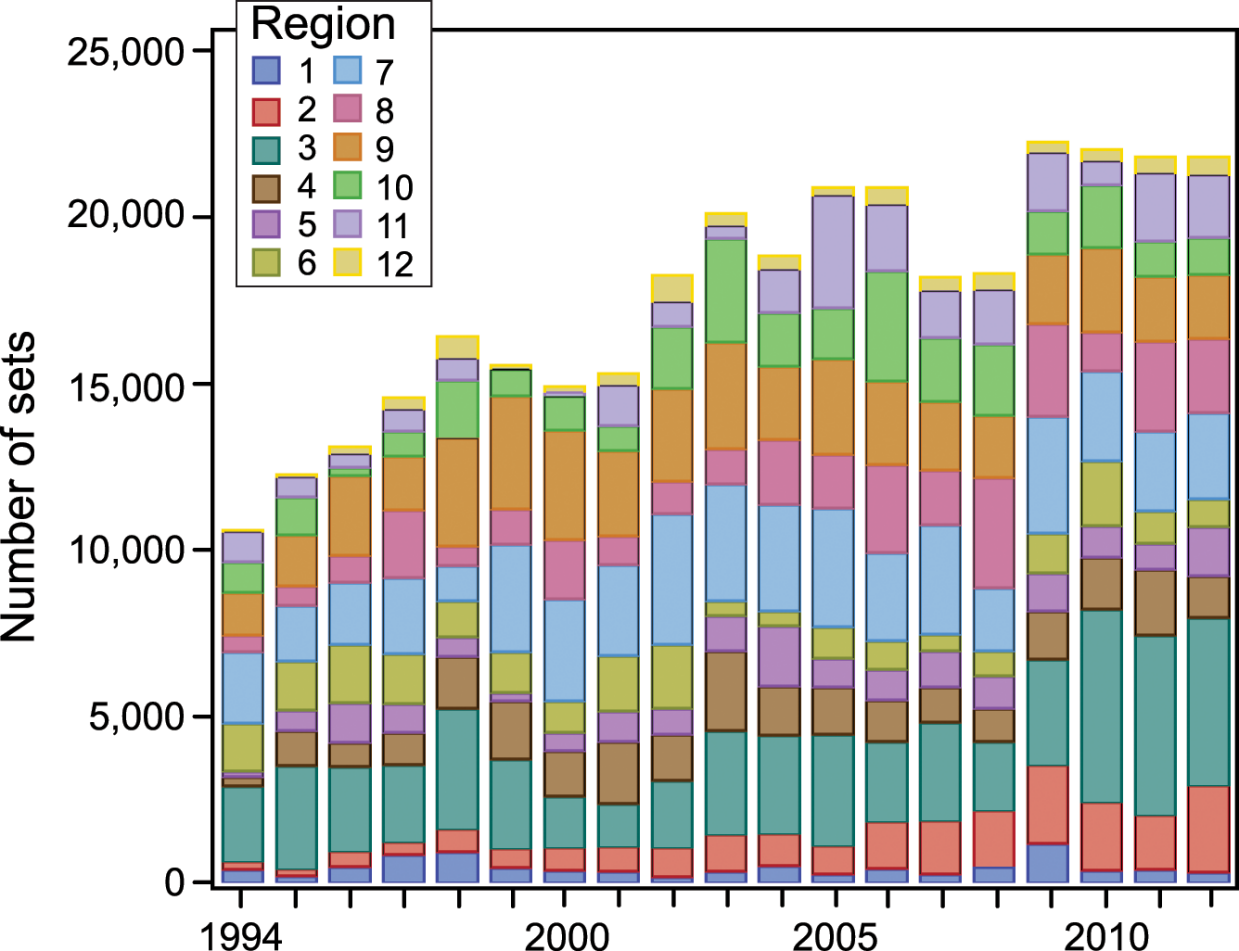
Number of purse-seine sets by EPO region, 1997–2012.

Given that the total area fished is vast, spatial location choice data were categorized by region to ensure sufficient data in each region to preserve model predictive capacity. Based on observed fishing patterns, we divided the EPO fishing area into twelve regions (Figs 2 and 3) that were intended to align with oceanography and with métier and fleet operations such that the number of observations of trips in each region during 1997–2012 was sufficient to provide a reasonable expectation of selection.

Our dependent variable is coded as the decision to set in one of the twelve regions shown in Fig 2, and is represented by the region code (see Table 1). This provides 16 models, eight for the first set decision and eight for the switching decision. Multiple models are necessary because vessels with a DML are essentially using a different métier than vessels without a DML, although some DML seiners that mostly fish on dolphin-associated sets will catch SKJ and BET tuna when they set on either OBJ or unassociated sets (NOA). Behavior is also expected to differ substantially by vessel size, as the maximum trip length, fuel costs, and other factors vary by the size of the fishing vessel. In general, smaller vessels are confined to shorter trips (duration) and so, even though they can certainly travel to the outskirts of the EPO in a single two-week trip, they would then have to return to port before heading out again, so their calculus is quite different from larger vessels that stay out for months at a time.

Because costs cannot be measured directly, we use distance as a proxy. This is reasonable because fuel is about 50% of the operating cost for vessels in the purse-seine fleet. We calculate two distance variables, one from the vessel’s current position to the mid-point of each region (Distant_Expected) and the second from the mid-point of each region to the vessel’s arrival port, where they will unload their catch (Distant-Arrive). Only Distant_Expected is included in the first set model, but both are included in the switching model because they capture two different costs. Both distance variables should be negatively correlated with the probability of location choice, unless there is some interaction with expected revenues. As a proxy for expected revenue we calculated a one month lagged catch-per-unit-effort (CPUE, tons/day fished), and weighted that by the species-specific price of tuna in that month to obtain the lagged revenue-per-unit-effort (RPUE) in each region. Monthly ex-vessel price data for cannery grade tuna were compiled for each species via personal communications from representatives of the canneries in Ecuador and in Thailand and used to define RPUE. Both CPUE and RPUE variables should be positively correlated with region choice unless there is an interaction with *Distance*. Thus our expectations for the sign of CPUE and RPUE will be somewhat unpredictable if there are high-revenue regions that are also high-distance regions and vice versa. This is sub-optimal, but we felt that a simpler representation of expected profits would be better in the first run of this already complex model.

Environmental condition variables are both general and region-specific. General oceanographic conditions include the Multivariate El Niño-Southern Oscillation Index (MEI) [29] and a dummy variable for the period from October to March in a given year. Both indicators are proxies for changes in weather and patterns of ocean properties that affect the distribution of effort because of increased safety concerns and technical constraints in stormy periods. MEI correlates with the direction and velocity of currents, and with the depth of the thermocline-oxygen barrier to tuna movement (see below). It is expected to correlate with changes in distributions of fishing vessels that are responding to spatial shifts in their operating environment and in the tuna resource. Oceanographic data compiled by region included chlorophyll content, sea surface height, mixed layer depth and sea surface temperature. Tunas have high oxygen requirements compared to other fishes, and the low oxygen concentration and low temperatures below the thermocline act in concert to create a boundary that limits the depth to which tuna dive [29–32]. For tractability, we chose to represent this thermocline-oxygen boundary by the dissolved oxygen concentration at a depth of 150 meters [33].

Data on the trip performance record provides basic information of the status of the vessel at a given set-choice. Here again, distance serves as a proxy for costs, although fishers must also keep track of distance traveled since departure (Distant_SinceDep) to ensure that they can return to port given limited supplies on board. Search time and travel time are also important decision factors, as there is a difference between a fisher who travels far in expectation of a high catch and a fisher who has spent considerable time searching between sets or before their first set. We include travel since departure (travel_sinceDep) and search since departure (search_sinceDep) in both models, and add travel since last set-region (travel_LastRegion) and search since last set-region (search_LastRegion) to the switching model. Similarly, fishers explained that their success on a given trip may influence their set choice. This is a complicated part of the decision process that we represented in this analysis by calculating the sum of the catch since departure and the sum of the catch in the last region fished by species. These variables are only included in the switching model and all variables are described in Table 1.

## Results

### Fit and Prediction

We fitted both the first set model and the switching region choice model using set-by-set data from 1997–2011, and we then simulated the probability of location choices in each region for trips in 2012 to test the out-of-sample prediction capacity by comparing predicted location choices to observations for the year 2012. We started the model in 1997 because there were constraints on the availability of satellite imagery (e.g. chlorophyll estimates) and because the fleet composition was relatively stable by this time following the influx of FAD-oriented vessels in the early 1990s. There were 200–225 vessels using each métier throughout the period. For random utility models, fit and predictive capacity are based on a range of goodness of fit measures. We looked first for consistency of fit, then at the actual level of fit, which was good to excellent for all models. The Estrella measure was 0.9525 for the first set model and 0.8199 for the switching region choice model. These measures indicate that the first set and the switching region choice models explain more than 80% of the variation in fisher choice of the location. The number of observations is relatively high for all sub-models (min = 408). Variation in goodness of fit seems to depend on the homogeneity of fishing strategies within a group rather than the number of observations. Specifically, for both DML and non-DML vessels, goodness of fit measures are generally higher for smaller vessels, which tend to have fewer operational options and therefore more homogenous fishing strategies. See Table A in S1 Appendix and Table B in S2 Appendix for a complete list of estimation result and goodness of fit indicators for the first set and switching region models, respectively.

The out-of-sample predictive capacity is also quite good for both first set and switch-region models. For first sets (Table 2) the average probability of showing a perfect prediction of region choice was 52% (32% to 100% across all departure countries, DML, and Vessel_size combinations). For the switching region choice model (Table 3) the fit was not as good as for the first set model, which was expected based on the greater potential variation. The average probability of obtaining a perfect prediction of switch in regions across all 92 region/DML/size combinations is 30%. These measures are confirmed visually in the plots of observed vs. predicted number of trips in each region by period, DML, and vessel size profiles for 2012 (Fig A in S3 Appendix and Fig B in S4 Appendix). In some regions, there are seemingly large differences between predicted and observed trips per region; however, the distribution across region is consistently predicted.

**Table 2:** Percentage of Perfectly Predicted Choices for First Set Regions.

Period	DML	Vessel Size	Observed (A)	Perfect Fit (B)	B/A (percent)
1997–2011	0	1_Small (363-700t)	1,927	911	47
		2_Median (700–1,050t)	985	383	39
		3_Large (1,050–1,250t)	408	160	39
		4_XLarge (1,250–1,800t)	590	268	45
	1	1_Small (363-700t)	539	372	69
		2_Median (700–1,050t)	1,555	928	60
		3_Large (1,050–1,250t)	2,240	1,380	62
		4_XLarge (1,250–1,800t)	640	321	50
	Total in 1997–2011		8,884	4,723	53
2012	0	1_Small (363-700t)	173	82	47
		2_Median (700–1,050t)	77	34	44
		3_Large (1,050–1,250t)	24	6	25
		4_XLarge (1,250–1,800t)	38	10	26
	1	1_Small (363-700t)	45	38	84
		2_Median (700–1,050t)	99	66	67
		3_Large (1,050–1,250t)	212	135	64
		4_XLarge (1,250–1,800t)	24	10	42
	Total in 2012		692	381	55

**Table 3:** Percentage of Perfectly Predicted Choices for Switching Regions.

Period	DML	Vessel Size	Observed (A)	Perfect Fit (B)	B/A (percent)
1997–2011	0	1_Small (363-700t)	4,755	1,447	30
		2_Median (700–1,050t)	3,733	973	26
		3_Large (1,050–1,250t)	1,574	364	23
		4_XLarge (1,250–1,800t)	2,266	586	26
	1	1_Small (363-700t)	855	332	39
		2_Median (700–1,050t)	5,332	1,409	26
		3_Large (1,050–1,250t)	8,616	2,198	26
		4_XLarge (1,250–1,800t)	2,385	612	26
	Total in 1997–2011		29,516	7,921	27
2012	0	1_Small (363-700t)	537	165	31
		2_Median (700–1,050t)	323	99	31
		3_Large (1,050–1,250t)	82	17	21
		4_XLarge (1,250–1,800t)	148	31	21
	1	1_Small (363-700t)	60	20	33
		2_Median (700–1,050t)	259	77	30
		3_Large (1,050–1,250t)	767	187	24
		4_XLarge (1,250–1,800t)	91	21	23
	Total in 2012		2,267	617	27

Within-sample simulation period = 1997–2011; Out-of-sample prediction period = 2012

**Fig 4:**
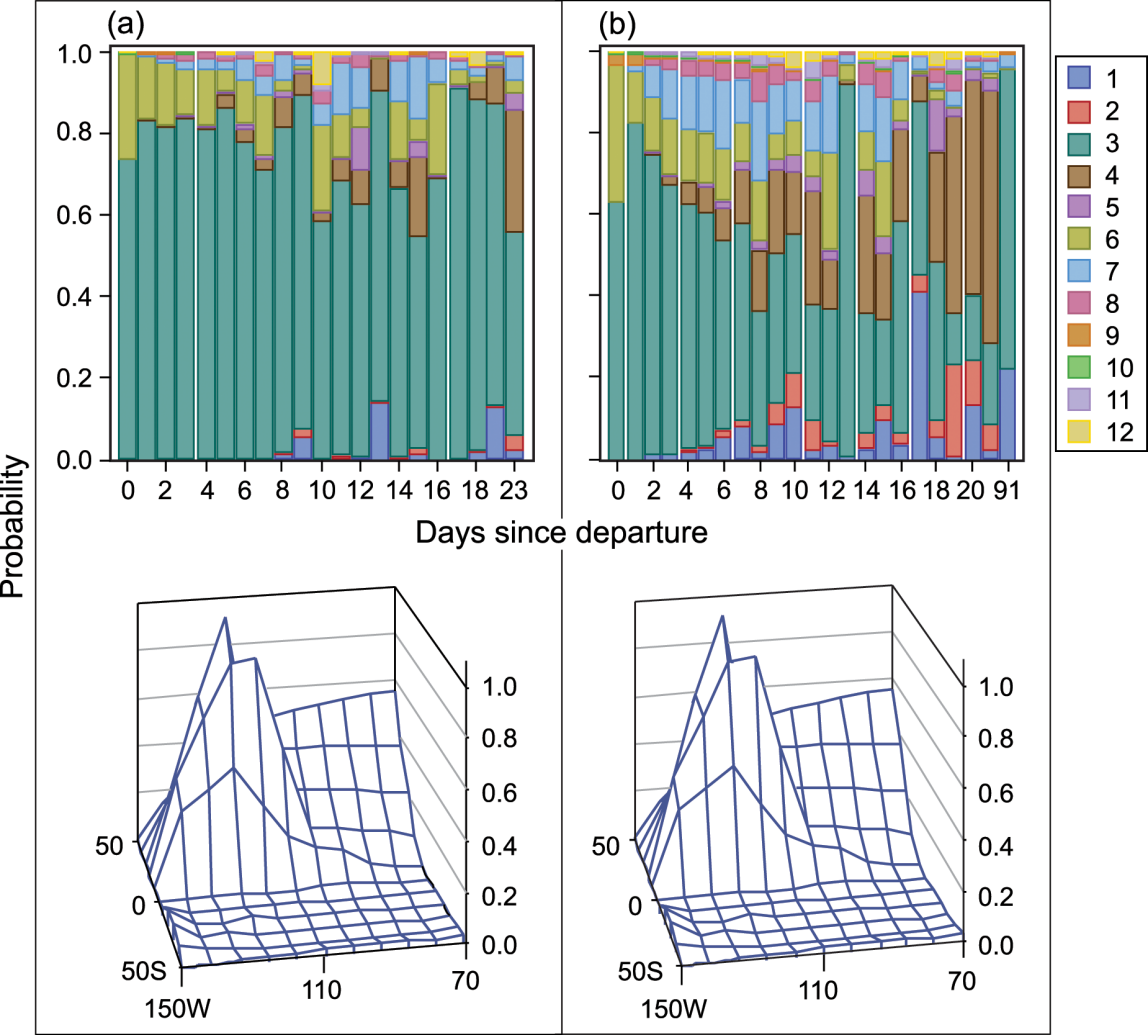
P(region of first set | days since departing port) [Upper panel] and its spatial distribution [Lower panel]. Vessels were categorized by carrying capacity, departure country, and vessel dolphin mortality limit: (a) 363–700 t, USA or MEX, Yes; and (b) 1,050–1,250 t, USA or MEX, Yes

**Fig 5:**
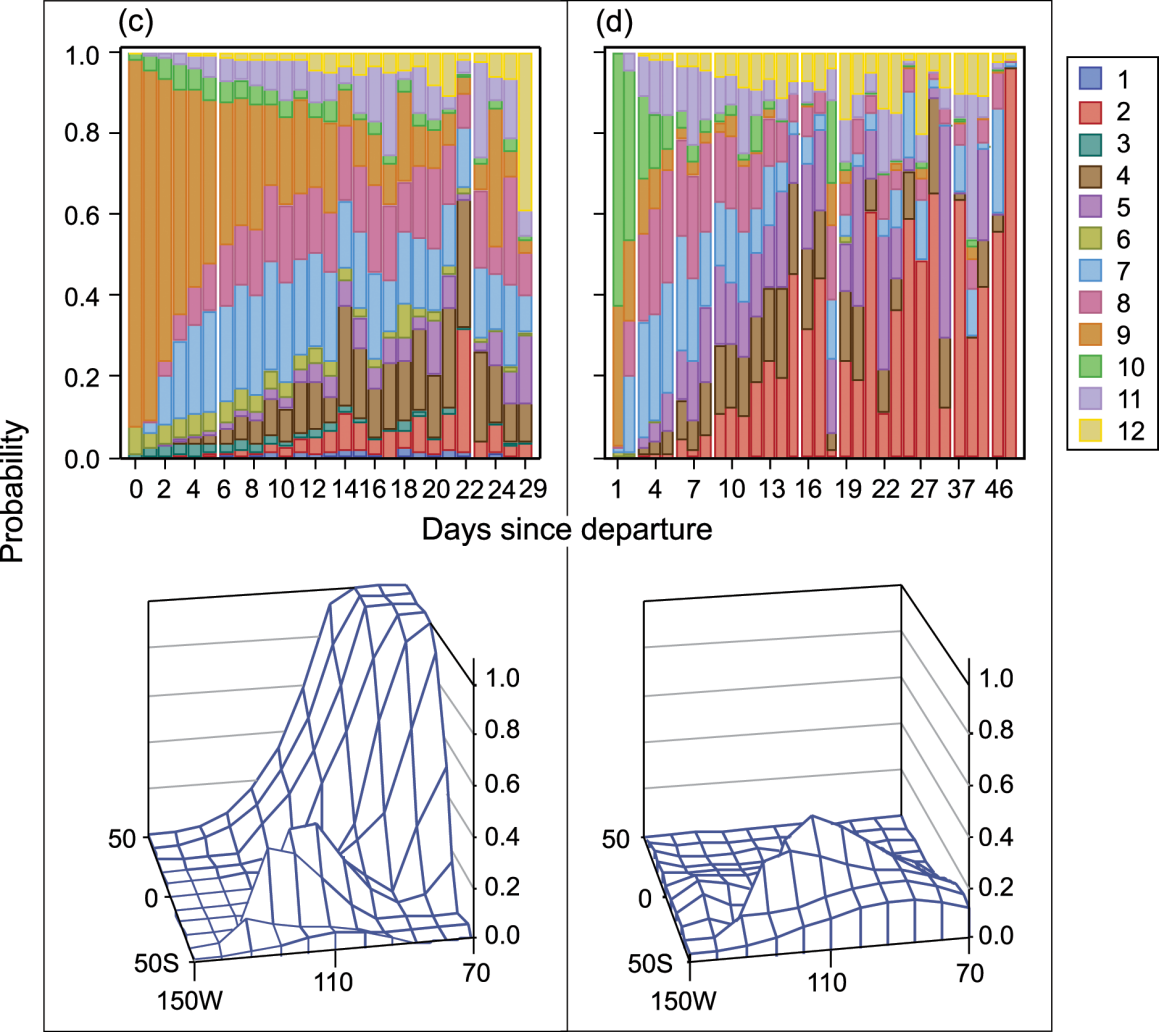
P(region of first set | days since departing port) [Upper panel] and its spatial distribution [Lower panel]. Vessels were categorized by carrying capacity, departure country, and vessel dolphin mortality limit: (c) 1,050–1,250 t, PAN, COL, or VEN, Yes; and (d) 1,250–1,800 t, ECU, No.

**Fig 6:**
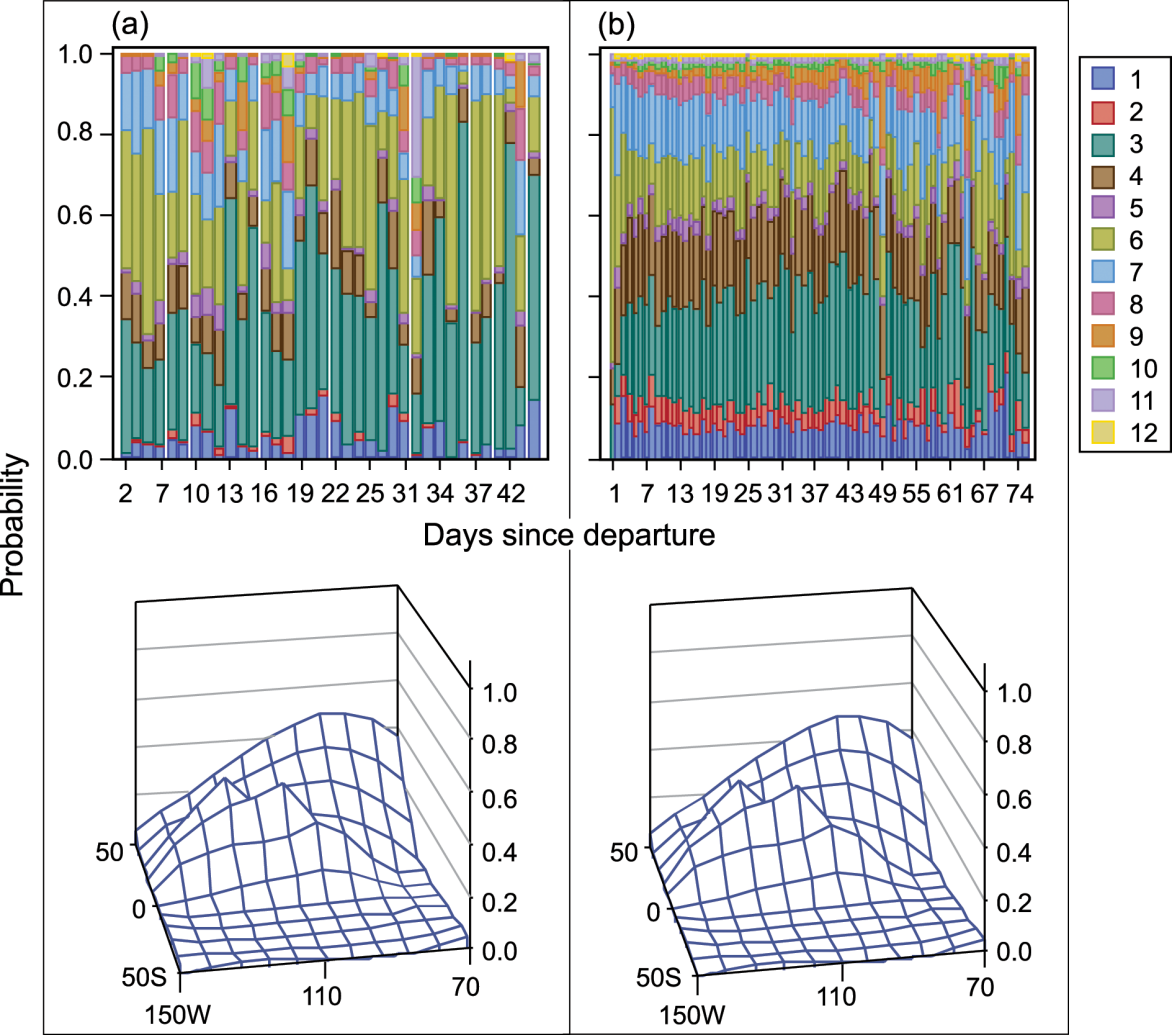
P(choice of subsequent region | days since departing port) [Upper panel] and its spatial distribution [Lower panel]. Vessels were categorized by carrying capacity and vessel dolphin mortality limit: (a) 363–700 t, Yes; and (b) 1,050–1,250 t, Yes.

**Fig 7:**
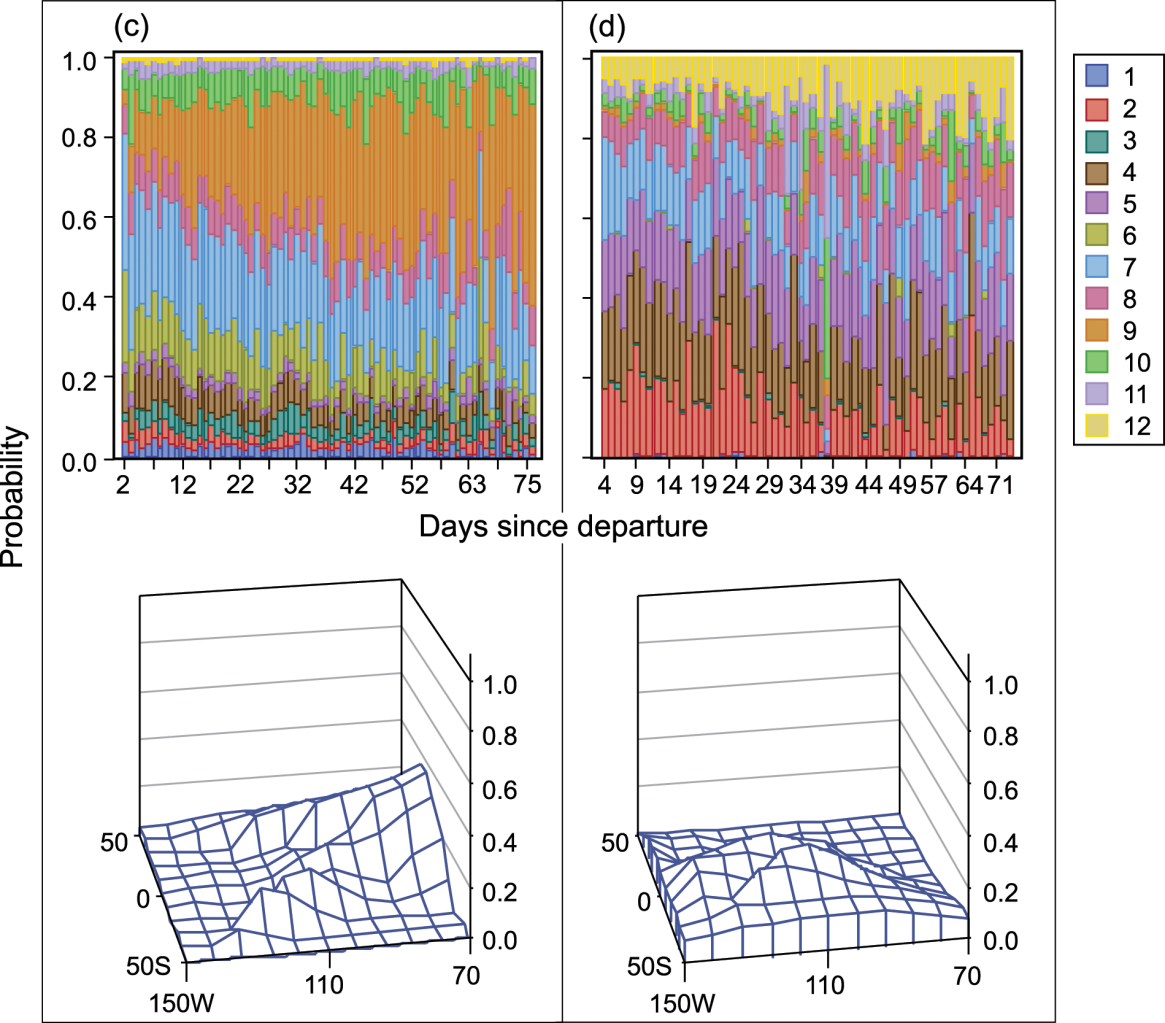
P(choice of subsequent region | days since departing port) [Upper panel] and its spatial distribution [Lower panel]. Vessels were categorized by carrying capacity and vessel dolphin mortality limit: (c) 1,050–1,250 t, Yes; and (d) 1,250–1,800 t, No.

The tendency of smaller vessels to fish nearer to the coast and for larger vessels to travel farther more frequently is clearly shown in the discrete response profile for each group of activities. Fig 4 and Fig 5 show the predicted probability of the region of the first set on a fishing trip, and Fig 6 and Fig 7 show the predicted probability of the region of the second and subsequent sets by days since departure, country of departure, possession of a DML, and vessel size. Vessels with a DML have increased options for set type and therefore have the opportunity to incorporate different fishing strategies than do vessels without a DML. As expected based on our interviews with fishermen, vessels without a DML tend to make their first set either in equatorial or southern regions, reflecting two first set strategies. All vessels with a DML use these two strategies, but larger vessels travel farther west before placing their first set more often than do smaller vessels. For vessels with a DML, most first sets occur in regions near the coast, but there is some variation between northern and equatorial search patterns, probably based on starting port location. Most fishers targeting tuna in association with dolphin operate from Mexican ports.

**Fig 8:**
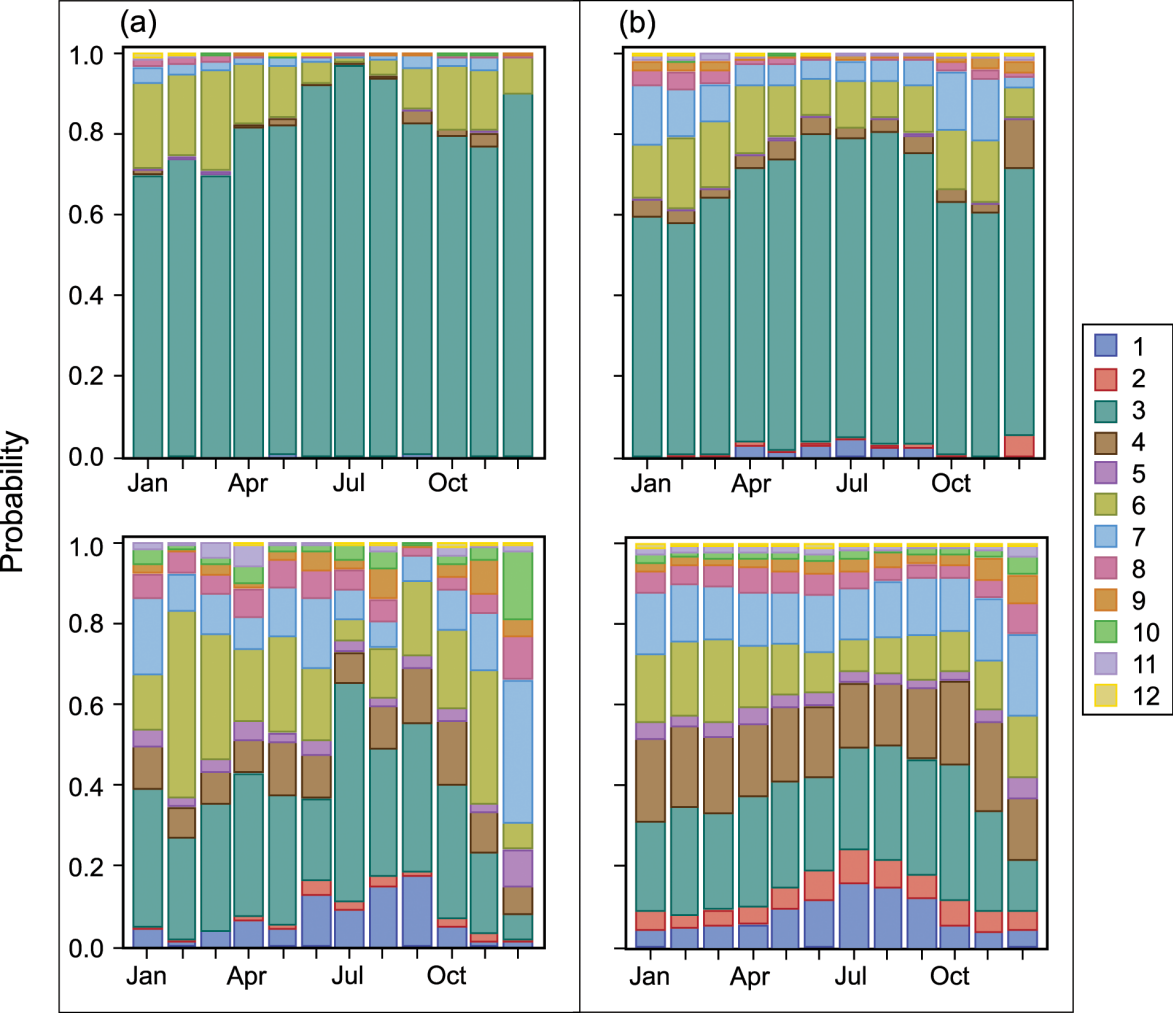
P(region of first set | month) [Upper panel] and P(choice of subsequent region | month). Upper panels: Vessels categorized by carrying capacity, departure country, and vessel dolphin mortality limit: (a) 363–700 t, USA or MEX, Yes; and (b) 1,050–1,250 t, USA or MEX, Yes. Lower panels: Vessels were categorized by carrying capacity and vessel dolphin mortality limit: (c) 1,050–1,250 t, Yes; and (d) 1,250–1,800 t, No.

**Fig 9:**
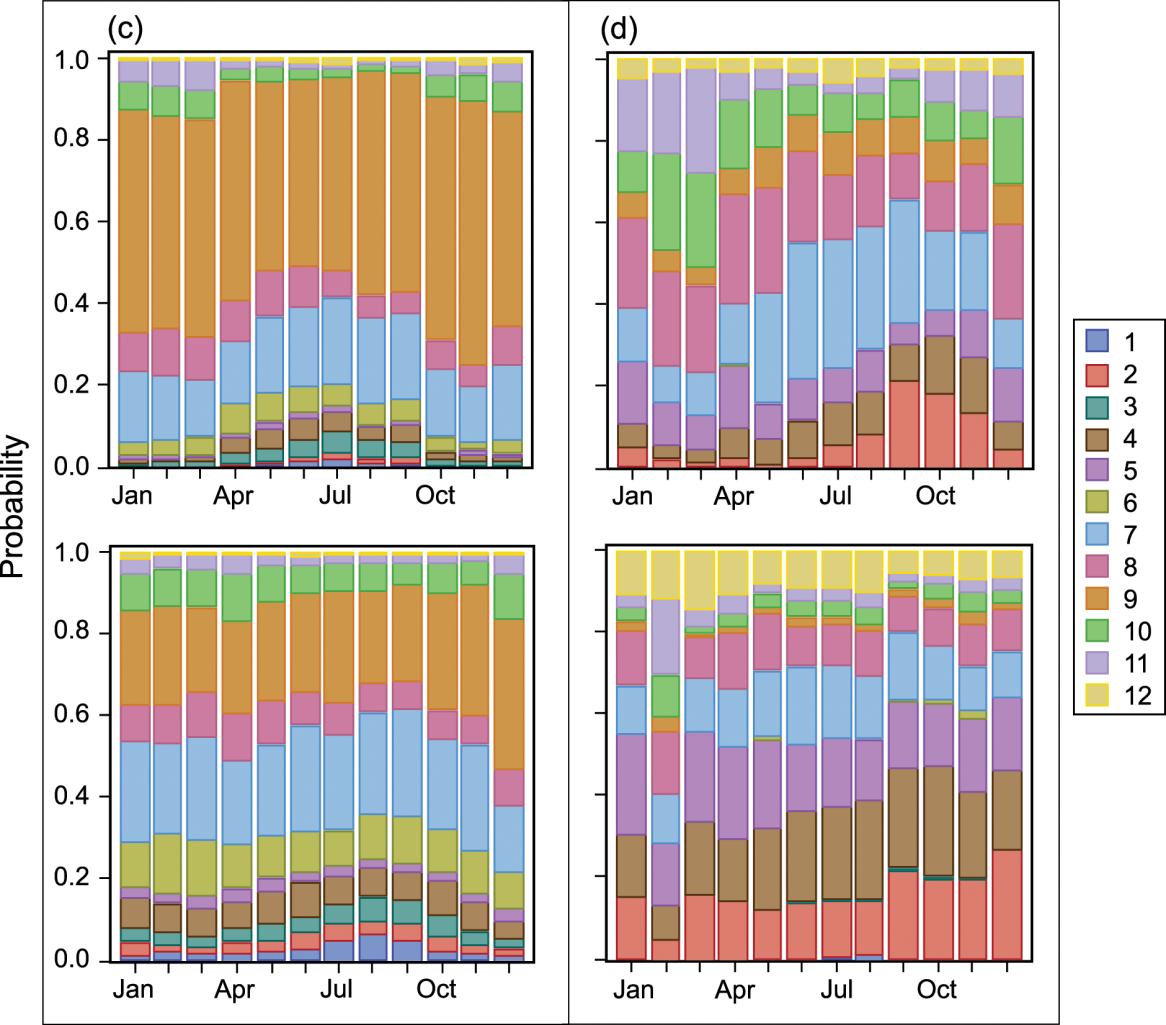
P(region of first set | month) [Upper panel] and P(choice of subsequent region | month). Upper panels: Vessels categorized by carrying capacity, departure country, and vessel dolphin mortality limit: (c) 1,050–1,250 t, PAN, COL, or VEN, Yes; and (d) 1,250–1,800 t, ECU, No. Lower panels: Vessels were categorized by carrying capacity and vessel dolphin mortality limit: (c) 1,050–1,250 t, Yes; and (d) 1,250–1,800 t, No.

As pointed out above, weather and ocean conditions influence location and timing of purse-seine fishing and fishing effort through the course of a year. Fig 8 and Fig 9 illustrate this point by showing fisher preference for a first set region and the regions to which they switch during various months and by cumulative distance travelled since departing port.

Given that modeled choices depend on both variation between fishing strategies and variations within trips, it is not surprising that while n is higher for each sub-result in the switching model (min = 1,574), the goodness of fit measures are consistently lower than in the first set model. Comparing the goodness of fit by DML/vessel-size grouping, most measures are lower by 0.1–0.2 points for vessels without DMLs and 0.2–0.3 points for vessels with DMLs. This is not because fit is poor for vessels with DMLs in the switching model, but rather because fit was so much better for vessels with DMLs in the first set model. It appears that vessels with DMLs are more homogeneous in first set strategies than they are in subsequent set decisions. Looking at all appropriately calibrated measures, the switching model still explains about 60% of the variation in the within-sample simulation for all DML/vessel size groups and frequently explains from 70% and up to 100% of variation in switching. The discrete response profile for the switching region behavior clearly shows that fishers spread out much more after their first set. The clustering of observations around coastal regions near ports is much less pronounced and sets are distributed more evenly across regions. Nevertheless, the patterns extrapolated from the first set results are reinforced by the switching analysis. Vessels without DMLs still tend to move west and south from coastal regions and vessels with DMLs largely fish either in northern or west-central regions. Smaller vessels tend to stay in coastal regions and larger vessels are more likely to fish in regions farther from the coast.

### First Set Location Choice Model

While goodness of fit and predictive capacity measures suggest that the sequential random utility model approach is quite promising, examination of parameter estimates reveals several areas for improvement and provides some insight into individual fisher behavior. Details of first set estimation results are given in Table A in S1 Appendix. While it is not possible to show and discuss herein each parameter estimate, several important elements stand out. First, distance is clearly important in decisions. Distant_Expected is strongly negative (< -2) and significant in all DML/vessel-size groupings, and it is of higher magnitude for the two smaller vessel size groups than the two larger. This reinforces the importance of expected distance to a region and supports the tendency of smaller vessels to set first in coastal regions. Similarly, in all DML/vessel-size groupings the estimates of zonal coefficients for distance traveled since departure (Distant_sinceDep) are statistically significant and strongly negative for all regions except those farthest from port locations (regions 1 and 2). Region 1 (far NW) also has a statistically significant negative coefficient for extra-large vessels fishing with a DML and the region 2 (far W) coefficient is significant and strongly negative for vessels fishing without a DML. Within the set of significant coefficients for DML vessels, those for regions closest to ports (*r* = 6, 8, and 9) are least likely to be the location of the first set when distance traveled is high. This makes considerable sense because these regions are most proximate to port locations (Fig 5).

On the other hand, the variables travel_sinceDep and search_sinceDep are much less consistent and also of lower magnitude generally (< 1). Medium size vessels with DMLs are more likely to go to regions 2, 4, 6, 7, 8 and 10 when they spend more time traveling, and extra-large vessels with DMLs are more likely to place their first set in regions 2 and 4 as travel time increases. Large vessels are less likely to place their first set in regions 5, 7, 9 and 11 as travel time increases. Hypothetically, this indicates that larger vessels sometimes travel a distance from port before making a set. This is consistent with what we know of medium to extra-large purse- seine vessels, which sometimes travel out to well-known fishing regions, forgoing sets on smaller schools encountered on the way.

There is general support for the strategy of searching regions near shore first and then moving to more distant regions. Given that these two strategies, travel far and return vs. search close and move out, are somewhat contradictory, they may cancel each other out in some segments of the data, creating the inconsistent results observed here. It is also possible that the Distant_sinceDep variable is crowding out these two variables, although the correlation coefficients between the three are low (the highest is less than 0.2).

There may also be some interaction between distance measures and proxies for the expected benefits of each region. CPUE with and without DMLs are both significant for small vessels, but while RPUE is positively correlated with the decision to place a first set in a given region, CPUE has a negative sign. Reasons for this are unclear, but may include a tradeoff between school size and species composition or some aspect of the geographic distribution of school size, particularly location of smaller schools in coastal regions.

Oceanographic variables are also consistent with some of our expectations even though some variables are insignificant for one group of observations but are significant for the other group of observations. In general, winter conditions (Oct-Mar) are more significant for vessels carrying DMLs, which avoid northeast regions in the winter and are more likely to fish in southeastern waters during this season. However, regions are not consistently significant across size groups, and there are no significant regions for small vessels, which stay near shore most of the time regardless of the season. For non-DML vessels, the coefficient for Oct_Mar is only significant in a handful of regions, but for several the sign was opposite our expectation. The MEI follows a similar pattern, though it seems to generally reduce the likelihood of placing a set in most regions for small, medium, and large vessels with DMLs, and it significantly decreases the probability of placing a first set in western regions for extra-large vessels with DMLs. The oxygen minimum layer parameters (O2_DOL_L and O2_DOL_H) are the region-specific oceanographic variables that are most consistently significant and are negative and of larger magnitude than other such parameters. However, balancing spatial scale with data requirements for these models makes linking spatially-explicit oceanographic conditions to fisher decisions very difficult.

### Switching Region Choice Model

Details of the switching region choice model and variables are laid out in Table B in S2 Appendix. This model includes trip-level variables and is more difficult to interpret than the first-step model, because it covers a much wider array of decisions, i.e. all set choices after the first set. Trip-level variables help to capture several decision factors, including the tendency to move closer to final port locations when catch on board is high; and the tendency to switch to a new region if no fish are found after expending high levels of search effort or if large schools are found but heavily fished (high catch since last region). Estimated parameters vary widely across the DML/size groups. Only Distance_Expected (how far the region is from port of landing) and Distance_Arrive (how far the fisher would need to travel to reach the new region) are consistently significant and of the expected sign for all results.

In contrast, for vessels fishing with a DML, a longer distance traveled almost uniformly results in a reduced probability of switching among regions. Results for this variable are not significant for small vessels, which suggests a uniform strategy of fishing close to port, while most region estimates are significant for other size classes. Here again, extra-large vessels diverge from the pattern in that there is a positive and significant probability that they will switch to region 1 (the far northwest) with longer distance traveled. It is possible that this is also caused by the influence of departures from ports in the western-central Pacific.

The variable search_sinceDep also shows that there are different strategies depending on vessel size and DML. For vessels without DMLs, longer search times result in a strong probability of switching to regions adjacent to the Ecuadorian port of Guayaquil (regions 9, 10, and 11), where major SKJ processing facilities are located. Large vessels with trips starting from ports in the western-central Pacific are also less likely to set in the far western equatorial region and extra-large vessels are more likely to set in central equatorial regions or in region 1, again reflecting the influence of departures from ports in the western-central Pacific. For vessels fishing with DMLs, increases in total search time result in a higher probability of switching into any region, although regions near ports (3, 6, 9 and 10) are clearly preferred. For medium and large-sized vessels the results are significant for all regions except 1 and 2, those regions farthest from continental ports of unloading. For small vessels with DMLs, none of the region coefficients are significant and signs are reversed for a few regions. Again, this is likely due to their strategy of fishing close to their home port locations.

The effect of travel_sinceDep is generally much smaller than search_sinceDep (most coefficients are <1). Small vessels without DMLs that spend more accumulated trip time traveling are less likely to set in western equatorial regions but more likely to set in region 3, which is the northern central region. This is well outside the usual fishing grounds for FADs and closer to the Mexican ports of Mazatlán and Manzanillo, where much of the YFT is landed and processed. Since vessels without a DML sometimes land large amounts of YFT taken while targeting SKJ, they may be willing to travel to these ports to get a better price. Similarly, larger vessels that travel more are more likely to set in region 6, the northeastern region, which is also near Mexican ports (Manzanillo is right on the border between the regions). However, medium-sized vessels are more likely to set in regions 9 and 10 near Guayaquil, Ecuador, and extra-large vessels are less likely to set in regions 2 or 8, again reflecting the presence of a western Pacific port for this group.

For vessels with DMLs, travel_sinceDep is much more difficult to interpret. No region coefficients are significant for small vessels (though all are positive), most are significant for medium-sized vessels, most are negative and some are significant for large vessels, and most are positive for extra-large vessels, but only significant for regions 4 and 7 in the north-central equatorial region. The results for small vessels fits their coastal strategy but the results for larger size classes can only be explained by the composition of the fleets, as the medium and extra-large categories tend to be dominated by vessels targeting SKJ on FADs but harvesting YFT incidentally, while the larger category is dominated by Mexican vessels explicitly targeting YFT in association with dolphin.

CPUE and RPUE indicators vary considerably between groups. These variables are only significant for small and extra-large vessels without DMLs and extra-large vessels with DMLs. CPUE is also significant for large vessels with DMLs. We expect that both are positive proxies for expected fishing success in a region; however, in all groups where the variable is significant, CPUE is positive and RPUE is negative. This lends support for the idea that fishers may be more likely to make switching decisions based on expected catch than on expected revenue, which would be reasonable given that prices are notoriously volatile in the fishery.

The overlap between fleets using the different métiers shows up in the effects of accumulated catch by species. Given the location of the statistically significant zones, it is possible that SKJ_sinceDep variable is picking up schools encountered on return to port, along with specific decisions to switch from one region to another.

YFT is targeted in the dolphin fishery but is bycatch in the FAD fishery, so results for accumulated YFT catches (YFT_sinceDep) are expected to be less clear for the non-DML fleet than results for the fleet with DMLs. For small vessels without DMLs, results suggest that fishers may change strategies if they happen upon a large school of YFT—either unassociated or in association with floating objects—to search for more YFT in coastal regions. However, large vessels without DMLs are more likely to switch to the far west equatorial region, so not all groups fit the pattern. Vessels with DMLs show similar behaviors to the SKJ_sinceDep results. For small, large, and extra-large vessels, most estimates (except region 11) are positive. Most estimates are not significant for small (all except 1) and extra-large size classes (all except 6 and 9). For large vessels, estimates are significant in northern regions, northern equatorial regions, and coastal regions except for the southern region.

Accumulated bigeye tuna catch (BET_sinceDep) is somewhat hit or miss for vessels with a DML, but for all size classes, higher amounts of bigeye onboard are associated with a higher probability of switching to western regions and a lower probability of switching to coastal regions. Since most bigeye is harvested as bycatch in the FAD fishery, it is possible that this reflects a higher degree of mixing in western schools vs. coastal schools, rather than any specific fishing strategy. This pattern holds for small vessels with DMLs but the coefficient on the variable is uniformly negative and significant except for region 5 for all other size classes with DMLs.

Estimates for trip-specific variables from the last region visited can also be important short-run determinants of set location choice. For vessels without a DML, parameters for the variable search_LastRegion generally point to an increased probability of switching to central equatorial or southern coastal regions and a reduced probability of switching to northern regions.

The number of sets by set-type in the last region should also be understood in the context of targeting, with the expectation that most non-DML vessels are primarily interested in schools associated with floating objects like FADs (OBJ_Last), and DML vessels should primarily be interested in schools associated with dolphins (DEL_Last). With these variables, as well as the catch since last region variables described below, one might expect that fishers are more likely to stay in a region where they have been successful, but it is important to remember that once large schools are harvested, fishers are likely to move on. Furthermore, high numbers of sets may indicate that schools in a region are small, and therefore less desirable, particularly for larger vessels. In addition, it is likely that these variables will behave differently by vessel size, as smaller vessels fill up more quickly than larger vessels and therefore return to port more often.

With the above in mind, for small and medium size vessels without a DML, most coefficients are exceptionally large (5–13), positive, and statistically significant. For large and extra-large vessels, most are still positive, but they are smaller in magnitude (< 0.3) and fewer are significant. Larger and extra-large vessels evince an increased probability (> 4) of placing their next set in central or equatorial regions. This result is surprising given that vessels without DMLs are prohibited to make sets on dolphins. The result may reflect a problem in aligning DML/non-DML with trips. DMLs are authorized for six month periods, and if a vessel exhausts its DML before the end of a period, then the probabilities of deploying to regions and other set types changes.

Results from trips by vessels with DMLs are more consistent. For example, coefficients for DEL_Last are uniformly positive and most are significant for medium, large, and extra-large size classes. None are significant for small vessels, which again may be due to a tendency to stay in coastal regions. Coefficients for OBJ_Last are not significant except for medium-size vessels. Here most are significant but negative, suggesting a reduced probability of switching, as might be expected for this group which is dominated by vessels fishing on FADs. Lastly, almost all coefficients for NOA_Last are positive, but few are significant except those for large vessels, which show an increased probability of moving into regions near port locations.

Similarly, results for catch by species since last region are more straightforward for vessels with DMLs than vessels without DMLs. For small vessels without DMLs, larger harvests of YFT in previous regions (variable YFT_Last) are correlated with an increased probability of fishing in the northern-central region and reduced probability of fishing in equatorial regions. Medium and large vessels evince an increased probability of fishing in coastal and southern regions, while extra-large vessels are less likely to switch to any region. That said, few of these coefficients are significant and most are quite small (> 0.1). The one exception is small vessels’ increased probability of fishing in region 3, which is adjacent to ports with YFT-processing plants in Mexico. Results for BET_Last are similar in terms of magnitude and significance but reversed spatially, in that higher landings of bigeye in the last region increases the likelihood that a vessel will switch into western equatorial regions and reduces likelihood of switching into coastal regions. Coefficients for SKJ_Last show that small vessels without DMLs are more likely to switch to central equatorial and southern coastal regions with higher recent catches of SKJ. The variable is not significant for medium-sized vessels but is associated with a reduced probability of switching to coastal and southern regions for large vessels and an increased probability if fishing in far west, central equatorial, and far southern regions for extra-large vessels. Like other variables in this category, however, magnitudes are small and few variables are significant.

For vessels with DMLs, higher catches of YFT in the last region fished are positively correlated (0.2 < *θ* < 0.8) with moves to far west and western central regions (such as moving from Region 6 to Region 3 indicated in Fig 2). For larger size vessels, all coefficients are negative and most are significant, suggesting a reduced probability of switching regions of operation in general. Within the results, central equatorial and southern regions are still preferred, although this may be a process of elimination (that is, large catches are harvested in typical dolphin-rich regions in the north and so fishers then explore other regions in order to fill capacity). Bigeye harvested in the last region fished (BET_Last) is not significant for small or extra-large vessels but is associated with an increased probability of switching to far western and central equatorial regions for medium and large vessels. Coefficients for SKJ_Last are not significant for large or extra-large vessels but for small vessels there is a relatively strong (< -0.39) reduction in the likelihood of switching to north-east and central coastal regions with higher recent catches of SKJ and for medium vessels coefficients for all regions are positive and significant, with far northern and central/coastal equatorial regions preferred (> 0.07).

Oceanographic variables appear to be more important (most are statistically significant) for switching region decisions than are first set decisions, but like the first set model results, aside from the oxygen minimum layer (O2), most region-specific coefficients are very small (< 0.01). Coefficients for O2 (low or 1^st^ quartile and high or 4^nd^ quartile) are consistently significant and negative for vessels fishing without DMLs and extra-large vessels with DMLs (-0.0205 to -1.1263). Only O2_H is significant for small, medium, and large vessels with DMLs, and while the coefficient is still negative, as expected, the values are smaller (> 0.1).

For variables with much smaller magnitudes, the count of 1^st^ quartile squares for sea surface temperature (SST_DEL_L) is significant for small, medium, and extra-large vessels without DMLs and is positive for small and medium vessels but negative for extra-large vessels. Coefficients for the count of 4^th^ quartile squares for SST (SST_DEL_H) are significant and negative for small and medium vessels without DMLs and for small, medium, and large vessels with DMLs. For sea surface height, SSH_DEL_L is negative and significant for all vessel size classes except small vessels with DMLs, where it is negative but not significant. Coefficients for SSH_DEL_H are consistently positive and significant across all DML/size groups. Both the upper and lower bounds on mixed layer depth are negative for all vessels without DMLs but are not statistically significant for medium-sized vessels and MLD_DEL_L is not significant for large vessels. MLD_DEL_L is positive for all vessels with DMLs, but only significant for the medium size class. Chlorophyll is less in line with expectations. CHLORO_DEL_L is positive and significant for all vessels without DMLs and for large vessels with DMLs, but is otherwise insignificant. CHLORO_DEL_H is significant but positive for large vessels without DMLs and significant and negative for medium vessels with DML.

MEI is generally associated with reduced probability of moving into far western equatorial regions and increased probability of staying in coastal regions, though less than half of the region coefficients are statistically significant for any of the non-DML results. Effects are greater than other oceanographic indicators (|0.3–1.28|). For small vessels with a DML, there is a reduced probability of moving to northern central and central equatorial regions, while for larger size classes, most coefficients are negative and significant, suggesting a general reduced probability of switching regions.

## Discussion

In general, goodness of fit measures show that the sequential random utility model explains much of the observed variation for decisions made prior to vessel departure from port and for decisions made daily on vessel operations. The predictive capacity is very good. It is generally known that there has been no reliable means to predict fisherman behavior in response to changes in environment or management or the changing conditions aboard a vessel at sea, but the model we have developed was able at levels over 30% to correctly predict skipper decisions and vessel movements. There were however some difficulties in the interpretation of the estimated coefficients that suggest that refinement of the model may be possible.

Our results show that the sequential random utility model can be a useful tool for understanding and predicting spatial decision processes in large-scale commercial fisheries, where alternative choices for set location are not temporally independent. This is a novel approach, and based on our results, we see considerable opportunity for further work and development. Although we selected each variable in the model to carefully reflect a different aspect of fisher choices, it is possible that the model is over-specified and that tests of alternate models could be undertaken to find the minimum necessary set of independent variables. With fewer variables we may also be able to delineate smaller choice regions. In particular, all variables that do not vary relatively widely across regions take up *R* degrees of freedom, so minimizing the number of such variables would provide considerable advantage in terms of fit vs. resolution. This issue will be discussed further below.

The model also captured the primary trip strategies used by purse-seiners in the EPO. These include 1) the tendency of small vessels to stay in coastal regions regardless of DML status—even though they have the capacity to travel longer distances, 2) the central equatorial and southern search patterns evinced by larger vessels without a DML, and 3) the northern search pattern used by medium, large, and extra-large vessels fishing with DMLs. On the other hand, there was some confounding of results based on the overlap of fisher strategies. This was seen for some vessels with DMLs targeting fish in association with FADs, resulting in a behavior differing from most vessels with a DML that primarily target YFT in association with dolphin. In future work, it would be useful to group fishers by flag state (or country of origin) as well as DML, because targeting behavior appears to be strongly associated with flag. Breaking down the dependent variable by flag groups would also help to tease out differences between fishers based on port of origin and could be useful for looking at response to future political and economic behavior of tuna commissions resulting from changes in fuel prices, regulations, and climate.

The results also clearly show that distance is a good proxy for costs and is one of the most important factors in fisher decision processes. Both expected distance and distance traveled since leaving port are strongly significant and of the expected sign. Other variables based on search and travel data are less clear indicators, possibly due to interactions with distance. A full analysis is outside the scope of this paper, but initial tests of the model without the Distant_sinceDep variable show that there is not much reduction in goodness of fit and that travel and search indicators have higher levels of significance and fit expectations more often. This indicates that there is a level of confounding, even though the variables are not strongly correlated. It is likely that the inclusion of both variables catch-by-species and number of sets-by-set type is similarly confounding the effects of these variables.

Increasing the number of regions, and thereby reducing region size, would increase the spatial resolution of the model and may improve the estimates for oceanographic conditions in particular. In spite of the fact that fishers often use satellite imagery to plan their trips and determine search locations, region-specific oceanographic measures were the least well-behaved and had the smallest magnitude of impact in the model. This was not surprising because vessel operational response is to the local environment. Although we attempted to compensate for the large size of the regions by using the lower and upper bounds of the preferred fishing conditions as proxies, this clearly was not sufficient to capture the effect of oceanographic conditions. In addition to smaller regions, a central measure such as the percent of squares within one standard deviation from the mean would reduce the total number of variables and provide a clearer indication of fishers’ preferred oceanographic conditions. It would also be helpful to examine the usefulness of each indicator, as some, like the oxygen minimum layer and mixed layer depth, tend to be correlated. Indeed, O2 was the only variable that was fairly consistent with expectations within this group of explanatory variables. Furthermore, it is possible that some variables, like SST, are positively correlated with location, so this is another region where testing of specification is needed.

With this first version of the sequential random utility model completed, it would be useful to develop versions that are not as data-dependent. Although use of on-board observers and vessel monitoring systems is increasing the availability of high resolution spatial data, the IATTC dataset used here is highly detailed, and variables like “search” and “travel” time are not likely to be available to most fisheries management organizations. Indeed, many agencies may only have access to effort at weekly or monthly time-scales, so the daily modeling described here would not be feasible. Given these limits, the results presented here suggest that distance can serve as a good proxy for travel and search and that a middle-range in terms of temporal and spatial scale might be desirable.

Ironically, another region of major concern is actually the lack of data points. Although this dataset is highly detailed, the fleet is quite small relative to the region fished, and a higher resolution model is likely to return a large number of empty sets. Aggregation over time rather than space has been used to solve this problem [10], but this has costs in terms of information lost through aggregation. It also presents problems in situations where time crosses significantly different environmental conditions [34]. One alternative is to use a combination of statistical models to calibrate and test agent-based models of fishing behavior that include adaptive decision processes. Such models tend to be highly complex and difficult to interpret but may be necessary when predicting future expectations with models such as the sequential random utility model.

In spite of the potential issues described herein, we believe that this model is the first step to a better understanding of spatial dynamics in large-scale fisheries and may have uses in applications other than marine resource management. Our work provides clear evidence of spatially-explicit patterns of exploitation that could be refined to provide better understanding of human decision processes that can have profound effects on ecosystems. While studying small-scale systems is more tractable, in larger systems, collective decisions such as where to fish, hunt, mine, or pollute can have much broader impacts. Furthermore, in these contexts, space-based management is increasingly important. Most correctly believe that the success of this approach to resource management depends heavily on the responses of individuals as they change their resource use strategies to accommodate new regulations, but spatial decisions also come into play much earlier in the process, as political and economic issues shape the placement of time-area closures, marine protected regions, and related space-based management measures. These are but a few of the reasons that there is a need to continue to develop and improve modeling techniques like the sequential random utility model.

## Supporting Information

S1 Appendix Table A. Estimation and goodness of fit result for the first set location choice model of the tuna purse seine fleet in eastern Pacific Ocean by DML and vessel size.
(PDF)

S2 Appendix Table B. Estimation and goodness of fit result for the switching region choice model of the tuna purse seine fleet in eastern Pacific Ocean by DML and vessel size.
(PDF)

S3 Appendix Fig A. Observed and predicted choices of first set location model for each region.
(PDF)

S4 Appendix Fig B. Observed and predicted choices of switching region choice model for each region.
(PDF)
