# Supplementary material for: Modeling the Spatial Dynamics of International Tuna Fleets
Source: PLoS One. 2016 Aug 18;11(8):e0159626. doi: 10.1371/journal.pone.0159626 (PMC4990267; doi:10.1371/journal.pone.0159626)
Supplement: S1 Appendix — Table A. Estimation and goodness of fit result for the first set location choice model of the tuna purse seine fleet in eastern Pacific Ocean by DML and vessel size. (PDF) [file pone.0159626.s001.pdf]

Table A. Estimation and goodness of fit result of the first set region sequential random utility model of the tuna purse seine fleet in eastern Pacific Ocean by DML and vessel size

Conditional Logit Estimates

DML=0 Vessel_Size=1_Small (363-700t)

Algorithm converged.

Model Fit Summary	
Dependent Variable	Decision
Number of Observations	1927
Number of Cases	23124
Log Likelihood	-2594
Log Likelihood Null (LogL(0))	-4788
Maximum Absolute Gradient	10.85993
Number of Iterations	195
Optimization Method	Dual Quasi-Newton
AIC	5324
Schwarz Criterion	5702

Discrete Response Profile			
Index	CHOICE	Frequency	Percent
0	1	1	0.05
1	2	2	0.10
2	3	28	1.45
3	4	30	1.56
4	5	40	2.08
5	6	2	0.10
6	7	245	12.71
7	8	327	16.97
8	9	353	18.32
9	10	687	35.65
10	11	174	9.03
11	12	38	1.97

Table A (cont). Estimation and goodness of fit result of the first set region sequential random utility model of the tuna purse seine fleet in eastern Pacific Ocean by DML and vessel size

Conditional Logit Estimates

DML=0 Vessel_Size=1_Small (363-700t)

Goodness-of-Fit Measures		
Measure	Value	Formula
Likelihood Ratio (R)	4388.9	2 * (LogL - LogL0)
Upper Bound of R (U)	9576.8	- 2 * LogL0
Aldrich-Nelson	0.6949	R / (R+N)
Cragg-Uhler 1	0.8975	1 - exp(-R/N)
Cragg-Uhler 2	0.9037	(1-exp(-R/N)) / (1-exp(-U/N))
Estrella	0.9525	1 - (1-R/U)^(U/N)
Adjusted Estrella	0.946	1 - ((LogL-K)/LogL0)^(-2/N*LogL0)
McFadden's LRI	0.4583	R / U
Veall-Zimmermann	0.8347	(R * (U+N)) / (U * (R+N))

N = # of observations, K = # of regressors

Table A Estimation and Goodness of Fit Result of the First Set Region SRUM by DML and Vessel Size of Tuna Purse Seine Fleet in EPO

Conditional Logit Estimates

DML=0 Vessel_Size=1_Small (363-700t)

Parameter Estimates

Parameter	DF	Estimate	Standard Error	t Value	Approx Pr > |t|
LDistant_Expected	1	-3.3648	0.2642	-12.74	<.0001
lCPUE_All_1	1	-0.3003	0.1172	-2.56	0.0104
lRPUE_All_1	1	0.2401	0.0764	3.14	0.0017
LDistant_sinceDep1_z1	1	-0.6654	1.5398	-0.43	0.6656
LDistant_sinceDep1_z2	1	-0.3730	0.2686	-1.39	0.1649
LDistant_sinceDep1_z3	1	-1.9219	0.5621	-3.42	0.0006
LDistant_sinceDep1_z4	1	-3.3166	0.4508	-7.36	<.0001
LDistant_sinceDep1_z5	1	-2.8443	0.3735	-7.62	<.0001
LDistant_sinceDep1_z6	1	-3.1214	0.6690	-4.67	<.0001
LDistant_sinceDep1_z7	1	-2.9744	0.4282	-6.95	<.0001
LDistant_sinceDep1_z8	1	-3.1476	0.4460	-7.06	<.0001
LDistant_sinceDep1_z9	1	-3.6097	0.4719	-7.65	<.0001
LDistant_sinceDep1_z10	1	-3.7412	0.4745	-7.89	<.0001
LDistant_sinceDep1_z11	1	-3.0114	0.4777	-6.30	<.0001
LDF_travel_sinceDep1_z1	1	-0.0265	1.9830	-0.01	0.9893
LDF_travel_sinceDep1_z2	1	0.0297	0.5190	0.06	0.9543
LDF_travel_sinceDep1_z3	1	-0.0936	0.1970	-0.48	0.6346
LDF_travel_sinceDep1_z4	1	0.0135	0.2103	0.06	0.9488
LDF_travel_sinceDep1_z5	1	0.0709	0.2004	0.35	0.7235
LDF_travel_sinceDep1_z6	1	1.5452	0.7152	2.16	0.0307
LDF_travel_sinceDep1_z7	1	-0.0460	0.1557	-0.30	0.7674
LDF_travel_sinceDep1_z8	1	-0.0568	0.1556	-0.36	0.7151
LDF_travel_sinceDep1_z9	1	-0.1471	0.1550	-0.95	0.3425
LDF_travel_sinceDep1_z10	1	-0.1703	0.1547	-1.10	0.2710
LDF_travel_sinceDep1_z11	1	-0.1098	0.1574	-0.70	0.4854
LDF_search_sinceDep1_z1	1	0.1049	0.1948	0.54	0.5902
LDF_search_sinceDep1_z2	1	0.6324	1.9935	0.32	0.7511
LDF_search_sinceDep1_z3	1	0.5839	1.7395	0.34	0.7371
LDF_search_sinceDep1_z4	1	3.7610	1.6211	2.32	0.0203
LDF_search_sinceDep1_z5	1	2.8817	1.4086	2.05	0.0408

Table A Estimation and Goodness of Fit Result of the First Set Region SRUM by DML and Vessel Size of Tuna Purse Seine Fleet in EPO

Conditional Logit Estimates

DML=0 Vessel_Size=1_Small (363-700t)

Parameter Estimates

Parameter	DF	Estimate	Standard Error	t Value	Approx Pr > |t|
LDF_search_sinceDep1_z6	1	0.9133	1.7727	0.52	0.6064
LDF_search_sinceDep1_z7	1	1.0063	0.9922	1.01	0.3105
LDF_search_sinceDep1_z8	1	1.2490	0.8130	1.54	0.1244
LDF_search_sinceDep1_z9	1	0.3034	0.6386	0.48	0.6347
LDF_search_sinceDep1_z10	1	0.1146	0.4882	0.23	0.8144
LDF_search_sinceDep1_z11	1	0.8465	0.4094	2.07	0.0386
SST_DOL_L	1	0.008530	0.001339	6.37	<.0001
SST_DOL_H	1	-0.0200	0.002829	-7.07	<.0001
SSH_DOL_L	1	-0.000988	0.001729	-0.57	0.5676
SSH_DOL_H	1	0.003724	0.002030	1.83	0.0665
MLD_DOL_L	1	0.003076	0.002654	1.16	0.2464
MLD_DOL_H	1	-0.009058	0.001398	-6.48	<.0001
CHLORO_DOL_L	1	0.0106	0.001692	6.24	<.0001
CHLORO_DOL_H	1	-0.007038	0.002722	-2.59	0.0097
O2_DOL_L	1	-0.1050	0.0291	-3.60	0.0003
O2_DOL_H	1	-0.0599	0.007692	-7.78	<.0001
Oct_Mar_z1	1	-0.2499	3.4687	-0.07	0.9426
Oct_Mar_z2	1	-0.7056	1.4806	-0.48	0.6337
Oct_Mar_z3	1	0.3489	0.8223	0.42	0.6713
Oct_Mar_z4	1	-0.4503	0.5528	-0.81	0.4153
Oct_Mar_z5	1	0.0170	0.5229	0.03	0.9741
Oct_Mar_z6	1	-1.0690	1.6925	-0.63	0.5276
Oct_Mar_z7	1	-0.7946	0.4238	-1.88	0.0608
Oct_Mar_z8	1	-0.0989	0.4124	-0.24	0.8104
Oct_Mar_z9	1	-1.0623	0.4251	-2.50	0.0125
Oct_Mar_z10	1	-0.2885	0.4119	-0.70	0.4836
Oct_Mar_z11	1	0.3038	0.4241	0.72	0.4739
MEI_z1	1	-0.2093	4.2622	-0.05	0.9608
MEI_z2	1	-0.0640	1.0112	-0.06	0.9495
MEI_z3	1	0.3702	0.4242	0.87	0.3828

Table A Estimation and Goodness of Fit Result of the First Set Region SRUM by DML and Vessel Size of Tuna Purse Seine Fleet in EPO

Conditional Logit Estimates

DML=0 Vessel_Size=1_Small (363-700t)

Parameter Estimates

Parameter	DF	Estimate	Standard Error	t Value	Approx Pr > |t|
MEI_z4	1	-0.5293	0.2992	-1.77	0.0769
MEI_z5	1	-0.5715	0.2765	-2.07	0.0388
MEI_z6	1	1.0014	0.8280	1.21	0.2265
MEI_z7	1	-0.2479	0.2119	-1.17	0.2421
MEI_z8	1	-0.2710	0.2162	-1.25	0.2100
MEI_z9	1	0.1277	0.2119	0.60	0.5468
MEI_z10	1	-0.1299	0.2106	-0.62	0.5374
MEI_z11	1	-0.2150	0.2194	-0.98	0.3271

Table A Estimation and Goodness of Fit Result of the First Set Region SRUM by DML and Vessel Size of Tuna Purse Seine Fleet in EPO

Conditional Logit Estimates

DML=0 Vessel_Size=2_Median (700-1,050t)

Algorithm converged.

Model Fit Summary	
Dependent Variable	Decision
Number of Observations	985
Number of Cases	11820
Log Likelihood	-1485
Log Likelihood Null (LogL(0))	-2448
Maximum Absolute Gradient	5.77839
Number of Iterations	214
Optimization Method	Dual Quasi-Newton
AIC	3106
Schwarz Criterion	3439

Discrete Response Profile

Index	CHOICE	Frequency	Percent
0	1	0	0.00
1	2	3	0.30
2	3	5	0.51
3	4	17	1.73
4	5	47	4.77
5	6	1	0.10
6	7	157	15.94
7	8	227	23.05
8	9	157	15.94
9	10	221	22.44
10	11	92	9.34
11	12	58	5.89

Table A Estimation and Goodness of Fit Result of the First Set Region SRUM by DML and Vessel Size of Tuna Purse Seine Fleet in EPO

Conditional Logit Estimates

DML=0 Vessel_Size=2_Median (700-1,050t)

Goodness-of-Fit Measures		
Measure	Value	Formula
Likelihood Ratio (R)	1925.4	2 * (LogL - LogL0)
Upper Bound of R (U)	4895.3	- 2 * LogL0
Aldrich-Nelson	0.6616	R / (R+N)
Cragg-Uhler 1	0.8584	1 - exp(-R/N)
Cragg-Uhler 2	0.8644	(1-exp(-R/N)) / (1-exp(-U/N))
Estrella	0.9166	1 - (1-R/U)^(U/N)
Adjusted Estrella	0.8958	1 - ((LogL-K)/LogL0)^(-2/N*LogL0)
McFadden's LRI	0.3933	R / U
Veall-Zimmermann	0.7947	(R * (U+N)) / (U * (R+N))
N = # of observations, K = # of regressors		

Table A Estimation and Goodness of Fit Result of the First Set Region SRUM by DML and Vessel Size of Tuna Purse Seine Fleet in EPO

Conditional Logit Estimates

DML=0 Vessel_Size=2_Median (700-1,050t)

Parameter Estimates

Parameter	DF	Estimate	Standard Error	t Value	Approx Pr > |t|
LDistant_Expected	1	-6.0489	0.6432	-9.40	<.0001
lCPUE_All_1	1	0.0965	0.1533	0.63	0.5291
lRPUE_All_1	1	-0.0461	0.0977	-0.47	0.6371
LDistant_sinceDep1_z1	1	-2.1346	818.1237	-0.00	0.9979
LDistant_sinceDep1_z2	1	-0.1659	0.3741	-0.44	0.6575
LDistant_sinceDep1_z3	1	-1.0323	0.7163	-1.44	0.1495
LDistant_sinceDep1_z4	1	-2.4404	0.3750	-6.51	<.0001
LDistant_sinceDep1_z5	1	-2.1560	0.3078	-7.00	<.0001
LDistant_sinceDep1_z6	1	-4.3905	1.6957	-2.59	0.0096
LDistant_sinceDep1_z7	1	-2.3306	0.3292	-7.08	<.0001
LDistant_sinceDep1_z8	1	-2.4499	0.3446	-7.11	<.0001
LDistant_sinceDep1_z9	1	-3.2884	0.3634	-9.05	<.0001
LDistant_sinceDep1_z10	1	-3.5302	0.3682	-9.59	<.0001
LDistant_sinceDep1_z11	1	-2.2904	0.3937	-5.82	<.0001
LDF_travel_sinceDep1_z1	1	0.3934	2620	0.00	0.9999
LDF_travel_sinceDep1_z2	1	0.8326	0.5619	1.48	0.1384
LDF_travel_sinceDep1_z3	1	0.7051	0.7994	0.88	0.3778
LDF_travel_sinceDep1_z4	1	-0.0150	0.1570	-0.10	0.9241
LDF_travel_sinceDep1_z5	1	0.4046	0.1893	2.14	0.0326
LDF_travel_sinceDep1_z6	1	-0.0364	1.5946	-0.02	0.9818
LDF_travel_sinceDep1_z7	1	0.0626	0.0877	0.71	0.4757
LDF_travel_sinceDep1_z8	1	0.0236	0.0850	0.28	0.7811
LDF_travel_sinceDep1_z9	1	-0.0269	0.0862	-0.31	0.7546
LDF_travel_sinceDep1_z10	1	-0.0457	0.0857	-0.53	0.5936
LDF_travel_sinceDep1_z11	1	-0.0376	0.0909	-0.41	0.6792
LDF_search_sinceDep1_z1	1	-0.2004	317.2243	-0.00	0.9995
LDF_search_sinceDep1_z2	1	-2.3468	3172	-0.00	0.9994
LDF_search_sinceDep1_z3	1	-2.3530	2855	-0.00	0.9993
LDF_search_sinceDep1_z4	1	0.5570	2538	0.00	0.9998
LDF_search_sinceDep1_z5	1	0.9097	2221	0.00	0.9997

Table A Estimation and Goodness of Fit Result of the First Set Region SRUM by DML and Vessel Size of Tuna Purse Seine Fleet in EPO

Conditional Logit Estimates

DML=0 Vessel_Size=2_Median (700-1,050t)

Parameter Estimates

Parameter	DF	Estimate	Standard Error	t Value	Approx Pr > |t|
LDF_search_sinceDep1_z6	1	7.4219	1903	0.00	0.9969
LDF_search_sinceDep1_z7	1	-0.7674	1586	-0.00	0.9996
LDF_search_sinceDep1_z8	1	-0.3982	1269	-0.00	0.9997
LDF_search_sinceDep1_z9	1	-0.4936	951.6729	-0.00	0.9996
LDF_search_sinceDep1_z10	1	-0.3358	634.4486	-0.00	0.9996
LDF_search_sinceDep1_z11	1	0.5716	317.2244	0.00	0.9986
SST_DOL_L	1	0.003502	0.001440	2.43	0.0150
SST_DOL_H	1	-0.0163	0.003357	-4.87	<.0001
SSH_DOL_L	1	-0.003368	0.002184	-1.54	0.1231
SSH_DOL_H	1	0.006939	0.002563	2.71	0.0068
MLD_DOL_L	1	-0.004366	0.003531	-1.24	0.2162
MLD_DOL_H	1	-0.001087	0.001824	-0.60	0.5515
CHLORO_DOL_L	1	0.009344	0.002097	4.46	<.0001
CHLORO_DOL_H	1	-0.004308	0.003851	-1.12	0.2634
O2_DOL_L	1	-0.1092	0.0511	-2.14	0.0327
O2_DOL_H	1	-0.0497	0.007064	-7.04	<.0001
Oct_Mar_z1	1	-0.0453	4642	-0.00	1.0000
Oct_Mar_z2	1	-0.4273	1.3995	-0.31	0.7601
Oct_Mar_z3	1	-6.0121	2.9191	-2.06	0.0394
Oct_Mar_z4	1	-0.4994	0.6005	-0.83	0.4056
Oct_Mar_z5	1	-0.0550	0.4703	-0.12	0.9070
Oct_Mar_z6	1	-4.5740	5.4675	-0.84	0.4028
Oct_Mar_z7	1	-0.5030	0.3836	-1.31	0.1897
Oct_Mar_z8	1	0.1393	0.3650	0.38	0.7027
Oct_Mar_z9	1	-0.4751	0.3937	-1.21	0.2275
Oct_Mar_z10	1	-0.0338	0.3829	-0.09	0.9296
Oct_Mar_z11	1	0.5091	0.3929	1.30	0.1951
MEI_z1	1	-0.0314	2390	-0.00	1.0000
MEI_z2	1	-0.9002	0.8459	-1.06	0.2872
MEI_z3	1	0.4544	0.9756	0.47	0.6414

Table A Estimation and Goodness of Fit Result of the First Set Region SRUM by DML and Vessel Size of Tuna Purse Seine Fleet in EPO

Conditional Logit Estimates

DML=0 Vessel_Size=2_Median (700-1,050t)

Parameter Estimates

Parameter	DF	Estimate	Standard Error	t Value	Approx Pr > |t|
MEI_z4	1	-0.4516	0.3171	-1.42	0.1544
MEI_z5	1	-0.2392	0.2494	-0.96	0.3374
MEI_z6	1	-0.2567	2.3809	-0.11	0.9142
MEI_z7	1	-0.1892	0.1887	-1.00	0.3160
MEI_z8	1	-0.0789	0.1965	-0.40	0.6879
MEI_z9	1	0.0838	0.2009	0.42	0.6768
MEI_z10	1	0.0755	0.2000	0.38	0.7056
MEI_z11	1	0.0493	0.2116	0.23	0.8157

Table A Estimation and Goodness of Fit Result of the First Set Region SRUM by DML and Vessel Size of Tuna Purse Seine Fleet in EPO

Conditional Logit Estimates

DML=0 Vessel_Size=3_Large (1,050-1,250t)

Algorithm converged.

Model Fit Summary	
Dependent Variable	Decision
Number of Observations	408
Number of Cases	4896
Log Likelihood	-676.95551
Log Likelihood Null (LogL(0))	-1014
Maximum Absolute Gradient	1.32394
Number of Iterations	177
Optimization Method	Dual Quasi-Newton
AIC	1490
Schwarz Criterion	1763

Discrete Response Profile			
Index	CHOICE	Frequency	Percent
0	1	1	0.25
1	2	21	5.15
2	3	12	2.94
3	4	26	6.37
4	5	32	7.84
5	6	3	0.74
6	7	84	20.59
7	8	70	17.16
8	9	33	8.09
9	10	49	12.01
10	11	41	10.05
11	12	36	8.82

Table A Estimation and Goodness of Fit Result of the First Set Region SRUM by DML and Vessel Size of Tuna Purse Seine Fleet in EPO

Conditional Logit Estimates

DML=0 Vessel_Size=3_Large (1,050-1,250t)

Goodness-of-Fit Measures		
Measure	Value	Formula
Likelihood Ratio (R)	673.77	2 * (LogL - LogL0)
Upper Bound of R (U)	2027.7	- 2 * LogL0
Aldrich-Nelson	0.6228	R / (R+N)
Cragg-Uhler 1	0.8082	1 - exp(-R/N)
Cragg-Uhler 2	0.8139	(1-exp(-R/N)) / (1-exp(-U/N))
Estrella	0.8656	1 - (1-R/U)^(U/N)
Adjusted Estrella	0.7838	1 - ((LogL-K)/LogL0)^(-2/N*LogL0)
McFadden's LRI	0.3323	R / U
Veall-Zimmermann	0.7482	(R * (U+N)) / (U * (R+N))
N = # of observations, K = # of regressors		

Table A Estimation and Goodness of Fit Result of the First Set Region SRUM by DML and Vessel Size of Tuna Purse Seine Fleet in EPO

Conditional Logit Estimates

DML=0 Vessel_Size=3_Large (1,050-1,250t)

Parameter Estimates

Parameter	DF	Estimate	Standard Error	t Value	Approx Pr > |t|
LDistant_Expected	1	-2.8021	0.3741	-7.49	<.0001
lCPUE_All_1	1	0.1962	0.2299	0.85	0.3935
lRPUE_All_1	1	-0.0938	0.1476	-0.64	0.5252
LDistant_sinceDep1_z1	1	-1.4084	2.0373	-0.69	0.4894
LDistant_sinceDep1_z2	1	-0.6337	0.2010	-3.15	0.0016
LDistant_sinceDep1_z3	1	-2.8433	0.4766	-5.97	<.0001
LDistant_sinceDep1_z4	1	-2.3613	0.4156	-5.68	<.0001
LDistant_sinceDep1_z5	1	-2.1996	0.3574	-6.15	<.0001
LDistant_sinceDep1_z6	1	-3.6322	0.6187	-5.87	<.0001
LDistant_sinceDep1_z7	1	-2.3621	0.3943	-5.99	<.0001
LDistant_sinceDep1_z8	1	-2.3743	0.4122	-5.76	<.0001
LDistant_sinceDep1_z9	1	-3.0381	0.4342	-7.00	<.0001
LDistant_sinceDep1_z10	1	-3.0953	0.4375	-7.07	<.0001
LDistant_sinceDep1_z11	1	-2.7492	0.4582	-6.00	<.0001
LDF_travel_sinceDep1_z1	1	0.5735	1.4629	0.39	0.6951
LDF_travel_sinceDep1_z2	1	-0.0939	0.1962	-0.48	0.6322
LDF_travel_sinceDep1_z3	1	-0.3354	0.2723	-1.23	0.2180
LDF_travel_sinceDep1_z4	1	0.1297	0.2830	0.46	0.6466
LDF_travel_sinceDep1_z5	1	-0.4169	0.1882	-2.22	0.0267
LDF_travel_sinceDep1_z6	1	-0.2205	0.4062	-0.54	0.5873
LDF_travel_sinceDep1_z7	1	-0.3604	0.1729	-2.08	0.0371
LDF_travel_sinceDep1_z8	1	-0.2758	0.1765	-1.56	0.1181
LDF_travel_sinceDep1_z9	1	-0.4294	0.1817	-2.36	0.0181
LDF_travel_sinceDep1_z10	1	-0.3831	0.1783	-2.15	0.0317
LDF_travel_sinceDep1_z11	1	-0.4353	0.1824	-2.39	0.0170
LDF_search_sinceDep1_z1	1	-0.1074	0.5255	-0.20	0.8381
LDF_search_sinceDep1_z2	1	-0.8862	5.2542	-0.17	0.8661
LDF_search_sinceDep1_z3	1	-0.8399	4.7233	-0.18	0.8589
LDF_search_sinceDep1_z4	1	0.3168	4.2202	0.08	0.9402
LDF_search_sinceDep1_z5	1	1.2337	3.6968	0.33	0.7386

Table A Estimation and Goodness of Fit Result of the First Set Region SRUM by DML and Vessel Size of Tuna Purse Seine Fleet in EPO

Conditional Logit Estimates

DML=0 Vessel_Size=3_Large (1,050-1,250t)

Parameter Estimates

Parameter	DF	Estimate	Standard Error	t Value	Approx Pr > |t|
LDF_search_sinceDep1_z6	1	1.6587	3.3945	0.49	0.6251
LDF_search_sinceDep1_z7	1	-0.2955	2.6303	-0.11	0.9105
LDF_search_sinceDep1_z8	1	-0.1361	2.1105	-0.06	0.9486
LDF_search_sinceDep1_z9	1	-0.1205	1.5894	-0.08	0.9395
LDF_search_sinceDep1_z10	1	-0.0684	1.0756	-0.06	0.9493
LDF_search_sinceDep1_z11	1	1.4536	0.6878	2.11	0.0346
SST_DOL_L	1	-0.000184	0.001935	-0.10	0.9242
SST_DOL_H	1	-0.004884	0.004068	-1.20	0.2299
SSH_DOL_L	1	-0.006722	0.003327	-2.02	0.0433
SSH_DOL_H	1	0.003917	0.002868	1.37	0.1720
MLD_DOL_L	1	-0.0121	0.005534	-2.19	0.0283
MLD_DOL_H	1	-0.006833	0.002202	-3.10	0.0019
CHLORO_DOL_L	1	0.008465	0.002332	3.63	0.0003
CHLORO_DOL_H	1	0.009094	0.005545	1.64	0.1010
O2_DOL_L	1	0.000351	0.0231	0.02	0.9879
O2_DOL_H	1	-0.0427	0.007912	-5.40	<.0001
Oct_Mar_z1	1	1.5765	9.3045	0.17	0.8655
Oct_Mar_z2	1	0.7917	0.7192	1.10	0.2710
Oct_Mar_z3	1	0.4185	1.6597	0.25	0.8009
Oct_Mar_z4	1	-0.2918	0.6339	-0.46	0.6453
Oct_Mar_z5	1	0.4127	0.6081	0.68	0.4974
Oct_Mar_z6	1	-1.5932	1.5202	-1.05	0.2946
Oct_Mar_z7	1	-0.2960	0.5671	-0.52	0.6016
Oct_Mar_z8	1	0.1608	0.5305	0.30	0.7619
Oct_Mar_z9	1	-0.5458	0.6406	-0.85	0.3942
Oct_Mar_z10	1	0.0742	0.5872	0.13	0.8995
Oct_Mar_z11	1	0.8506	0.5643	1.51	0.1317
MEI_z1	1	3.8470	5.5486	0.69	0.4881
MEI_z2	1	-1.0728	0.4059	-2.64	0.0082
MEI_z3	1	0.6658	0.7013	0.95	0.3425

Table A Estimation and Goodness of Fit Result of the First Set Region SRUM by DML and Vessel Size of Tuna Purse Seine Fleet in EPO

Conditional Logit Estimates

DML=0 Vessel_Size=3_Large (1,050-1,250t)

Parameter Estimates					
Parameter	DF	Estimate	Standard Error	t Value	Approx Pr > |t|
MEI_z4	1	-0.7567	0.3268	-2.32	0.0206
MEI_z5	1	-0.4854	0.3101	-1.57	0.1175
MEI_z6	1	1.4019	0.6842	2.05	0.0405
MEI_z7	1	-0.3750	0.2746	-1.37	0.1721
MEI_z8	1	-0.3347	0.2832	-1.18	0.2372
MEI_z9	1	0.0899	0.3412	0.26	0.7923
MEI_z10	1	0.0741	0.3115	0.24	0.8119
MEI_z11	1	-0.0766	0.2854	-0.27	0.7884

Table A Estimation and Goodness of Fit Result of the First Set Region SRUM by DML and Vessel Size of Tuna Purse Seine Fleet in EPO

Conditional Logit Estimates

DML=0 Vessel_Size=4_XLarge (1,250-1,800t)

Algorithm converged.

Model Fit Summary	
Dependent Variable	Decision
Number of Observations	590
Number of Cases	7080
Log Likelihood	-911.54056
Log Likelihood Null (LogL(0))	-1466
Maximum Absolute Gradient	1.85111
Number of Iterations	170
Optimization Method	Dual Quasi-Newton
AIC	1959
Schwarz Criterion	2257

Discrete Response Profile

Index	CHOICE	Frequency	Percent
0	1	0	0.00
1	2	49	8.31
2	3	0	0.00
3	4	51	8.64
4	5	58	9.83
5	6	2	0.34
6	7	134	22.71
7	8	104	17.63
8	9	54	9.15
9	10	59	10.00
10	11	56	9.49
11	12	23	3.90

Table A Estimation and Goodness of Fit Result of the First Set Region SRUM by DML and Vessel Size of Tuna Purse Seine Fleet in EPO

Conditional Logit Estimates

DML=0 Vessel_Size=4_XLarge (1,250-1,800t)

Goodness-of-Fit Measures		
Measure	Value	Formula
Likelihood Ratio (R)	1109.1	2 * (LogL - LogL0)
Upper Bound of R (U)	2932.2	- 2 * LogL0
Aldrich-Nelson	0.6528	R / (R+N)
Cragg-Uhler 1	0.8474	1 - exp(-R/N)
Cragg-Uhler 2	0.8533	(1-exp(-R/N)) / (1-exp(-U/N))
Estrella	0.9057	1 - (1-R/U)^(U/N)
Adjusted Estrella	0.8652	1 - ((LogL-K)/LogL0)^(-2/N*LogL0)
McFadden's LRI	0.3783	R / U
Veall-Zimmermann	0.7841	(R * (U+N)) / (U * (R+N))
N = # of observations, K = # of regressors		

Table A Estimation and Goodness of Fit Result of the First Set Region SRUM by DML and Vessel Size of Tuna Purse Seine Fleet in EPO

Conditional Logit Estimates

DML=0 Vessel_Size=4_XLarge (1,250-1,800t)

Parameter Estimates

Parameter	DF	Estimate	Standard Error	t Value	Approx Pr > |t|
LDistant_Expected	1	-2.0588	0.3217	-6.40	<.0001
lCPUE_All_1	1	-0.1731	0.1840	-0.94	0.3468
lRPUE_All_1	1	0.1517	0.1187	1.28	0.2012
LDistant_sinceDep1_z1	0	-1.0737	.	.	.
LDistant_sinceDep1_z2	1	1.3699	0.3961	3.46	0.0005
LDistant_sinceDep1_z3	0	-0.1249	.	.	.
LDistant_sinceDep1_z4	1	-2.6283	0.5227	-5.03	<.0001
LDistant_sinceDep1_z5	1	-2.9921	0.4214	-7.10	<.0001
LDistant_sinceDep1_z6	1	-3.5206	0.8709	-4.04	<.0001
LDistant_sinceDep1_z7	1	-3.1592	0.4894	-6.46	<.0001
LDistant_sinceDep1_z8	1	-3.0585	0.5133	-5.96	<.0001
LDistant_sinceDep1_z9	1	-4.0688	0.5426	-7.50	<.0001
LDistant_sinceDep1_z10	1	-4.1758	0.5462	-7.65	<.0001
LDistant_sinceDep1_z11	1	-1.9424	0.5860	-3.31	0.0009
LDF_travel_sinceDep1_z1	0	0.1872	.	.	.
LDF_travel_sinceDep1_z2	1	0.1471	0.1591	0.92	0.3552
LDF_travel_sinceDep1_z3	0	0.0267	.	.	.
LDF_travel_sinceDep1_z4	1	0.1248	0.1804	0.69	0.4890
LDF_travel_sinceDep1_z5	1	0.1488	0.1748	0.85	0.3947
LDF_travel_sinceDep1_z6	0	-1.8312	.	.	.
LDF_travel_sinceDep1_z7	1	0.0540	0.1414	0.38	0.7027
LDF_travel_sinceDep1_z8	1	0.0949	0.1450	0.65	0.5126
LDF_travel_sinceDep1_z9	1	-0.008928	0.1476	-0.06	0.9518
LDF_travel_sinceDep1_z10	1	-0.0130	0.1488	-0.09	0.9303
LDF_travel_sinceDep1_z11	1	0.002550	0.1505	0.02	0.9865
LDF_search_sinceDep1_z1	0	-0.0366	.	.	.
LDF_search_sinceDep1_z2	1	-0.7615	0.3446	-2.21	0.0271
LDF_search_sinceDep1_z3	0	-0.0254	.	.	.
LDF_search_sinceDep1_z4	1	1.4917	0.4898	3.05	0.0023
LDF_search_sinceDep1_z5	1	1.6459	0.4604	3.57	0.0004

Table A Estimation and Goodness of Fit Result of the First Set Region SRUM by DML and Vessel Size of Tuna Purse Seine Fleet in EPO

Conditional Logit Estimates

DML=0 Vessel_Size=4_XLarge (1,250-1,800t)

Parameter Estimates

Parameter	DF	Estimate	Standard Error	t Value	Approx Pr > |t|
LDF_search_sinceDep1_z6	1	-2.0876	0.9009	-2.32	0.0205
LDF_search_sinceDep1_z7	1	-0.1166	0.3442	-0.34	0.7348
LDF_search_sinceDep1_z8	1	0.1541	0.3759	0.41	0.6818
LDF_search_sinceDep1_z9	1	-0.2868	0.3422	-0.84	0.4020
LDF_search_sinceDep1_z10	1	-0.2446	0.3421	-0.72	0.4745
LDF_search_sinceDep1_z11	1	-0.2258	0.3419	-0.66	0.5089
SST_DOL_L	1	0.001792	0.001678	1.07	0.2854
SST_DOL_H	1	-0.008477	0.003682	-2.30	0.0213
SSH_DOL_L	1	-0.007724	0.002714	-2.85	0.0044
SSH_DOL_H	1	0.006797	0.002376	2.86	0.0042
MLD_DOL_L	1	-0.0116	0.004612	-2.52	0.0116
MLD_DOL_H	1	-0.003629	0.002087	-1.74	0.0820
CHLORO_DOL_L	1	0.009478	0.001870	5.07	<.0001
CHLORO_DOL_H	1	0.000428	0.004893	0.09	0.9304
O2_DOL_L	1	-0.5757	0.0909	-6.33	<.0001
O2_DOL_H	1	-0.0736	0.009369	-7.86	<.0001
Oct_Mar_z1	0	0.0295	.	.	.
Oct_Mar_z2	1	-0.6265	0.6824	-0.92	0.3586
Oct_Mar_z3	0	0.0299	.	.	.
Oct_Mar_z4	1	-0.6657	0.6223	-1.07	0.2848
Oct_Mar_z5	1	-0.4984	0.6152	-0.81	0.4178
Oct_Mar_z6	1	-1.7642	2.6379	-0.67	0.5036
Oct_Mar_z7	1	-1.0565	0.5981	-1.77	0.0773
Oct_Mar_z8	1	-0.4841	0.5722	-0.85	0.3975
Oct_Mar_z9	1	-0.6155	0.6462	-0.95	0.3409
Oct_Mar_z10	1	0.0155	0.6389	0.02	0.9806
Oct_Mar_z11	1	0.6900	0.6125	1.13	0.2599
MEI_z1	0	-0.0134	.	.	.
MEI_z2	1	-0.5954	0.3786	-1.57	0.1158
MEI_z3	0	-0.009286	.	.	.

Table A Estimation and Goodness of Fit Result of the First Set Region SRUM by DML and Vessel Size of Tuna Purse Seine Fleet in EPO

Conditional Logit Estimates

DML=0 Vessel_Size=4_XLarge (1,250-1,800t)

Parameter Estimates

Parameter	DF	Estimate	Standard Error	t Value	Approx Pr > |t|
MEI_z4	1	-0.3503	0.3209	-1.09	0.2750
MEI_z5	1	-0.1007	0.3184	-0.32	0.7519
MEI_z6	1	1.7910	1.8703	0.96	0.3382
MEI_z7	1	-0.3823	0.2970	-1.29	0.1981
MEI_z8	1	-0.3421	0.3113	-1.10	0.2718
MEI_z9	1	0.2090	0.3483	0.60	0.5485
MEI_z10	1	-0.2579	0.3421	-0.75	0.4509
MEI_z11	1	0.3099	0.3094	1.00	0.3166

Table A Estimation and Goodness of Fit Result of the First Set Region SRUM by DML and Vessel Size of Tuna Purse Seine Fleet in EPO

Conditional Logit Estimates

DML=1 Vessel_Size=1_Small (363-700t)

Algorithm converged.

Model Fit Summary	
Dependent Variable	Decision
Number of Observations	539
Number of Cases	6468
Log Likelihood	-447.54721
Log Likelihood Null (LogL(0))	-1339
Maximum Absolute Gradient	2.09979
Number of Iterations	205
Optimization Method	Dual Quasi-Newton
AIC	1031
Schwarz Criterion	1323

Discrete Response Profile

Index	CHOICE	Frequency	Percent
0	1	1	0.19
1	2	1	0.19
2	3	260	48.24
3	4	7	1.30
4	5	3	0.56
5	6	49	9.09
6	7	35	6.49
7	8	38	7.05
8	9	61	11.32
9	10	72	13.36
10	11	10	1.86
11	12	2	0.37

Table A Estimation and Goodness of Fit Result of the First Set Region SRUM by DML and Vessel Size of Tuna Purse Seine Fleet in EPO

Conditional Logit Estimates

DML=1 Vessel_Size=1_Small (363-700t)

Goodness-of-Fit Measures		
Measure	Value	Formula
Likelihood Ratio (R)	1783.6	2 * (LogL - LogL0)
Upper Bound of R (U)	2678.7	- 2 * LogL0
Aldrich-Nelson	0.7679	R / (R+N)
Cragg-Uhler 1	0.9635	1 - exp(-R/N)
Cragg-Uhler 2	0.9702	(1-exp(-R/N)) / (1-exp(-U/N))
Estrella	0.9957	1 - (1-R/U)^(U/N)
Adjusted Estrella	0.9913	1 - ((LogL-K)/LogL0)^(-2/N*LogL0)
McFadden's LRI	0.6659	R / U
Veall-Zimmermann	0.9225	(R * (U+N)) / (U * (R+N))
N = # of observations, K = # of regressors		

Table A Estimation and Goodness of Fit Result of the First Set Region SRUM by DML and Vessel Size of Tuna Purse Seine Fleet in EPO

Conditional Logit Estimates

DML=1 Vessel_Size=1_Small (363-700t)

Parameter Estimates

Parameter	DF	Estimate	Standard Error	t Value	Approx Pr > |t|
LDistant_Expected	1	-4.0531	0.3233	-12.54	<.0001
lCPUE_All_1	1	-0.5671	0.3253	-1.74	0.0813
lRPUE_All_1	1	0.3786	0.2197	1.72	0.0848
LDistant_sinceDep1_z1	1	-0.9368	0.5976	-1.57	0.1170
LDistant_sinceDep1_z2	1	-0.9193	0.7848	-1.17	0.2415
LDistant_sinceDep1_z3	1	-1.9529	0.6818	-2.86	0.0042
LDistant_sinceDep1_z4	1	-1.8461	0.6148	-3.00	0.0027
LDistant_sinceDep1_z5	1	-1.4154	0.6197	-2.28	0.0224
LDistant_sinceDep1_z6	1	-2.0169	0.6962	-2.90	0.0038
LDistant_sinceDep1_z7	1	-1.4409	0.5853	-2.46	0.0138
LDistant_sinceDep1_z8	1	-1.3727	0.6184	-2.22	0.0264
LDistant_sinceDep1_z9	1	-2.0170	0.6455	-3.12	0.0018
LDistant_sinceDep1_z10	1	-2.0916	0.6497	-3.22	0.0013
LDistant_sinceDep1_z11	1	-1.8670	0.6627	-2.82	0.0048
LDF_travel_sinceDep1_z1	1	-0.6267	0.8936	-0.70	0.4830
LDF_travel_sinceDep1_z2	1	-0.5243	1.3445	-0.39	0.6966
LDF_travel_sinceDep1_z3	1	-0.5574	0.8100	-0.69	0.4913
LDF_travel_sinceDep1_z4	1	-0.8147	0.8230	-0.99	0.3222
LDF_travel_sinceDep1_z5	1	-0.4441	0.9431	-0.47	0.6377
LDF_travel_sinceDep1_z6	1	-0.4651	0.8121	-0.57	0.5669
LDF_travel_sinceDep1_z7	1	-0.5555	0.8135	-0.68	0.4947
LDF_travel_sinceDep1_z8	1	-0.5408	0.8100	-0.67	0.5043
LDF_travel_sinceDep1_z9	1	-0.8032	0.8094	-0.99	0.3211
LDF_travel_sinceDep1_z10	1	-0.7534	0.8091	-0.93	0.3518
LDF_travel_sinceDep1_z11	1	-0.8517	0.8139	-1.05	0.2954
LDF_search_sinceDep1_z1	1	-0.1463	0.2384	-0.61	0.5394
LDF_search_sinceDep1_z2	1	1.4245	3.3489	0.43	0.6706
LDF_search_sinceDep1_z3	1	-1.3502	1.8416	-0.73	0.4635
LDF_search_sinceDep1_z4	1	1.2694	1.8438	0.69	0.4912
LDF_search_sinceDep1_z5	1	0.6886	1.9300	0.36	0.7213

Table A Estimation and Goodness of Fit Result of the First Set Region SRUM by DML and Vessel Size of Tuna Purse Seine Fleet in EPO

Conditional Logit Estimates

DML=1 Vessel_Size=1_Small (363-700t)

Parameter Estimates

Parameter	DF	Estimate	Standard Error	t Value	Approx Pr > |t|
LDF_search_sinceDep1_z6	1	-0.9681	1.3863	-0.70	0.4850
LDF_search_sinceDep1_z7	1	-0.008843	1.3151	-0.01	0.9946
LDF_search_sinceDep1_z8	1	-0.6609	1.2275	-0.54	0.5903
LDF_search_sinceDep1_z9	1	-0.3513	1.2121	-0.29	0.7719
LDF_search_sinceDep1_z10	1	-0.2970	1.2427	-0.24	0.8111
LDF_search_sinceDep1_z11	1	1.1061	1.4361	0.77	0.4412
SST_DOL_L	1	0.008899	0.003559	2.50	0.0124
SST_DOL_H	1	-0.0161	0.005942	-2.70	0.0068
SSH_DOL_L	1	-0.006432	0.004414	-1.46	0.1451
SSH_DOL_H	1	0.007791	0.004516	1.73	0.0845
MLD_DOL_L	1	0.0192	0.006106	3.15	0.0016
MLD_DOL_H	1	-0.006169	0.002443	-2.53	0.0116
CHLORO_DOL_L	1	0.004325	0.004506	0.96	0.3371
CHLORO_DOL_H	1	-0.000810	0.006303	-0.13	0.8978
O2_DOL_L	1	0.0441	0.0144	3.06	0.0022
O2_DOL_H	1	-0.0341	0.0102	-3.35	0.0008
Oct_Mar_z1	1	-1.3404	4.4408	-0.30	0.7628
Oct_Mar_z2	1	-2.6618	4.0779	-0.65	0.5139
Oct_Mar_z3	1	-0.1745	1.8339	-0.10	0.9242
Oct_Mar_z4	1	-0.6036	1.9473	-0.31	0.7566
Oct_Mar_z5	1	0.0637	2.2404	0.03	0.9773
Oct_Mar_z6	1	-0.3294	1.8763	-0.18	0.8606
Oct_Mar_z7	1	-0.8435	1.8194	-0.46	0.6429
Oct_Mar_z8	1	0.1748	1.8056	0.10	0.9229
Oct_Mar_z9	1	-0.2927	1.8127	-0.16	0.8717
Oct_Mar_z10	1	0.7320	1.7983	0.41	0.6839
Oct_Mar_z11	1	1.0918	1.8719	0.58	0.5597
MEI_z1	1	-1.8188	1.5237	-1.19	0.2326
MEI_z2	1	-0.7514	2.0550	-0.37	0.7146
MEI_z3	1	-2.1417	0.9075	-2.36	0.0183

Table A Estimation and Goodness of Fit Result of the First Set Region SRUM by DML and Vessel Size of Tuna Purse Seine Fleet in EPO

Conditional Logit Estimates

DML=1 Vessel_Size=1_Small (363-700t)

Parameter Estimates

Parameter	DF	Estimate	Standard Error	t Value	Approx Pr > |t|
MEI_z4	1	-2.1852	1.0062	-2.17	0.0299
MEI_z5	1	-3.5921	1.4323	-2.51	0.0121
MEI_z6	1	-1.9838	0.9075	-2.19	0.0288
MEI_z7	1	-1.9271	0.9171	-2.10	0.0356
MEI_z8	1	-1.6183	0.8992	-1.80	0.0719
MEI_z9	1	-1.7013	0.9002	-1.89	0.0588
MEI_z10	1	-1.4526	0.9035	-1.61	0.1079
MEI_z11	1	-1.1063	0.9423	-1.17	0.2404

Table A Estimation and Goodness of Fit Result of the First Set Region SRUM by DML and Vessel Size of Tuna Purse Seine Fleet in EPO

Conditional Logit Estimates

DML=1 Vessel_Size=2_Median (700-1,050t)

Algorithm converged.

Model Fit Summary	
Dependent Variable	Decision
Number of Observations	1555
Number of Cases	18660
Log Likelihood	-1852
Log Likelihood Null (LogL(0))	-3864
Maximum Absolute Gradient	0.88123
Number of Iterations	210
Optimization Method	Dual Quasi-Newton
AIC	3840
Schwarz Criterion	4203

Discrete Response Profile

Index	CHOICE	Frequency	Percent
0	1	6	0.39
1	2	14	0.90
2	3	277	17.81
3	4	54	3.47
4	5	23	1.48
5	6	102	6.56
6	7	229	14.73
7	8	95	6.11
8	9	514	33.05
9	10	187	12.03
10	11	47	3.02
11	12	7	0.45

Table A Estimation and Goodness of Fit Result of the First Set Region SRUM by DML and Vessel Size of Tuna Purse Seine Fleet in EPO

Conditional Logit Estimates

DML=1 Vessel_Size=2_Median (700-1,050t)

Goodness-of-Fit Measures		
Measure	Value	Formula
Likelihood Ratio (R)	4024.3	2 * (LogL - LogL0)
Upper Bound of R (U)	7728.1	- 2 * LogL0
Aldrich-Nelson	0.7213	R / (R+N)
Cragg-Uhler 1	0.9248	1 - exp(-R/N)
Cragg-Uhler 2	0.9313	(1-exp(-R/N)) / (1-exp(-U/N))
Estrella	0.9741	1 - (1-R/U)^(U/N)
Adjusted Estrella	0.9691	1 - ((LogL-K)/LogL0)^(-2/N*LogL0)
McFadden's LRI	0.5207	R / U
Veall-Zimmermann	0.8664	(R * (U+N)) / (U * (R+N))
N = # of observations, K = # of regressors		

Table A Estimation and Goodness of Fit Result of the First Set Region SRUM by DML and Vessel Size of Tuna Purse Seine Fleet in EPO

Conditional Logit Estimates

DML=1 Vessel_Size=2_Median (700-1,050t)

Parameter Estimates

Parameter	DF	Estimate	Standard Error	t Value	Approx Pr > |t|
LDistant_Expected	1	-3.0947	0.1146	-27.01	<.0001
lCPUE_All_1	1	-0.0447	0.1501	-0.30	0.7658
lRPUE_All_1	1	0.0681	0.1018	0.67	0.5035
LDistant_sinceDep1_z1	1	-0.2856	0.2139	-1.33	0.1819
LDistant_sinceDep1_z2	1	-0.2409	0.1916	-1.26	0.2087
LDistant_sinceDep1_z3	1	-2.0477	0.3044	-6.73	<.0001
LDistant_sinceDep1_z4	1	-1.6485	0.2895	-5.69	<.0001
LDistant_sinceDep1_z5	1	-1.6910	0.2703	-6.26	<.0001
LDistant_sinceDep1_z6	1	-2.3709	0.3324	-7.13	<.0001
LDistant_sinceDep1_z7	1	-1.6553	0.2908	-5.69	<.0001
LDistant_sinceDep1_z8	1	-1.8348	0.3042	-6.03	<.0001
LDistant_sinceDep1_z9	1	-2.3120	0.3199	-7.23	<.0001
LDistant_sinceDep1_z10	1	-2.3058	0.3203	-7.20	<.0001
LDistant_sinceDep1_z11	1	-1.9406	0.3159	-6.14	<.0001
LDF_travel_sinceDep1_z1	1	0.3593	0.3946	0.91	0.3625
LDF_travel_sinceDep1_z2	1	0.8124	0.3027	2.68	0.0073
LDF_travel_sinceDep1_z3	1	0.1901	0.1192	1.59	0.1108
LDF_travel_sinceDep1_z4	1	0.4643	0.1537	3.02	0.0025
LDF_travel_sinceDep1_z5	1	0.2203	0.1507	1.46	0.1437
LDF_travel_sinceDep1_z6	1	0.2523	0.1208	2.09	0.0368
LDF_travel_sinceDep1_z7	1	0.2627	0.1150	2.28	0.0223
LDF_travel_sinceDep1_z8	1	0.2627	0.1184	2.22	0.0265
LDF_travel_sinceDep1_z9	1	0.1711	0.1144	1.50	0.1347
LDF_travel_sinceDep1_z10	1	0.2874	0.1166	2.46	0.0137
LDF_travel_sinceDep1_z11	1	0.4843	0.1501	3.23	0.0013
LDF_search_sinceDep1_z1	1	-0.0556	0.0813	-0.68	0.4939
LDF_search_sinceDep1_z2	1	-0.5985	0.7692	-0.78	0.4365
LDF_search_sinceDep1_z3	1	-0.9912	0.6167	-1.61	0.1080
LDF_search_sinceDep1_z4	1	-0.0422	0.5975	-0.07	0.9437
LDF_search_sinceDep1_z5	1	0.9211	0.6325	1.46	0.1453

Table A Estimation and Goodness of Fit Result of the First Set Region SRUM by DML and Vessel Size of Tuna Purse Seine Fleet in EPO

Conditional Logit Estimates

DML=1 Vessel_Size=2_Median (700-1,050t)

Parameter Estimates

Parameter	DF	Estimate	Standard Error	t Value	Approx Pr > |t|
LDF_search_sinceDep1_z6	1	-0.7321	0.4897	-1.49	0.1350
LDF_search_sinceDep1_z7	1	-0.7102	0.4633	-1.53	0.1253
LDF_search_sinceDep1_z8	1	-0.4514	0.4624	-0.98	0.3289
LDF_search_sinceDep1_z9	1	-0.7499	0.4600	-1.63	0.1030
LDF_search_sinceDep1_z10	1	-0.6755	0.4794	-1.41	0.1588
LDF_search_sinceDep1_z11	1	-0.0704	0.5539	-0.13	0.8989
SST_DOL_L	1	0.008593	0.001565	5.49	<.0001
SST_DOL_H	1	-0.0112	0.002432	-4.62	<.0001
SSH_DOL_L	1	-0.006229	0.002085	-2.99	0.0028
SSH_DOL_H	1	-0.000873	0.001794	-0.49	0.6266
MLD_DOL_L	1	0.008781	0.002783	3.16	0.0016
MLD_DOL_H	1	-0.006259	0.001230	-5.09	<.0001
CHLORO_DOL_L	1	0.000806	0.001804	0.45	0.6552
CHLORO_DOL_H	1	0.003244	0.003060	1.06	0.2891
O2_DOL_L	1	0.0279	0.005151	5.42	<.0001
O2_DOL_H	1	-0.0329	0.005041	-6.53	<.0001
Oct_Mar_z1	1	-4.1608	3.1187	-1.33	0.1822
Oct_Mar_z2	1	-2.7567	1.1611	-2.37	0.0176
Oct_Mar_z3	1	-1.5490	0.9314	-1.66	0.0963
Oct_Mar_z4	1	-1.9469	0.9410	-2.07	0.0386
Oct_Mar_z5	1	-0.5111	0.9842	-0.52	0.6036
Oct_Mar_z6	1	-1.9961	0.9362	-2.13	0.0330
Oct_Mar_z7	1	-1.7004	0.9060	-1.88	0.0605
Oct_Mar_z8	1	-0.8258	0.9145	-0.90	0.3665
Oct_Mar_z9	1	-0.5982	0.9035	-0.66	0.5079
Oct_Mar_z10	1	0.7562	0.9118	0.83	0.4069
Oct_Mar_z11	1	-0.2346	0.9467	-0.25	0.8043
MEI_z1	1	-0.6139	0.6663	-0.92	0.3569
MEI_z2	1	-0.5759	0.5681	-1.01	0.3108
MEI_z3	1	-0.8836	0.3918	-2.26	0.0241

Table A Estimation and Goodness of Fit Result of the First Set Region SRUM by DML and Vessel Size of Tuna Purse Seine Fleet in EPO

Conditional Logit Estimates

DML=1 Vessel_Size=2_Median (700-1,050t)

Parameter Estimates

Parameter	DF	Estimate	Standard Error	t Value	Approx Pr > |t|
MEI_z4	1	-0.7375	0.4047	-1.82	0.0684
MEI_z5	1	-0.8568	0.4274	-2.00	0.0450
MEI_z6	1	-0.8881	0.3913	-2.27	0.0232
MEI_z7	1	-0.5708	0.3791	-1.51	0.1321
MEI_z8	1	-0.9575	0.3898	-2.46	0.0140
MEI_z9	1	-0.8519	0.3767	-2.26	0.0237
MEI_z10	1	-0.9028	0.3818	-2.36	0.0180
MEI_z11	1	-1.4319	0.4045	-3.54	0.0004

Table A Estimation and Goodness of Fit Result of the First Set Region SRUM by DML and Vessel Size of Tuna Purse Seine Fleet in EPO

Conditional Logit Estimates

DML=1 Vessel_Size=3_Large (1,050-1,250t)

Algorithm converged.

Model Fit Summary	
Dependent Variable	Decision
Number of Observations	2240
Number of Cases	26880
Log Likelihood	-2502
Log Likelihood Null (LogL(0))	-5566
Maximum Absolute Gradient	9.42178
Number of Iterations	180
Optimization Method	Dual Quasi-Newton
AIC	5141
Schwarz Criterion	5529

Discrete Response Profile

Index	CHOICE	Frequency	Percent
0	1	22	0.98
1	2	15	0.67
2	3	666	29.73
3	4	64	2.86
4	5	18	0.80
5	6	177	7.90
6	7	278	12.41
7	8	134	5.98
8	9	608	27.14
9	10	185	8.26
10	11	61	2.72
11	12	12	0.54

Table A Estimation and Goodness of Fit Result of the First Set Region SRUM by DML and Vessel Size of Tuna Purse Seine Fleet in EPO

Conditional Logit Estimates

DML=1 Vessel_Size=3_Large (1,050-1,250t)

Goodness-of-Fit Measures		
Measure	Value	Formula
Likelihood Ratio (R)	6127.7	2 * (LogL - LogL0)
Upper Bound of R (U)	11132	- 2 * LogL0
Aldrich-Nelson	0.7323	R / (R+N)
Cragg-Uhler 1	0.9351	1 - exp(-R/N)
Cragg-Uhler 2	0.9417	(1-exp(-R/N)) / (1-exp(-U/N))
Estrella	0.9812	1 - (1-R/U)^(U/N)
Adjusted Estrella	0.9785	1 - ((LogL-K)/LogL0)^(-2/N*LogL0)
McFadden's LRI	0.5504	R / U
Veall-Zimmermann	0.8797	(R * (U+N)) / (U * (R+N))
N = # of observations, K = # of regressors		

Table A Estimation and Goodness of Fit Result of the First Set Region SRUM by DML and Vessel Size of Tuna Purse Seine Fleet in EPO

Conditional Logit Estimates

DML=1 Vessel_Size=3_Large (1,050-1,250t)

Parameter Estimates

Parameter	DF	Estimate	Standard Error	t Value	Approx Pr > |t|
LDistant_Expected	1	-2.8860	0.0857	-33.67	<.0001
lCPUE_All_1	1	-0.0937	0.1266	-0.74	0.4592
lRPUE_All_1	1	0.0894	0.0841	1.06	0.2881
LDistant_sinceDep1_z1	1	0.0116	0.1503	0.08	0.9385
LDistant_sinceDep1_z2	1	-0.1468	0.1945	-0.75	0.4504
LDistant_sinceDep1_z3	1	-1.2776	0.2390	-5.35	<.0001
LDistant_sinceDep1_z4	1	-0.9737	0.2245	-4.34	<.0001
LDistant_sinceDep1_z5	1	-0.9613	0.2229	-4.31	<.0001
LDistant_sinceDep1_z6	1	-1.4289	0.2487	-5.75	<.0001
LDistant_sinceDep1_z7	1	-0.8550	0.2215	-3.86	0.0001
LDistant_sinceDep1_z8	1	-0.9728	0.2309	-4.21	<.0001
LDistant_sinceDep1_z9	1	-1.3573	0.2394	-5.67	<.0001
LDistant_sinceDep1_z10	1	-1.3233	0.2397	-5.52	<.0001
LDistant_sinceDep1_z11	1	-1.2182	0.2420	-5.03	<.0001
LDF_travel_sinceDep1_z1	1	0.3226	0.2372	1.36	0.1739
LDF_travel_sinceDep1_z2	1	0.3015	0.3390	0.89	0.3738
LDF_travel_sinceDep1_z3	1	0.0519	0.1795	0.29	0.7725
LDF_travel_sinceDep1_z4	1	0.3381	0.2143	1.58	0.1147
LDF_travel_sinceDep1_z5	1	0.1129	0.2314	0.49	0.6257
LDF_travel_sinceDep1_z6	1	0.1830	0.1816	1.01	0.3137
LDF_travel_sinceDep1_z7	1	0.0969	0.1782	0.54	0.5867
LDF_travel_sinceDep1_z8	1	0.1191	0.1809	0.66	0.5104
LDF_travel_sinceDep1_z9	1	-0.0104	0.1777	-0.06	0.9532
LDF_travel_sinceDep1_z10	1	0.1029	0.1797	0.57	0.5669
LDF_travel_sinceDep1_z11	1	0.0635	0.1858	0.34	0.7324
LDF_search_sinceDep1_z1	1	0.1554	0.0567	2.74	0.0061
LDF_search_sinceDep1_z2	1	1.6037	0.5643	2.84	0.0045
LDF_search_sinceDep1_z3	1	-0.1163	0.1671	-0.70	0.4864
LDF_search_sinceDep1_z4	1	1.1683	0.3104	3.76	0.0002
LDF_search_sinceDep1_z5	1	1.2844	0.5030	2.55	0.0107

Table A Estimation and Goodness of Fit Result of the First Set Region SRUM by DML and Vessel Size of Tuna Purse Seine Fleet in EPO

Conditional Logit Estimates

DML=1 Vessel_Size=3_Large (1,050-1,250t)

Parameter Estimates					
Parameter	DF	Estimate	Standard Error	t Value	Approx Pr > |t|
LDF_search_sinceDep1_z6	1	-0.5413	0.2913	-1.86	0.0632
LDF_search_sinceDep1_z7	1	-0.6429	0.3392	-1.90	0.0580
LDF_search_sinceDep1_z8	1	-0.2921	0.4094	-0.71	0.4755
LDF_search_sinceDep1_z9	1	-1.0723	0.4437	-2.42	0.0157
LDF_search_sinceDep1_z10	1	-1.1869	0.4982	-2.38	0.0172
LDF_search_sinceDep1_z11	1	-0.1542	0.5904	-0.26	0.7939
SST_DOL_L	1	0.006881	0.001285	5.36	<.0001
SST_DOL_H	1	-0.005288	0.001995	-2.65	0.0080
SSH_DOL_L	1	-0.006634	0.001839	-3.61	0.0003
SSH_DOL_H	1	0.001103	0.001507	0.73	0.4641
MLD_DOL_L	1	0.005704	0.002426	2.35	0.0187
MLD_DOL_H	1	-0.005814	0.000956	-6.08	<.0001
CHLORO_DOL_L	1	0.002721	0.001574	1.73	0.0838
CHLORO_DOL_H	1	0.004571	0.002769	1.65	0.0987
O2_DOL_L	1	0.0272	0.004048	6.73	<.0001
O2_DOL_H	1	-0.0224	0.003589	-6.23	<.0001
Oct_Mar_z1	1	-1.9605	1.0253	-1.91	0.0559
Oct_Mar_z2	1	-2.0867	0.9385	-2.22	0.0262
Oct_Mar_z3	1	-0.6466	0.6673	-0.97	0.3325
Oct_Mar_z4	1	-1.5041	0.6980	-2.16	0.0312
Oct_Mar_z5	1	-0.6866	0.7834	-0.88	0.3808
Oct_Mar_z6	1	-0.7670	0.6728	-1.14	0.2542
Oct_Mar_z7	1	-0.4803	0.6518	-0.74	0.4612
Oct_Mar_z8	1	-0.0351	0.6561	-0.05	0.9574
Oct_Mar_z9	1	0.0279	0.6499	0.04	0.9657
Oct_Mar_z10	1	0.8023	0.6610	1.21	0.2248
Oct_Mar_z11	1	0.9218	0.7102	1.30	0.1943
MEI_z1	1	-0.4125	0.4032	-1.02	0.3064
MEI_z2	1	-0.4493	0.4316	-1.04	0.2978
MEI_z3	1	-0.8524	0.3203	-2.66	0.0078

Table A Estimation and goodness of fit result of the first set region sequential random utility model of the tuna purse seine fleet in eastern Pacific Ocean by DML and vessel size.

Conditional Logit Estimates

DML=1 Vessel_Size=3_Large (1,050-1,250t)

Parameter Estimates

Parameter	DF	Estimate	Standard Error	t Value	Approx Pr > |t|
MEI_z4	1	-1.1325	0.3501	-3.23	0.0012
MEI_z5	1	-0.2132	0.3903	-0.55	0.5849
MEI_z6	1	-0.9413	0.3229	-2.92	0.0035
MEI_z7	1	-0.7560	0.3145	-2.40	0.0162
MEI_z8	1	-0.9723	0.3252	-2.99	0.0028
MEI_z9	1	-0.8862	0.3144	-2.82	0.0048
MEI_z10	1	-0.7872	0.3222	-2.44	0.0146
MEI_z11	1	-1.1335	0.3378	-3.36	0.0008

Table A (cont) Estimation and goodness of fit result of the first set region sequential random utility model of the tuna purse seine fleet in eastern Pacific Ocean by DML and vessel size.

Conditional Logit Estimates

DML=1 Vessel_Size=4_XLarge (1,250-1,800t)

Algorithm converged.

Model Fit Summary	
Dependent Variable	Decision
Number of Observations	640
Number of Cases	7680
Log Likelihood	-893.17361
Log Likelihood Null (LogL(0))	-1590
Maximum Absolute Gradient	5.39963
Number of Iterations	224
Optimization Method	Dual Quasi-Newton
AIC	1922
Schwarz Criterion	2226

Discrete Response Profile

Index	CHOICE	Frequency	Percent
0	1	3	0.47
1	2	11	1.72
2	3	21	3.28
3	4	39	6.09
4	5	19	2.97
5	6	13	2.03
6	7	110	17.19
7	8	63	9.84
8	9	218	34.06
9	10	101	15.78
10	11	33	5.16
11	12	9	1.41

Table A Estimation and Goodness of Fit Result of the First Set Region SRUM by DML and Vessel Size of Tuna Purse Seine Fleet in EPO

Conditional Logit Estimates

DML=1 Vessel_Size=4_XLarge (1,250-1,800t)

Goodness-of-Fit Measures		
Measure	Value	Formula
Likelihood Ratio (R)	1394.3	2 * (LogL - LogL0)
Upper Bound of R (U)	3180.7	- 2 * LogL0
Aldrich-Nelson	0.6854	R / (R+N)
Cragg-Uhler 1	0.8868	1 - exp(-R/N)
Cragg-Uhler 2	0.893	(1-exp(-R/N)) / (1-exp(-U/N))
Estrella	0.9431	1 - (1-R/U)^(U/N)
Adjusted Estrella	0.9181	1 - ((LogL-K)/LogL0)^(-2/N*LogL0)
McFadden's LRI	0.4384	R / U
Veall-Zimmermann	0.8233	(R * (U+N)) / (U * (R+N))
N = # of observations, K = # of regressors		

Table A Estimation and Goodness of Fit Result of the First Set Region SRUM by DML and Vessel Size of Tuna Purse Seine Fleet in EPO

Conditional Logit Estimates

DML=1 Vessel_Size=4_XLarge (1,250-1,800t)

Parameter Estimates

Parameter	DF	Estimate	Standard Error	t Value	Approx Pr > |t|
LDistant_Expected	1	-2.3458	0.1712	-13.70	<.0001
lCPUE_All_1	1	-0.0630	0.2062	-0.31	0.7599
lRPUE_All_1	1	0.0465	0.1339	0.35	0.7283
LDistant_sinceDep1_z1	1	-1.3329	0.7149	-1.86	0.0623
LDistant_sinceDep1_z2	1	-0.2318	0.2164	-1.07	0.2840
LDistant_sinceDep1_z3	1	-1.8672	0.4465	-4.18	<.0001
LDistant_sinceDep1_z4	1	-1.7749	0.4509	-3.94	<.0001
LDistant_sinceDep1_z5	1	-1.5567	0.3944	-3.95	<.0001
LDistant_sinceDep1_z6	1	-2.2859	0.5334	-4.29	<.0001
LDistant_sinceDep1_z7	1	-1.7219	0.4513	-3.82	0.0001
LDistant_sinceDep1_z8	1	-1.8043	0.4711	-3.83	0.0001
LDistant_sinceDep1_z9	1	-2.3341	0.5025	-4.65	<.0001
LDistant_sinceDep1_z10	1	-2.3768	0.5047	-4.71	<.0001
LDistant_sinceDep1_z11	1	-1.8149	0.4843	-3.75	0.0002
LDF_travel_sinceDep1_z1	1	-0.5038	0.4454	-1.13	0.2580
LDF_travel_sinceDep1_z2	1	1.5619	0.4735	3.30	0.0010
LDF_travel_sinceDep1_z3	1	0.0293	0.2519	0.12	0.9075
LDF_travel_sinceDep1_z4	1	0.5819	0.2981	1.95	0.0509
LDF_travel_sinceDep1_z5	1	0.3026	0.3358	0.90	0.3676
LDF_travel_sinceDep1_z6	1	0.1214	0.2556	0.48	0.6348
LDF_travel_sinceDep1_z7	1	0.0447	0.2304	0.19	0.8462
LDF_travel_sinceDep1_z8	1	-0.0416	0.2316	-0.18	0.8576
LDF_travel_sinceDep1_z9	1	-0.0753	0.2309	-0.33	0.7444
LDF_travel_sinceDep1_z10	1	-0.0602	0.2323	-0.26	0.7956
LDF_travel_sinceDep1_z11	1	0.1343	0.2451	0.55	0.5838
LDF_search_sinceDep1_z1	1	-0.4737	0.2256	-2.10	0.0358
LDF_search_sinceDep1_z2	1	-5.3567	2.2596	-2.37	0.0178
LDF_search_sinceDep1_z3	1	-4.5451	2.0214	-2.25	0.0245
LDF_search_sinceDep1_z4	1	-2.6276	1.8103	-1.45	0.1467
LDF_search_sinceDep1_z5	1	-2.0420	1.6116	-1.27	0.2051

Table A Estimation and Goodness of Fit Result of the First Set Region SRUM by DML and Vessel Size of Tuna Purse Seine Fleet in EPO

Conditional Logit Estimates

DML=1 Vessel_Size=4_XLarge (1,250-1,800t)

Parameter Estimates

Parameter	DF	Estimate	Standard Error	t Value	Approx Pr > |t|
LDF_search_sinceDep1_z6	1	-3.1304	1.3574	-2.31	0.0211
LDF_search_sinceDep1_z7	1	-2.5956	1.1317	-2.29	0.0218
LDF_search_sinceDep1_z8	1	-1.9880	0.9162	-2.17	0.0300
LDF_search_sinceDep1_z9	1	-1.6776	0.7031	-2.39	0.0170
LDF_search_sinceDep1_z10	1	-1.1423	0.5043	-2.27	0.0235
LDF_search_sinceDep1_z11	1	-0.6245	0.3536	-1.77	0.0774
SST_DOL_L	1	0.005225	0.002139	2.44	0.0146
SST_DOL_H	1	-0.007824	0.003936	-1.99	0.0468
SSH_DOL_L	1	-0.009510	0.002924	-3.25	0.0011
SSH_DOL_H	1	0.000242	0.002869	0.08	0.9329
MLD_DOL_L	1	0.007131	0.004016	1.78	0.0758
MLD_DOL_H	1	-0.006037	0.002003	-3.01	0.0026
CHLORO_DOL_L	1	0.009129	0.002663	3.43	0.0006
CHLORO_DOL_H	1	0.009234	0.004499	2.05	0.0401
O2_DOL_L	1	-0.0120	0.0138	-0.87	0.3827
O2_DOL_H	1	-0.0403	0.009138	-4.41	<.0001
Oct_Mar_z1	1	-6.5238	5.1445	-1.27	0.2048
Oct_Mar_z2	1	-2.8566	1.2920	-2.21	0.0270
Oct_Mar_z3	1	-1.1679	1.0902	-1.07	0.2840
Oct_Mar_z4	1	-1.5835	0.9589	-1.65	0.0987
Oct_Mar_z5	1	-1.7595	0.9993	-1.76	0.0783
Oct_Mar_z6	1	-4.2053	1.3883	-3.03	0.0025
Oct_Mar_z7	1	-1.8756	0.9240	-2.03	0.0424
Oct_Mar_z8	1	-1.5036	0.9160	-1.64	0.1007
Oct_Mar_z9	1	-1.1483	0.9044	-1.27	0.2042
Oct_Mar_z10	1	-0.0375	0.9147	-0.04	0.9673
Oct_Mar_z11	1	-1.2111	0.9482	-1.28	0.2015
MEI_z1	1	2.2629	2.1240	1.07	0.2867
MEI_z2	1	-3.2973	1.0817	-3.05	0.0023
MEI_z3	1	-0.5610	0.4660	-1.20	0.2286

Table A Estimation and Goodness of Fit Result of the First Set Region SRUM by DML and Vessel Size of Tuna Purse Seine Fleet in EPO

Conditional Logit Estimates

DML=1 Vessel_Size=4_XLarge (1,250-1,800t)

Parameter Estimates

Parameter	DF	Estimate	Standard Error	t Value	Approx Pr > |t|
MEI_z4	1	-0.9059	0.3951	-2.29	0.0219
MEI_z5	1	-0.8183	0.4373	-1.87	0.0613
MEI_z6	1	0.5284	0.5379	0.98	0.3259
MEI_z7	1	-0.3705	0.3583	-1.03	0.3011
MEI_z8	1	-0.4332	0.3768	-1.15	0.2502
MEI_z9	1	-0.1755	0.3528	-0.50	0.6189
MEI_z10	1	-0.2448	0.3636	-0.67	0.5008
MEI_z11	1	-0.5834	0.3883	-1.50	0.1330
