# Supplementary material for: Modeling the Spatial Dynamics of International Tuna Fleets
Source: PLoS One. 2016 Aug 18;11(8):e0159626. doi: 10.1371/journal.pone.0159626 (PMC4990267; doi:10.1371/journal.pone.0159626)
Supplement: S2 Appendix — Table B. Estimation and goodness of fit result for the switching region choice model of the tuna purse seine fleet in eastern Pacific Ocean by DML and vessel size. (PDF) [file pone.0159626.s002.pdf]

Table B Estimation Result of the SRUM for Switching Region Sets by DML and Vessel Size of Tuna Purse Seine Fleet in EPO

The MDC Procedure

Conditional Logit Estimates

DML=0 Vessel_Size=1_Small (363-700t)

Algorithm converged.

Model Fit Summary	
Dependent Variable	Decision
Number of Observations	4755
Number of Cases	57060
Log Likelihood	-8368
Log Likelihood Null (LogL(0))	-11816
Maximum Absolute Gradient	2.51529
Number of Iterations	501
Optimization Method	Dual Quasi-Newton
AIC	17094
Schwarz Criterion	18252

Discrete Response Profile			
Index	CHOICE	Frequency	Percent
0	1	3	0.06
1	2	57	1.20
2	3	9	0.19
3	4	326	6.86
4	5	308	6.48
5	6	21	0.44
6	7	930	19.56
7	8	1023	21.51
8	9	545	11.46
9	10	928	19.52
10	11	415	8.73
11	12	190	4.00

Table B Estimation Result of the SRUM for Switching Region Sets by DML and Vessel Size of Tuna Purse Seine Fleet in EPO

The MDC Procedure

Conditional Logit Estimates

DML=0 Vessel_Size=1_Small (363-700t)

Goodness-of-Fit Measures		
Measure	Value	Formula
Likelihood Ratio (R)	6895.1	2 * (LogL - LogL0)
Upper Bound of R (U)	23631	- 2 * LogL0
Aldrich-Nelson	0.5918	R / (R+N)
Cragg-Uhler 1	0.7654	1 - exp(-R/N)
Cragg-Uhler 2	0.7708	(1-exp(-R/N)) / (1-exp(-U/N))
Estrella	0.82	1 - (1-R/U)^(U/N)
Adjusted Estrella	0.8	1 - ((LogL-K)/LogL0)^(-2/N*LogL0)
McFadden's LRI	0.2918	R / U
Veall-Zimmermann	0.7109	(R * (U+N)) / (U * (R+N))
N = # of observations, K = # of regressors		

Table B Estimation Result of the SRUM for Switching Region Sets by DML and Vessel Size of Tuna Purse Seine Fleet in EPO

The MDC Procedure

Conditional Logit Estimates

DML=0 Vessel_Size=1_Small (363-700t)

Parameter Estimates

Parameter	DF	Estimate	Standard Error	t Value	Approx Pr > |t|
LDistant_Expected	1	-0.2578	0.0288	-8.95	<.0001
LDistant_Arrive	1	-0.9472	0.1986	-4.77	<.0001
lCPUE_All_1	1	0.2048	0.0670	3.06	0.0022
lRPUE_All_1	1	-0.1032	0.0425	-2.43	0.0151
LDistant_sinceDep1_z1	1	1.4622	7.4878	0.20	0.8452
LDistant_sinceDep1_z2	1	5.2877	0.7048	7.50	<.0001
LDistant_sinceDep1_z3	1	5.2122	1.1777	4.43	<.0001
LDistant_sinceDep1_z4	1	1.5091	0.3726	4.05	<.0001
LDistant_sinceDep1_z5	1	2.6682	0.4079	6.54	<.0001
LDistant_sinceDep1_z6	1	0.9801	0.8876	1.10	0.2695
LDistant_sinceDep1_z7	1	0.0499	0.3482	0.14	0.8860
LDistant_sinceDep1_z8	1	-0.2761	0.3450	-0.80	0.4235
LDistant_sinceDep1_z9	1	-2.1384	0.3584	-5.97	<.0001
LDistant_sinceDep1_z10	1	-2.0468	0.3527	-5.80	<.0001
LDistant_sinceDep1_z11	1	-1.5300	0.3851	-3.97	<.0001
LDF_search_sinceDep1_z1	1	0.3872	0.5224	0.74	0.4586
LDF_search_sinceDep1_z2	1	-0.0938	5.2419	-0.02	0.9857
LDF_search_sinceDep1_z3	1	-0.1695	4.7548	-0.04	0.9716
LDF_search_sinceDep1_z4	1	1.9078	4.1765	0.46	0.6478
LDF_search_sinceDep1_z5	1	0.6749	3.6584	0.18	0.8536
LDF_search_sinceDep1_z6	1	2.4465	3.2504	0.75	0.4517
LDF_search_sinceDep1_z7	1	1.9356	2.6173	0.74	0.4596
LDF_search_sinceDep1_z8	1	1.8546	2.0996	0.88	0.3771
LDF_search_sinceDep1_z9	1	3.6161	1.5914	2.27	0.0231
LDF_search_sinceDep1_z10	1	3.0515	1.0835	2.82	0.0049
LDF_search_sinceDep1_z11	1	1.6767	0.6239	2.69	0.0072
LDF_travel_sinceDep1_z1	1	5.3314	7.6188	0.70	0.4841
LDF_travel_sinceDep1_z2	1	-0.4444	0.2038	-2.18	0.0292
LDF_travel_sinceDep1_z3	1	1.3369	0.5634	2.37	0.0177

Table B Estimation Result of the SRUM for Switching Region Sets by DML and Vessel Size of Tuna Purse Seine Fleet in EPO

The MDC Procedure

Conditional Logit Estimates

DML=0 Vessel_Size=1_Small (363-700t)

Parameter Estimates

Parameter	DF	Estimate	Standard Error	t Value	Approx Pr > |t|
LDF_travel_sinceDep1_z4	1	-0.2638	0.1216	-2.17	0.0301
LDF_travel_sinceDep1_z5	1	-0.2410	0.1299	-1.85	0.0636
LDF_travel_sinceDep1_z6	1	-0.1872	0.2921	-0.64	0.5217
LDF_travel_sinceDep1_z7	1	-0.2105	0.1090	-1.93	0.0535
LDF_travel_sinceDep1_z8	1	-0.1294	0.1089	-1.19	0.2347
LDF_travel_sinceDep1_z9	1	-0.1116	0.1170	-0.95	0.3399
LDF_travel_sinceDep1_z10	1	0.0233	0.1142	0.20	0.8384
LDF_travel_sinceDep1_z11	1	-0.1528	0.1147	-1.33	0.1828
lSKJ_sinceDep1_z1	1	-2.9102	9.5648	-0.30	0.7609
lSKJ_sinceDep1_z2	1	0.0150	0.0715	0.21	0.8342
lSKJ_sinceDep1_z3	1	0.0914	0.1129	0.81	0.4179
lSKJ_sinceDep1_z4	1	0.004114	0.0301	0.14	0.8913
lSKJ_sinceDep1_z5	1	0.0226	0.0308	0.73	0.4638
lSKJ_sinceDep1_z6	1	-0.1419	0.0754	-1.88	0.0599
lSKJ_sinceDep1_z7	1	0.0494	0.0253	1.95	0.0512
lSKJ_sinceDep1_z8	1	0.0306	0.0249	1.23	0.2188
lSKJ_sinceDep1_z9	1	0.003093	0.0273	0.11	0.9098
lSKJ_sinceDep1_z10	1	0.0326	0.0261	1.25	0.2127
lSKJ_sinceDep1_z11	1	0.0322	0.0281	1.15	0.2517
lYFT_sinceDep1_z1	1	-2.2725	7.1956	-0.32	0.7521
lYFT_sinceDep1_z2	1	-0.0511	0.0505	-1.01	0.3122
lYFT_sinceDep1_z3	1	-0.1500	0.1286	-1.17	0.2434
lYFT_sinceDep1_z4	1	-0.0181	0.0275	-0.66	0.5097
lYFT_sinceDep1_z5	1	-0.0488	0.0275	-1.77	0.0760
lYFT_sinceDep1_z6	1	0.2293	0.1349	1.70	0.0891
lYFT_sinceDep1_z7	1	-0.0262	0.0236	-1.11	0.2687
lYFT_sinceDep1_z8	1	-0.0403	0.0235	-1.71	0.0867
lYFT_sinceDep1_z9	1	0.0187	0.0259	0.72	0.4712
lYFT_sinceDep1_z10	1	-0.0164	0.0243	-0.68	0.4979

Table B Estimation Result of the SRUM for Switching Region Sets by DML and Vessel Size of Tuna Purse Seine Fleet in EPO

The MDC Procedure

Conditional Logit Estimates

DML=0 Vessel_Size=1_Small (363-700t)

Parameter Estimates

Parameter	DF	Estimate	Standard Error	t Value	Approx Pr > |t|
lYFT_sinceDep1_z11	1	-0.0121	0.0260	-0.46	0.6429
lBET_sinceDep1_z1	1	-0.8379	114.3634	-0.01	0.9942
lBET_sinceDep1_z2	1	0.1401	0.0559	2.51	0.0122
lBET_sinceDep1_z3	1	-0.1660	0.1107	-1.50	0.1336
lBET_sinceDep1_z4	1	0.0397	0.0232	1.71	0.0866
lBET_sinceDep1_z5	1	0.0605	0.0238	2.54	0.0110
lBET_sinceDep1_z6	1	-0.0963	0.0552	-1.74	0.0813
lBET_sinceDep1_z7	1	0.007467	0.0193	0.39	0.6987
lBET_sinceDep1_z8	1	0.006984	0.0191	0.36	0.7152
lBET_sinceDep1_z9	1	-0.0231	0.0204	-1.13	0.2588
lBET_sinceDep1_z10	1	-0.0291	0.0194	-1.49	0.1352
lBET_sinceDep1_z11	1	-0.0674	0.0222	-3.04	0.0024
LDF_search_LastRegion_z1	1	0.1025	3.8574	0.03	0.9788
LDF_search_LastRegion_z2	1	0.0359	0.0881	0.41	0.6839
LDF_search_LastRegion_z3	1	-0.1548	0.1914	-0.81	0.4187
LDF_search_LastRegion_z4	1	0.0540	0.0456	1.18	0.2368
LDF_search_LastRegion_z5	1	0.0184	0.0458	0.40	0.6880
LDF_search_LastRegion_z6	1	-0.3557	0.1081	-3.29	0.0010
LDF_search_LastRegion_z7	1	0.0848	0.0373	2.28	0.0228
LDF_search_LastRegion_z8	1	0.0589	0.0367	1.60	0.1087
LDF_search_LastRegion_z9	1	0.0528	0.0408	1.29	0.1959
LDF_search_LastRegion_z10	1	0.0995	0.0391	2.54	0.0109
LDF_search_LastRegion_z11	1	0.1345	0.0442	3.04	0.0023
LDF_travel_LastRegion_z1	1	0.7140	3.1137	0.23	0.8186
LDF_travel_LastRegion_z2	1	0.005012	0.0840	0.06	0.9524
LDF_travel_LastRegion_z3	1	-0.0349	0.1783	-0.20	0.8446
LDF_travel_LastRegion_z4	1	-0.0128	0.0464	-0.28	0.7828
LDF_travel_LastRegion_z5	1	0.0193	0.0482	0.40	0.6891
LDF_travel_LastRegion_z6	1	-0.0814	0.1304	-0.62	0.5325

Table B Estimation Result of the SRUM for Switching Region Sets by DML and Vessel Size of Tuna Purse Seine Fleet in EPO

The MDC Procedure

Conditional Logit Estimates

DML=0 Vessel_Size=1_Small (363-700t)

Parameter Estimates

Parameter	DF	Estimate	Standard Error	t Value	Approx Pr > |t|
LDF_travel_LastRegion_z7	1	-0.0738	0.0392	-1.88	0.0597
LDF_travel_LastRegion_z8	1	-0.0824	0.0389	-2.12	0.0343
LDF_travel_LastRegion_z9	1	-0.008423	0.0423	-0.20	0.8422
LDF_travel_LastRegion_z10	1	-0.0525	0.0400	-1.31	0.1891
LDF_travel_LastRegion_z11	1	-0.0641	0.0428	-1.50	0.1339
lDOL_LastRegion_z1	1	0.2414	156.1916	0.00	0.9988
lDOL_LastRegion_z2	1	7.1328	0.8254	8.64	<.0001
lDOL_LastRegion_z3	1	13.0434	1.3983	9.33	<.0001
lDOL_LastRegion_z4	1	13.4576	1.3613	9.89	<.0001
lDOL_LastRegion_z5	1	13.2417	1.2226	10.83	<.0001
lDOL_LastRegion_z6	1	13.7042	1.3736	9.98	<.0001
lDOL_LastRegion_z7	1	13.0193	1.3113	9.93	<.0001
lDOL_LastRegion_z8	1	13.0786	1.3537	9.66	<.0001
lDOL_LastRegion_z9	1	13.0863	1.3850	9.45	<.0001
lDOL_LastRegion_z10	1	13.2624	1.3821	9.60	<.0001
lDOL_LastRegion_z11	1	11.7487	1.4273	8.23	<.0001
lOBJ_LastRegion_z1	1	-0.1597	0.6384	-0.25	0.8025
lOBJ_LastRegion_z2	1	-0.0715	0.0881	-0.81	0.4172
lOBJ_LastRegion_z3	1	-0.2001	0.2312	-0.87	0.3867
lOBJ_LastRegion_z4	1	0.0161	0.0384	0.42	0.6762
lOBJ_LastRegion_z5	1	0.001269	0.0394	0.03	0.9743
lOBJ_LastRegion_z6	1	0.1888	0.0986	1.91	0.0556
lOBJ_LastRegion_z7	1	-0.005264	0.0329	-0.16	0.8730
lOBJ_LastRegion_z8	1	0.003181	0.0326	0.10	0.9223
lOBJ_LastRegion_z9	1	-0.0183	0.0339	-0.54	0.5890
lOBJ_LastRegion_z10	1	0.0407	0.0324	1.25	0.2098
lOBJ_LastRegion_z11	1	-0.0647	0.0350	-1.85	0.0646
lNOA_LastRegion_z1	1	-0.3638	0.7209	-0.50	0.6138
lNOA_LastRegion_z2	1	-0.0759	0.0851	-0.89	0.3721

Table B Estimation Result of the SRUM for Switching Region Sets by DML and Vessel Size of Tuna Purse Seine Fleet in EPO

The MDC Procedure

Conditional Logit Estimates

DML=0 Vessel_Size=1_Small (363-700t)

Parameter Estimates

Parameter	DF	Estimate	Standard Error	t Value	Approx Pr > |t|
lNOA_LastRegion_z3	1	-0.1754	0.2250	-0.78	0.4356
lNOA_LastRegion_z4	1	0.0109	0.0336	0.33	0.7445
lNOA_LastRegion_z5	1	0.009202	0.0354	0.26	0.7950
lNOA_LastRegion_z6	1	0.3513	0.0964	3.65	0.0003
lNOA_LastRegion_z7	1	0.0698	0.0290	2.41	0.0160
lNOA_LastRegion_z8	1	0.0653	0.0287	2.27	0.0230
lNOA_LastRegion_z9	1	0.1033	0.0296	3.50	0.0005
lNOA_LastRegion_z10	1	0.0998	0.0287	3.48	0.0005
lNOA_LastRegion_z11	1	0.1021	0.0312	3.27	0.0011
lYFT_LastRegion_z1	1	2.6587	7.0742	0.38	0.7070
lYFT_LastRegion_z2	1	-0.0259	0.0538	-0.48	0.6306
lYFT_LastRegion_z3	1	0.4332	0.2332	1.86	0.0632
lYFT_LastRegion_z4	1	-0.0571	0.0300	-1.91	0.0564
lYFT_LastRegion_z5	1	-0.0419	0.0308	-1.36	0.1741
lYFT_LastRegion_z6	1	-0.0483	0.0864	-0.56	0.5762
lYFT_LastRegion_z7	1	-0.0638	0.0267	-2.39	0.0168
lYFT_LastRegion_z8	1	-0.0266	0.0267	-1.00	0.3182
lYFT_LastRegion_z9	1	-0.0133	0.0280	-0.47	0.6363
lYFT_LastRegion_z10	1	-0.0449	0.0268	-1.67	0.0941
lYFT_LastRegion_z11	1	-0.0107	0.0294	-0.36	0.7154
lBET_LastRegion_z1	1	-0.5142	194.3629	-0.00	0.9979
lBET_LastRegion_z2	1	0.0286	0.0567	0.50	0.6137
lBET_LastRegion_z3	1	-0.0752	0.1403	-0.54	0.5919
lBET_LastRegion_z4	1	-0.0334	0.0284	-1.18	0.2392
lBET_LastRegion_z5	1	-0.0328	0.0291	-1.13	0.2598
lBET_LastRegion_z6	1	-0.1037	0.1098	-0.94	0.3449
lBET_LastRegion_z7	1	-0.0358	0.0252	-1.42	0.1548
lBET_LastRegion_z8	1	-0.0503	0.0249	-2.02	0.0435
lBET_LastRegion_z9	1	-0.0339	0.0269	-1.26	0.2071

Table B Estimation Result of the SRUM for Switching Region Sets by DML and Vessel Size of Tuna Purse Seine Fleet in EPO

The MDC Procedure

Conditional Logit Estimates

DML=0 Vessel_Size=1_Small (363-700t)

Parameter Estimates

Parameter	DF	Estimate	Standard Error	t Value	Approx Pr > |t|
lBET_LastRegion_z10	1	-0.0939	0.0256	-3.67	0.0002
lBET_LastRegion_z11	1	-0.0877	0.0312	-2.81	0.0050
lSKJ_LastRegion_z1	1	2.9490	9.8534	0.30	0.7647
lSKJ_LastRegion_z2	1	0.1028	0.0674	1.52	0.1275
lSKJ_LastRegion_z3	1	-0.002806	0.1183	-0.02	0.9811
lSKJ_LastRegion_z4	1	0.0896	0.0309	2.90	0.0037
lSKJ_LastRegion_z5	1	0.0619	0.0316	1.96	0.0500
lSKJ_LastRegion_z6	1	0.000296	0.0763	0.00	0.9969
lSKJ_LastRegion_z7	1	0.0723	0.0260	2.78	0.0055
lSKJ_LastRegion_z8	1	0.0635	0.0258	2.46	0.0138
lSKJ_LastRegion_z9	1	0.0232	0.0269	0.86	0.3874
lSKJ_LastRegion_z10	1	0.0430	0.0258	1.67	0.0956
lSKJ_LastRegion_z11	1	0.0669	0.0286	2.34	0.0191
SST_DOL_L	1	0.003322	0.000606	5.49	<.0001
SST_DOL_H	1	-0.007402	0.001276	-5.80	<.0001
O2_DOL_L	1	-0.4582	0.0812	-5.64	<.0001
O2_DOL_H	1	-0.2516	0.0239	-10.53	<.0001
SSH_DOL_L	1	-0.004543	0.000956	-4.75	<.0001
SSH_DOL_H	1	0.002020	0.000929	2.18	0.0296
MLD_DOL_L	1	-0.003093	0.001527	-2.02	0.0429
MLD_DOL_H	1	-0.003400	0.000700	-4.86	<.0001
CHLORO_DOL_L	1	0.005132	0.000784	6.54	<.0001
CHLORO_DOL_H	1	-0.002702	0.001655	-1.63	0.1026
MEI_z1	1	-0.3677	2.6234	-0.14	0.8885
MEI_z2	1	-0.1586	0.1838	-0.86	0.3881
MEI_z3	1	-0.0649	0.4969	-0.13	0.8962
MEI_z4	1	-0.3196	0.1035	-3.09	0.0020
MEI_z5	1	-0.1990	0.1074	-1.85	0.0639
MEI_z6	1	0.7376	0.2677	2.76	0.0059

Table B Estimation Result of the SRUM for Switching Region Sets by DML and Vessel Size of Tuna Purse Seine Fleet in EPO

The MDC Procedure

Conditional Logit Estimates

DML=0 Vessel_Size=1_Small (363-700t)

Parameter Estimates

Parameter	DF	Estimate	Standard Error	t Value	Approx Pr > |t|
MEI_z7	1	-0.1369	0.0906	-1.51	0.1309
MEI_z8	1	-0.1519	0.0949	-1.60	0.1095
MEI_z9	1	0.1451	0.0976	1.49	0.1370
MEI_z10	1	0.0668	0.0935	0.71	0.4749
MEI_z11	1	0.3354	0.1037	3.23	0.0012

Table B Estimation Result of the SRUM for Switching Region Sets by DML and Vessel Size of Tuna Purse Seine Fleet in EPO

The MDC Procedure

Conditional Logit Estimates

DML=0 Vessel_Size=2_Median (700-1,050t)

Algorithm converged.

Model Fit Summary	
Dependent Variable	Decision
Number of Observations	3733
Number of Cases	44796
Log Likelihood	-6904
Log Likelihood Null (LogL(0))	-9276
Maximum Absolute Gradient	1.11593
Number of Iterations	458
Optimization Method	Dual Quasi-Newton
AIC	14166
Schwarz Criterion	15281

Discrete Response Profile

Index	CHOICE	Frequency	Percent
0	1	1	0.03
1	2	129	3.46
2	3	4	0.11
3	4	337	9.03
4	5	574	15.38
5	6	1	0.03
6	7	617	16.53
7	8	850	22.77
8	9	177	4.74
9	10	418	11.20
10	11	225	6.03
11	12	400	10.72

Table B Estimation Result of the SRUM for Switching Region Sets by DML and Vessel Size of Tuna Purse Seine Fleet in EPO

The MDC Procedure

Conditional Logit Estimates

DML=0 Vessel_Size=2_Median (700-1,050t)

Goodness-of-Fit Measures		
Measure	Value	Formula
Likelihood Ratio (R)	4744.1	2 * (LogL - LogL0)
Upper Bound of R (U)	18552	- 2 * LogL0
Aldrich-Nelson	0.5596	R / (R+N)
Cragg-Uhler 1	0.7194	1 - exp(-R/N)
Cragg-Uhler 2	0.7244	(1-exp(-R/N)) / (1-exp(-U/N))
Estrella	0.7696	1 - (1-R/U)^(U/N)
Adjusted Estrella	0.7383	1 - ((LogL-K)/LogL0)^(-2/N*LogL0)
McFadden's LRI	0.2557	R / U
Veall-Zimmermann	0.6722	(R * (U+N)) / (U * (R+N))
N = # of observations, K = # of regressors		

Table B Estimation Result of the SRUM for Switching Region Sets by DML and Vessel Size of Tuna Purse Seine Fleet in EPO

The MDC Procedure

Conditional Logit Estimates

DML=0 Vessel_Size=2_Median (700-1,050t)

Parameter Estimates

Parameter	DF	Estimate	Standard Error	t Value	Approx Pr > |t|
LDistant_Expected	1	-0.4280	0.0319	-13.42	<.0001
LDistant_Arrive	1	-0.5853	0.3355	-1.74	0.0810
lCPUE_All_1	1	-0.0113	0.0687	-0.16	0.8692
lRPUE_All_1	1	0.0237	0.0433	0.55	0.5842
LDistant_sinceDep1_z1	1	-1.2578	6.6152	-0.19	0.8492
LDistant_sinceDep1_z2	1	4.8050	0.5678	8.46	<.0001
LDistant_sinceDep1_z3	1	-2.8171	3.1764	-0.89	0.3751
LDistant_sinceDep1_z4	1	1.3262	0.3588	3.70	0.0002
LDistant_sinceDep1_z5	1	1.8334	0.3287	5.58	<.0001
LDistant_sinceDep1_z6	1	-5.8944	6.5082	-0.91	0.3651
LDistant_sinceDep1_z7	1	-0.3483	0.2996	-1.16	0.2450
LDistant_sinceDep1_z8	1	-0.4596	0.2831	-1.62	0.1045
LDistant_sinceDep1_z9	1	-2.5006	0.3825	-6.54	<.0001
LDistant_sinceDep1_z10	1	-2.3562	0.3329	-7.08	<.0001
LDistant_sinceDep1_z11	1	-1.5559	0.3857	-4.03	<.0001
LDF_search_sinceDep1_z1	1	0.1555	0.4447	0.35	0.7266
LDF_search_sinceDep1_z2	1	-2.1458	4.4595	-0.48	0.6304
LDF_search_sinceDep1_z3	1	3.9034	4.7477	0.82	0.4110
LDF_search_sinceDep1_z4	1	0.1215	3.5618	0.03	0.9728
LDF_search_sinceDep1_z5	1	-0.6296	3.1155	-0.20	0.8399
LDF_search_sinceDep1_z6	1	0.1259	9.6023	0.01	0.9895
LDF_search_sinceDep1_z7	1	1.0867	2.2298	0.49	0.6260
LDF_search_sinceDep1_z8	1	1.2302	1.7873	0.69	0.4913
LDF_search_sinceDep1_z9	1	3.0415	1.3761	2.21	0.0271
LDF_search_sinceDep1_z10	1	3.0464	0.9336	3.26	0.0011
LDF_search_sinceDep1_z11	1	1.8179	0.5586	3.25	0.0011
LDF_travel_sinceDep1_z1	1	5.0599	3.6040	1.40	0.1603
LDF_travel_sinceDep1_z2	1	0.0790	0.1804	0.44	0.6615
LDF_travel_sinceDep1_z3	1	-0.7480	1.8876	-0.40	0.6919

Table B Estimation Result of the SRUM for Switching Region Sets by DML and Vessel Size of Tuna Purse Seine Fleet in EPO

The MDC Procedure

Conditional Logit Estimates

DML=0 Vessel_Size=2_Median (700-1,050t)

Parameter Estimates

Parameter	DF	Estimate	Standard Error	t Value	Approx Pr > |t|
LDF_travel_sinceDep1_z4	1	-0.0683	0.1076	-0.64	0.5254
LDF_travel_sinceDep1_z5	1	0.006559	0.0965	0.07	0.9458
LDF_travel_sinceDep1_z6	1	1.8217	2.4858	0.73	0.4637
LDF_travel_sinceDep1_z7	1	0.0815	0.0914	0.89	0.3722
LDF_travel_sinceDep1_z8	1	0.0914	0.0862	1.06	0.2885
LDF_travel_sinceDep1_z9	1	0.2617	0.1394	1.88	0.0605
LDF_travel_sinceDep1_z10	1	0.1968	0.1121	1.76	0.0791
LDF_travel_sinceDep1_z11	1	0.0585	0.1044	0.56	0.5755
lSKJ_sinceDep1_z1	1	0.4336	1.6078	0.27	0.7874
lSKJ_sinceDep1_z2	1	0.0142	0.0502	0.28	0.7781
lSKJ_sinceDep1_z3	1	-0.1298	0.1756	-0.74	0.4599
lSKJ_sinceDep1_z4	1	0.0558	0.0291	1.92	0.0552
lSKJ_sinceDep1_z5	1	0.0521	0.0261	2.00	0.0460
lSKJ_sinceDep1_z6	1	-2.4896	4.4555	-0.56	0.5763
lSKJ_sinceDep1_z7	1	0.0659	0.0244	2.70	0.0068
lSKJ_sinceDep1_z8	1	0.0542	0.0231	2.35	0.0188
lSKJ_sinceDep1_z9	1	-0.007186	0.0346	-0.21	0.8356
lSKJ_sinceDep1_z10	1	0.0790	0.0315	2.51	0.0122
lSKJ_sinceDep1_z11	1	0.0515	0.0307	1.68	0.0933
lYFT_sinceDep1_z1	1	0.1903	0.8074	0.24	0.8137
lYFT_sinceDep1_z2	1	-0.0857	0.0377	-2.28	0.0228
lYFT_sinceDep1_z3	1	-0.0739	0.1492	-0.50	0.6202
lYFT_sinceDep1_z4	1	-0.0586	0.0250	-2.34	0.0191
lYFT_sinceDep1_z5	1	-0.0588	0.0224	-2.63	0.0086
lYFT_sinceDep1_z6	1	-1.9636	7.5442	-0.26	0.7946
lYFT_sinceDep1_z7	1	-0.0714	0.0211	-3.38	0.0007
lYFT_sinceDep1_z8	1	-0.0735	0.0206	-3.57	0.0004
lYFT_sinceDep1_z9	1	-0.0169	0.0315	-0.54	0.5923
lYFT_sinceDep1_z10	1	-0.0260	0.0243	-1.07	0.2836

Table B Estimation Result of the SRUM for Switching Region Sets by DML and Vessel Size of Tuna Purse Seine Fleet in EPO

The MDC Procedure

Conditional Logit Estimates

DML=0 Vessel_Size=2_Median (700-1,050t)

Parameter Estimates

Parameter	DF	Estimate	Standard Error	t Value	Approx Pr > |t|
lYFT_sinceDep1_z11	1	-0.0467	0.0267	-1.75	0.0805
lBET_sinceDep1_z1	1	0.3983	1.1716	0.34	0.7339
lBET_sinceDep1_z2	1	0.0760	0.0421	1.80	0.0713
lBET_sinceDep1_z3	1	0.1419	0.2174	0.65	0.5141
lBET_sinceDep1_z4	1	0.003845	0.0213	0.18	0.8570
lBET_sinceDep1_z5	1	0.0198	0.0196	1.01	0.3105
lBET_sinceDep1_z6	1	-0.5408	8.6215	-0.06	0.9500
lBET_sinceDep1_z7	1	-0.0131	0.0176	-0.75	0.4558
lBET_sinceDep1_z8	1	-0.0153	0.0167	-0.92	0.3591
lBET_sinceDep1_z9	1	-0.000546	0.0230	-0.02	0.9811
lBET_sinceDep1_z10	1	-0.0463	0.0185	-2.50	0.0125
lBET_sinceDep1_z11	1	-0.0766	0.0216	-3.55	0.0004
LDF_search_LastRegion_z1	1	0.4519	0.7171	0.63	0.5286
LDF_search_LastRegion_z2	1	-0.0433	0.0581	-0.75	0.4561
LDF_search_LastRegion_z3	1	-0.0681	0.3030	-0.22	0.8222
LDF_search_LastRegion_z4	1	0.0987	0.0423	2.34	0.0195
LDF_search_LastRegion_z5	1	0.0314	0.0311	1.01	0.3127
LDF_search_LastRegion_z6	1	3.9665	7.2633	0.55	0.5850
LDF_search_LastRegion_z7	1	0.0339	0.0301	1.12	0.2612
LDF_search_LastRegion_z8	1	0.0567	0.0284	1.99	0.0462
LDF_search_LastRegion_z9	1	0.0676	0.0452	1.49	0.1351
LDF_search_LastRegion_z10	1	0.0633	0.0371	1.71	0.0875
LDF_search_LastRegion_z11	1	0.0790	0.0378	2.09	0.0363
LDF_travel_LastRegion_z1	1	-0.1620	0.3982	-0.41	0.6841
LDF_travel_LastRegion_z2	1	0.0545	0.0622	0.88	0.3812
LDF_travel_LastRegion_z3	1	2.1482	1.6237	1.32	0.1858
LDF_travel_LastRegion_z4	1	0.0345	0.0374	0.92	0.3560
LDF_travel_LastRegion_z5	1	-0.0177	0.0313	-0.57	0.5718
LDF_travel_LastRegion_z6	1	-0.6465	1.3884	-0.47	0.6415

Table B Estimation Result of the SRUM for Switching Region Sets by DML and Vessel Size of Tuna Purse Seine Fleet in EPO

The MDC Procedure

Conditional Logit Estimates

DML=0 Vessel_Size=2_Median (700-1,050t)

Parameter Estimates

Parameter	DF	Estimate	Standard Error	t Value	Approx Pr > |t|
LDF_travel_LastRegion_z7	1	0.001910	0.0301	0.06	0.9494
LDF_travel_LastRegion_z8	1	-0.0287	0.0280	-1.02	0.3066
LDF_travel_LastRegion_z9	1	0.0181	0.0414	0.44	0.6610
LDF_travel_LastRegion_z10	1	0.0604	0.0348	1.74	0.0826
LDF_travel_LastRegion_z11	1	-0.0608	0.0355	-1.71	0.0871
lDOL_LastRegion_z1	1	0.2490	9812	0.00	1.0000
lDOL_LastRegion_z2	1	5.1020	0.8957	5.70	<.0001
lDOL_LastRegion_z3	1	8.9917	15.4081	0.58	0.5595
lDOL_LastRegion_z4	1	10.2332	1.2745	8.03	<.0001
lDOL_LastRegion_z5	1	10.1351	1.2148	8.34	<.0001
lDOL_LastRegion_z6	1	3.5055	5.3963	0.65	0.5159
lDOL_LastRegion_z7	1	9.9077	1.2779	7.75	<.0001
lDOL_LastRegion_z8	1	9.9913	1.2950	7.72	<.0001
lDOL_LastRegion_z9	1	9.7999	1.3498	7.26	<.0001
lDOL_LastRegion_z10	1	10.0391	1.3116	7.65	<.0001
lDOL_LastRegion_z11	1	8.2072	1.4019	5.85	<.0001
lOBJ_LastRegion_z1	1	-1.0692	41.7671	-0.03	0.9796
lOBJ_LastRegion_z2	1	-0.0946	0.0643	-1.47	0.1414
lOBJ_LastRegion_z3	1	-3.8360	46.5028	-0.08	0.9343
lOBJ_LastRegion_z4	1	-0.0217	0.0326	-0.67	0.5059
lOBJ_LastRegion_z5	1	-0.0418	0.0301	-1.39	0.1657
lOBJ_LastRegion_z6	1	0.4384	0.8529	0.51	0.6072
lOBJ_LastRegion_z7	1	-0.0444	0.0271	-1.64	0.1012
lOBJ_LastRegion_z8	1	0.001246	0.0253	0.05	0.9608
lOBJ_LastRegion_z9	1	-0.0836	0.0354	-2.36	0.0182
lOBJ_LastRegion_z10	1	-0.0339	0.0278	-1.22	0.2233
lOBJ_LastRegion_z11	1	-0.1087	0.0342	-3.18	0.0015
lNOA_LastRegion_z1	1	-1.0291	50.4448	-0.02	0.9837
lNOA_LastRegion_z2	1	-0.1750	0.0657	-2.66	0.0077

Table B Estimation Result of the SRUM for Switching Region Sets by DML and Vessel Size of Tuna Purse Seine Fleet in EPO

The MDC Procedure

Conditional Logit Estimates

DML=0 Vessel_Size=2_Median (700-1,050t)

Parameter Estimates

Parameter	DF	Estimate	Standard Error	t Value	Approx Pr > |t|
lNOA_LastRegion_z3	1	-4.2645	112.4389	-0.04	0.9697
lNOA_LastRegion_z4	1	-0.0444	0.0256	-1.74	0.0827
lNOA_LastRegion_z5	1	-0.0384	0.0235	-1.64	0.1019
lNOA_LastRegion_z6	1	0.0182	0.4100	0.04	0.9646
lNOA_LastRegion_z7	1	0.003369	0.0206	0.16	0.8700
lNOA_LastRegion_z8	1	-0.0383	0.0199	-1.93	0.0539
lNOA_LastRegion_z9	1	-0.0144	0.0275	-0.53	0.5989
lNOA_LastRegion_z10	1	0.008419	0.0219	0.38	0.7013
lNOA_LastRegion_z11	1	0.001575	0.0267	0.06	0.9529
lYFT_LastRegion_z1	1	-0.7402	2301	-0.00	0.9997
lYFT_LastRegion_z2	1	0.0252	0.0354	0.71	0.4760
lYFT_LastRegion_z3	1	2.1810	12.6907	0.17	0.8635
lYFT_LastRegion_z4	1	-0.009947	0.0230	-0.43	0.6654
lYFT_LastRegion_z5	1	-0.009373	0.0204	-0.46	0.6466
lYFT_LastRegion_z6	1	2.9312	7.4782	0.39	0.6951
lYFT_LastRegion_z7	1	-0.0177	0.0197	-0.90	0.3683
lYFT_LastRegion_z8	1	0.009407	0.0188	0.50	0.6177
lYFT_LastRegion_z9	1	0.0482	0.0283	1.70	0.0892
lYFT_LastRegion_z10	1	-0.0205	0.0216	-0.95	0.3429
lYFT_LastRegion_z11	1	0.0486	0.0265	1.83	0.0669
lBET_LastRegion_z1	1	-0.6192	8984	-0.00	0.9999
lBET_LastRegion_z2	1	0.0686	0.0401	1.71	0.0870
lBET_LastRegion_z3	1	-1.0336	4934	-0.00	0.9998
lBET_LastRegion_z4	1	-0.005650	0.0214	-0.26	0.7913
lBET_LastRegion_z5	1	0.0421	0.0202	2.08	0.0374
lBET_LastRegion_z6	1	0.0171	8.8540	0.00	0.9985
lBET_LastRegion_z7	1	0.0203	0.0188	1.08	0.2785
lBET_LastRegion_z8	1	-0.008619	0.0172	-0.50	0.6164
lBET_LastRegion_z9	1	-0.0581	0.0252	-2.31	0.0210

Table B Estimation Result of the SRUM for Switching Region Sets by DML and Vessel Size of Tuna Purse Seine Fleet in EPO

The MDC Procedure

Conditional Logit Estimates

DML=0 Vessel_Size=2_Median (700-1,050t)

Parameter Estimates

Parameter	DF	Estimate	Standard Error	t Value	Approx Pr > |t|
lBET_LastRegion_z10	1	-0.0501	0.0203	-2.46	0.0139
lBET_LastRegion_z11	1	0.002556	0.0254	0.10	0.9198
lSKJ_LastRegion_z1	1	-0.9379	3243	-0.00	0.9998
lSKJ_LastRegion_z2	1	0.0390	0.0436	0.89	0.3711
lSKJ_LastRegion_z3	1	-1.3623	4935	-0.00	0.9998
lSKJ_LastRegion_z4	1	0.0224	0.0254	0.88	0.3783
lSKJ_LastRegion_z5	1	0.0149	0.0227	0.66	0.5112
lSKJ_LastRegion_z6	1	2.3008	4.3662	0.53	0.5982
lSKJ_LastRegion_z7	1	0.007472	0.0211	0.35	0.7238
lSKJ_LastRegion_z8	1	-0.0109	0.0198	-0.55	0.5812
lSKJ_LastRegion_z9	1	0.0148	0.0291	0.51	0.6109
lSKJ_LastRegion_z10	1	0.003941	0.0228	0.17	0.8627
lSKJ_LastRegion_z11	1	0.0118	0.0272	0.44	0.6632
SST_DOL_L	1	0.001222	0.000538	2.27	0.0230
SST_DOL_H	1	-0.004430	0.001236	-3.58	0.0003
O2_DOL_L	1	-0.6796	0.1767	-3.85	0.0001
O2_DOL_H	1	-0.1973	0.0237	-8.31	<.0001
SSH_DOL_L	1	-0.002904	0.001016	-2.86	0.0043
SSH_DOL_H	1	0.001548	0.000876	1.77	0.0772
MLD_DOL_L	1	-0.001108	0.001770	-0.63	0.5312
MLD_DOL_H	1	-0.000935	0.000654	-1.43	0.1526
CHLORO_DOL_L	1	0.004283	0.000713	6.00	<.0001
CHLORO_DOL_H	1	-0.000930	0.002023	-0.46	0.6457
MEI_z1	1	0.2280	1.1994	0.19	0.8492
MEI_z2	1	-0.1241	0.1252	-0.99	0.3216
MEI_z3	1	0.5758	0.6863	0.84	0.4015
MEI_z4	1	-0.2481	0.0885	-2.80	0.0050
MEI_z5	1	-0.0640	0.0808	-0.79	0.4285
MEI_z6	1	1.3398	2.1349	0.63	0.5303

Table B Estimation Result of the SRUM for Switching Region Sets by DML and Vessel Size of Tuna Purse Seine Fleet in EPO

The MDC Procedure

Conditional Logit Estimates

DML=0 Vessel_Size=2_Median (700-1,050t)

Parameter Estimates

Parameter	DF	Estimate	Standard Error	t Value	Approx Pr > |t|
MEI_z7	1	-0.1066	0.0788	-1.35	0.1760
MEI_z8	1	-0.0649	0.0803	-0.81	0.4188
MEI_z9	1	0.0814	0.1126	0.72	0.4700
MEI_z10	1	0.0873	0.0901	0.97	0.3325
MEI_z11	1	0.1671	0.1025	1.63	0.1029

Table B Estimation Result of the SRUM for Switching Region Sets by DML and Vessel Size of Tuna Purse Seine Fleet in EPO

The MDC Procedure

Conditional Logit Estimates

DML=0 Vessel_Size=3_Large (1,050-1,250t)

Algorithm converged.

Model Fit Summary	
Dependent Variable	Decision
Number of Observations	1574
Number of Cases	18888
Log Likelihood	-3013
Log Likelihood Null (LogL(0))	-3911
Maximum Absolute Gradient	10.78181
Number of Iterations	354
Optimization Method	Dual Quasi-Newton
AIC	6383
Schwarz Criterion	7343

Discrete Response Profile

Index	CHOICE	Frequency	Percent
0	1	10	0.64
1	2	176	11.18
2	3	18	1.14
3	4	227	14.42
4	5	295	18.74
5	6	17	1.08
6	7	155	9.85
7	8	235	14.93
8	9	58	3.68
9	10	90	5.72
10	11	68	4.32
11	12	225	14.29

Table B Estimation Result of the SRUM for Switching Region Sets by DML and Vessel Size of Tuna Purse Seine Fleet in EPO

The MDC Procedure

Conditional Logit Estimates

DML=0 Vessel_Size=3_Large (1,050-1,250t)

Goodness-of-Fit Measures		
Measure	Value	Formula
Likelihood Ratio (R)	1797.2	2 * (LogL - LogL0)
Upper Bound of R (U)	7822.5	- 2 * LogL0
Aldrich-Nelson	0.5331	R / (R+N)
Cragg-Uhler 1	0.6808	1 - exp(-R/N)
Cragg-Uhler 2	0.6855	(1-exp(-R/N)) / (1-exp(-U/N))
Estrella	0.7267	1 - (1-R/U)^(U/N)
Adjusted Estrella	0.636	1 - ((LogL-K)/LogL0)^(-2/N*LogL0)
McFadden's LRI	0.2298	R / U
Veall-Zimmermann	0.6404	(R * (U+N)) / (U * (R+N))
N = # of observations, K = # of regressors		

Table B Estimation Result of the SRUM for Switching Region Sets by DML and Vessel Size of Tuna Purse Seine Fleet in EPO

The MDC Procedure

Conditional Logit Estimates

DML=0 Vessel_Size=3_Large (1,050-1,250t)

Parameter Estimates

Parameter	DF	Estimate	Standard Error	t Value	Approx Pr > |t|
LDistant_Expected	1	-0.4974	0.0469	-10.61	<.0001
LDistant_Arrive	1	-0.7289	0.2038	-3.58	0.0003
lCPUE_All_1	1	0.1110	0.1116	0.99	0.3200
lRPUE_All_1	1	-0.0646	0.0708	-0.91	0.3614
LDistant_sinceDep1_z1	1	0.9950	0.5099	1.95	0.0510
LDistant_sinceDep1_z2	1	1.0282	0.3145	3.27	0.0011
LDistant_sinceDep1_z3	1	1.3894	0.9236	1.50	0.1325
LDistant_sinceDep1_z4	1	-0.2037	0.3373	-0.60	0.5459
LDistant_sinceDep1_z5	1	-0.5095	0.2734	-1.86	0.0624
LDistant_sinceDep1_z6	1	-2.0672	1.3848	-1.49	0.1355
LDistant_sinceDep1_z7	1	-0.7769	0.3195	-2.43	0.0150
LDistant_sinceDep1_z8	1	-0.7840	0.3315	-2.36	0.0180
LDistant_sinceDep1_z9	1	-1.7019	0.3752	-4.54	<.0001
LDistant_sinceDep1_z10	1	-1.8764	0.3684	-5.09	<.0001
LDistant_sinceDep1_z11	1	-0.7593	0.4230	-1.80	0.0727
LDF_search_sinceDep1_z1	1	0.0115	0.0943	0.12	0.9031
LDF_search_sinceDep1_z2	1	-1.6868	0.9401	-1.79	0.0728
LDF_search_sinceDep1_z3	1	-0.3518	1.1915	-0.30	0.7678
LDF_search_sinceDep1_z4	1	-0.4761	0.7753	-0.61	0.5391
LDF_search_sinceDep1_z5	1	0.0174	0.6908	0.03	0.9799
LDF_search_sinceDep1_z6	1	1.4488	1.3034	1.11	0.2663
LDF_search_sinceDep1_z7	1	0.3844	0.5518	0.70	0.4861
LDF_search_sinceDep1_z8	1	0.7233	0.4704	1.54	0.1242
LDF_search_sinceDep1_z9	1	2.0999	0.5542	3.79	0.0002
LDF_search_sinceDep1_z10	1	2.5314	0.4767	5.31	<.0001
LDF_search_sinceDep1_z11	1	0.9390	0.4628	2.03	0.0425
LDF_travel_sinceDep1_z1	1	0.4915	0.5970	0.82	0.4103
LDF_travel_sinceDep1_z2	1	-0.007870	0.1311	-0.06	0.9521
LDF_travel_sinceDep1_z3	1	-0.1335	0.2025	-0.66	0.5096

Table B Estimation Result of the SRUM for Switching Region Sets by DML and Vessel Size of Tuna Purse Seine Fleet in EPO

The MDC Procedure

Conditional Logit Estimates

DML=0 Vessel_Size=3_Large (1,050-1,250t)

Parameter Estimates

Parameter	DF	Estimate	Standard Error	t Value	Approx Pr > |t|
LDF_travel_sinceDep1_z4	1	-0.0521	0.1174	-0.44	0.6570
LDF_travel_sinceDep1_z5	1	0.1732	0.1238	1.40	0.1616
LDF_travel_sinceDep1_z6	1	0.9221	0.4517	2.04	0.0412
LDF_travel_sinceDep1_z7	1	0.1669	0.1415	1.18	0.2384
LDF_travel_sinceDep1_z8	1	0.1203	0.1266	0.95	0.3422
LDF_travel_sinceDep1_z9	1	-0.2136	0.1437	-1.49	0.1373
LDF_travel_sinceDep1_z10	1	-0.1483	0.1411	-1.05	0.2933
LDF_travel_sinceDep1_z11	1	-0.0588	0.1386	-0.42	0.6712
lSKJ_sinceDep1_z1	1	-0.0947	0.1294	-0.73	0.4643
lSKJ_sinceDep1_z2	1	0.0583	0.0452	1.29	0.1972
lSKJ_sinceDep1_z3	1	0.0403	0.0779	0.52	0.6052
lSKJ_sinceDep1_z4	1	0.0406	0.0385	1.05	0.2917
lSKJ_sinceDep1_z5	1	0.0331	0.0371	0.89	0.3712
lSKJ_sinceDep1_z6	1	-0.0987	0.0787	-1.25	0.2098
lSKJ_sinceDep1_z7	1	0.001916	0.0372	0.05	0.9589
lSKJ_sinceDep1_z8	1	0.0428	0.0362	1.18	0.2374
lSKJ_sinceDep1_z9	1	0.0324	0.0480	0.67	0.4998
lSKJ_sinceDep1_z10	1	0.0404	0.0427	0.94	0.3451
lSKJ_sinceDep1_z11	1	0.0584	0.0469	1.24	0.2132
lYFT_sinceDep1_z1	1	-0.0922	0.1182	-0.78	0.4355
lYFT_sinceDep1_z2	1	0.0903	0.0424	2.13	0.0331
lYFT_sinceDep1_z3	1	0.0537	0.1228	0.44	0.6622
lYFT_sinceDep1_z4	1	0.0207	0.0367	0.56	0.5732
lYFT_sinceDep1_z5	1	0.002101	0.0351	0.06	0.9523
lYFT_sinceDep1_z6	1	0.2722	0.2548	1.07	0.2854
lYFT_sinceDep1_z7	1	0.0195	0.0398	0.49	0.6247
lYFT_sinceDep1_z8	1	-0.007114	0.0359	-0.20	0.8431
lYFT_sinceDep1_z9	1	0.002401	0.0558	0.04	0.9657
lYFT_sinceDep1_z10	1	-0.0140	0.0488	-0.29	0.7746

Table B Estimation Result of the SRUM for Switching Region Sets by DML and Vessel Size of Tuna Purse Seine Fleet in EPO

The MDC Procedure

Conditional Logit Estimates

DML=0 Vessel_Size=3_Large (1,050-1,250t)

Parameter Estimates

Parameter	DF	Estimate	Standard Error	t Value	Approx Pr > |t|
lYFT_sinceDep1_z11	1	-0.0200	0.0519	-0.38	0.7006
lBET_sinceDep1_z1	1	-0.1102	0.1104	-1.00	0.3183
lBET_sinceDep1_z2	1	0.0273	0.0333	0.82	0.4128
lBET_sinceDep1_z3	1	-0.2488	0.0785	-3.17	0.0015
lBET_sinceDep1_z4	1	0.0367	0.0295	1.25	0.2127
lBET_sinceDep1_z5	1	-0.004187	0.0287	-0.15	0.8842
lBET_sinceDep1_z6	1	-0.1809	0.0822	-2.20	0.0277
lBET_sinceDep1_z7	1	-0.0273	0.0309	-0.88	0.3779
lBET_sinceDep1_z8	1	-0.0502	0.0288	-1.74	0.0814
lBET_sinceDep1_z9	1	-0.0178	0.0384	-0.46	0.6434
lBET_sinceDep1_z10	1	-0.0736	0.0335	-2.19	0.0283
lBET_sinceDep1_z11	1	-0.1102	0.0386	-2.86	0.0043
LDF_search_LastRegion_z1	1	-0.0715	0.1865	-0.38	0.7014
LDF_search_LastRegion_z2	1	0.0332	0.0528	0.63	0.5290
LDF_search_LastRegion_z3	1	0.0384	0.1222	0.31	0.7531
LDF_search_LastRegion_z4	1	0.0799	0.0520	1.54	0.1243
LDF_search_LastRegion_z5	1	0.005366	0.0428	0.13	0.9003
LDF_search_LastRegion_z6	1	-0.004765	0.1264	-0.04	0.9699
LDF_search_LastRegion_z7	1	0.0244	0.0527	0.46	0.6433
LDF_search_LastRegion_z8	1	0.0973	0.0470	2.07	0.0385
LDF_search_LastRegion_z9	1	-0.0259	0.0684	-0.38	0.7054
LDF_search_LastRegion_z10	1	0.0320	0.0635	0.50	0.6148
LDF_search_LastRegion_z11	1	0.1071	0.0670	1.60	0.1101
LDF_travel_LastRegion_z1	1	-0.002067	0.1792	-0.01	0.9908
LDF_travel_LastRegion_z2	1	0.0719	0.0540	1.33	0.1830
LDF_travel_LastRegion_z3	1	-0.1534	0.1077	-1.42	0.1543
LDF_travel_LastRegion_z4	1	0.0996	0.0501	1.99	0.0467
LDF_travel_LastRegion_z5	1	0.0549	0.0431	1.27	0.2034
LDF_travel_LastRegion_z6	1	-0.0206	0.1285	-0.16	0.8728

Table B Estimation Result of the SRUM for Switching Region Sets by DML and Vessel Size of Tuna Purse Seine Fleet in EPO

The MDC Procedure

Conditional Logit Estimates

DML=0 Vessel_Size=3_Large (1,050-1,250t)

Parameter Estimates

Parameter	DF	Estimate	Standard Error	t Value	Approx Pr > |t|
LDF_travel_LastRegion_z7	1	0.0622	0.0530	1.17	0.2408
LDF_travel_LastRegion_z8	1	0.0135	0.0437	0.31	0.7579
LDF_travel_LastRegion_z9	1	0.1061	0.0736	1.44	0.1497
LDF_travel_LastRegion_z10	1	0.0903	0.0636	1.42	0.1558
LDF_travel_LastRegion_z11	1	-0.0381	0.0608	-0.63	0.5312
lDOL_LastRegion_z1	1	0.3346	0.1378	2.43	0.0152
lDOL_LastRegion_z2	1	0.0534	0.1115	0.48	0.6324
lDOL_LastRegion_z3	1	0.2666	0.1177	2.26	0.0236
lDOL_LastRegion_z4	1	0.2194	0.0757	2.90	0.0038
lDOL_LastRegion_z5	1	0.1301	0.0771	1.69	0.0914
lDOL_LastRegion_z6	1	0.1244	0.1494	0.83	0.4048
lDOL_LastRegion_z7	1	0.2331	0.0760	3.07	0.0022
lDOL_LastRegion_z8	1	0.1804	0.0752	2.40	0.0165
lDOL_LastRegion_z9	1	0.1664	0.0881	1.89	0.0589
lDOL_LastRegion_z10	1	0.0913	0.0889	1.03	0.3045
lDOL_LastRegion_z11	1	-0.0807	0.1165	-0.69	0.4884
lOBJ_LastRegion_z1	1	-0.0681	0.1527	-0.45	0.6556
lOBJ_LastRegion_z2	1	0.0894	0.0611	1.46	0.1437
lOBJ_LastRegion_z3	1	-0.1110	0.0984	-1.13	0.2597
lOBJ_LastRegion_z4	1	0.0217	0.0530	0.41	0.6826
lOBJ_LastRegion_z5	1	0.0274	0.0511	0.54	0.5920
lOBJ_LastRegion_z6	1	0.1642	0.1122	1.46	0.1431
lOBJ_LastRegion_z7	1	-0.0117	0.0538	-0.22	0.8280
lOBJ_LastRegion_z8	1	0.0133	0.0506	0.26	0.7933
lOBJ_LastRegion_z9	1	-0.0510	0.0665	-0.77	0.4433
lOBJ_LastRegion_z10	1	0.0327	0.0581	0.56	0.5740
lOBJ_LastRegion_z11	1	-0.0110	0.0701	-0.16	0.8751
lNOA_LastRegion_z1	1	-0.0266	0.1249	-0.21	0.8312
lNOA_LastRegion_z2	1	-0.0510	0.0504	-1.01	0.3113

Table B Estimation Result of the SRUM for Switching Region Sets by DML and Vessel Size of Tuna Purse Seine Fleet in EPO

The MDC Procedure

Conditional Logit Estimates

DML=0 Vessel_Size=3_Large (1,050-1,250t)

Parameter Estimates

Parameter	DF	Estimate	Standard Error	t Value	Approx Pr > |t|
lNOA_LastRegion_z3	1	0.1229	0.0899	1.37	0.1713
lNOA_LastRegion_z4	1	-0.0334	0.0412	-0.81	0.4171
lNOA_LastRegion_z5	1	0.002129	0.0373	0.06	0.9545
lNOA_LastRegion_z6	1	-0.1773	0.1302	-1.36	0.1734
lNOA_LastRegion_z7	1	0.0338	0.0399	0.85	0.3979
lNOA_LastRegion_z8	1	0.0160	0.0370	0.43	0.6653
lNOA_LastRegion_z9	1	0.0459	0.0519	0.88	0.3765
lNOA_LastRegion_z10	1	0.0167	0.0450	0.37	0.7101
lNOA_LastRegion_z11	1	0.0122	0.0521	0.23	0.8147
lYFT_LastRegion_z1	1	-0.0205	0.1114	-0.18	0.8541
lYFT_LastRegion_z2	1	-0.0680	0.0363	-1.88	0.0607
lYFT_LastRegion_z3	1	-0.002042	0.0935	-0.02	0.9826
lYFT_LastRegion_z4	1	-0.0220	0.0344	-0.64	0.5226
lYFT_LastRegion_z5	1	-0.005465	0.0326	-0.17	0.8669
lYFT_LastRegion_z6	1	0.003519	0.1182	0.03	0.9763
lYFT_LastRegion_z7	1	-0.0280	0.0369	-0.76	0.4472
lYFT_LastRegion_z8	1	-0.0139	0.0334	-0.42	0.6767
lYFT_LastRegion_z9	1	0.0113	0.0521	0.22	0.8277
lYFT_LastRegion_z10	1	0.0123	0.0441	0.28	0.7806
lYFT_LastRegion_z11	1	0.0980	0.0534	1.83	0.0666
lBET_LastRegion_z1	1	0.0137	0.1194	0.11	0.9089
lBET_LastRegion_z2	1	-0.0151	0.0316	-0.48	0.6315
lBET_LastRegion_z3	1	0.0191	0.1052	0.18	0.8555
lBET_LastRegion_z4	1	-0.0440	0.0286	-1.54	0.1240
lBET_LastRegion_z5	1	0.0352	0.0288	1.22	0.2211
lBET_LastRegion_z6	1	-1.6763	1.1748	-1.43	0.1536
lBET_LastRegion_z7	1	0.0166	0.0332	0.50	0.6176
lBET_LastRegion_z8	1	0.0667	0.0305	2.19	0.0285
lBET_LastRegion_z9	1	-0.0723	0.0460	-1.57	0.1166

Table B Estimation Result of the SRUM for Switching Region Sets by DML and Vessel Size of Tuna Purse Seine Fleet in EPO

The MDC Procedure

Conditional Logit Estimates

DML=0 Vessel_Size=3_Large (1,050-1,250t)

Parameter Estimates

Parameter	DF	Estimate	Standard Error	t Value	Approx Pr > |t|
lBET_LastRegion_z10	1	-0.0581	0.0380	-1.53	0.1262
lBET_LastRegion_z11	1	0.0500	0.0430	1.16	0.2450
lSKJ_LastRegion_z1	1	0.1790	0.1408	1.27	0.2035
lSKJ_LastRegion_z2	1	-0.004107	0.0476	-0.09	0.9312
lSKJ_LastRegion_z3	1	0.0768	0.0839	0.92	0.3598
lSKJ_LastRegion_z4	1	0.006038	0.0418	0.14	0.8851
lSKJ_LastRegion_z5	1	-0.0448	0.0394	-1.13	0.2566
lSKJ_LastRegion_z6	1	-0.0439	0.1052	-0.42	0.6765
lSKJ_LastRegion_z7	1	-0.0210	0.0429	-0.49	0.6250
lSKJ_LastRegion_z8	1	-0.0852	0.0390	-2.18	0.0290
lSKJ_LastRegion_z9	1	-0.0134	0.0553	-0.24	0.8079
lSKJ_LastRegion_z10	1	-0.0776	0.0478	-1.63	0.1040
lSKJ_LastRegion_z11	1	-0.1287	0.0513	-2.51	0.0121
SST_DOL_L	1	-0.000454	0.000706	-0.64	0.5206
SST_DOL_H	1	0.002697	0.001718	1.57	0.1165
O2_DOL_L	1	-0.2224	0.0615	-3.62	0.0003
O2_DOL_H	1	-0.0205	0.005099	-4.02	<.0001
SSH_DOL_L	1	-0.005015	0.001585	-3.16	0.0016
SSH_DOL_H	1	0.004395	0.001082	4.06	<.0001
MLD_DOL_L	1	-0.004606	0.003061	-1.50	0.1324
MLD_DOL_H	1	-0.002755	0.000812	-3.39	0.0007
CHLORO_DOL_L	1	0.003553	0.000923	3.85	0.0001
CHLORO_DOL_H	1	0.005704	0.003147	1.81	0.0700
MEI_z1	1	-0.5821	0.5810	-1.00	0.3164
MEI_z2	1	-0.3436	0.1540	-2.23	0.0256
MEI_z3	1	1.5463	0.3465	4.46	<.0001
MEI_z4	1	-0.2512	0.1294	-1.94	0.0523
MEI_z5	1	-0.1451	0.1201	-1.21	0.2273
MEI_z6	1	1.2861	0.3731	3.45	0.0006

Table B Estimation Result of the SRUM for Switching Region Sets by DML and Vessel Size of Tuna Purse Seine Fleet in EPO

The MDC Procedure

Conditional Logit Estimates

DML=0 Vessel_Size=3_Large (1,050-1,250t)

Parameter Estimates

Parameter	DF	Estimate	Standard Error	t Value	Approx Pr > |t|
MEI_z7	1	-0.0721	0.1408	-0.51	0.6088
MEI_z8	1	-0.0308	0.1291	-0.24	0.8114
MEI_z9	1	0.2099	0.2042	1.03	0.3041
MEI_z10	1	0.1531	0.1791	0.85	0.3926
MEI_z11	1	0.4595	0.1785	2.57	0.0101

Table B Estimation Result of the SRUM for Switching Region Sets by DML and Vessel Size of Tuna Purse Seine Fleet in EPO

The MDC Procedure

Conditional Logit Estimates

DML=0 Vessel_Size=4_XLarge (1,250-1,800t)

Algorithm converged.

Model Fit Summary	
Dependent Variable	Decision
Number of Observations	2266
Number of Cases	27192
Log Likelihood	-4179
Log Likelihood Null (LogL(0))	-5631
Maximum Absolute Gradient	6.42112
Number of Iterations	465
Optimization Method	Dual Quasi-Newton
AIC	8715
Schwarz Criterion	9740

Discrete Response Profile			
Index	CHOICE	Frequency	Percent
0	1	12	0.53
1	2	400	17.65
2	3	9	0.40
3	4	453	19.99
4	5	456	20.12
5	6	5	0.22
6	7	224	9.89
7	8	239	10.55
8	9	34	1.50
9	10	95	4.19
10	11	105	4.63
11	12	234	10.33

Table B Estimation Result of the SRUM for Switching Region Sets by DML and Vessel Size of Tuna Purse Seine Fleet in EPO

The MDC Procedure

Conditional Logit Estimates

DML=0 Vessel_Size=4_XLarge (1,250-1,800t)

Goodness-of-Fit Measures		
Measure	Value	Formula
Likelihood Ratio (R)	2904.2	2 * (LogL - LogL0)
Upper Bound of R (U)	11262	- 2 * LogL0
Aldrich-Nelson	0.5617	R / (R+N)
Cragg-Uhler 1	0.7224	1 - exp(-R/N)
Cragg-Uhler 2	0.7275	(1-exp(-R/N)) / (1-exp(-U/N))
Estrella	0.7729	1 - (1-R/U)^(U/N)
Adjusted Estrella	0.7202	1 - ((LogL-K)/LogL0)^(-2/N*LogL0)
McFadden's LRI	0.2579	R / U
Veall-Zimmermann	0.6747	(R * (U+N)) / (U * (R+N))
N = # of observations, K = # of regressors		

Table B Estimation Result of the SRUM for Switching Region Sets by DML and Vessel Size of Tuna Purse Seine Fleet in EPO

The MDC Procedure

Conditional Logit Estimates

DML=0 Vessel_Size=4_XLarge (1,250-1,800t)

Parameter Estimates

Parameter	DF	Estimate	Standard Error	t Value	Approx Pr > |t|
LDistant_Expected	1	-0.2646	0.0396	-6.69	<.0001
LDistant_Arrive	1	-0.5650	0.1959	-2.88	0.0039
lCPUE_All_1	1	0.2994	0.1022	2.93	0.0034
lRPUE_All_1	1	-0.1797	0.0647	-2.78	0.0055
LDistant_sinceDep1_z1	1	5.6184	1.7934	3.13	0.0017
LDistant_sinceDep1_z2	1	2.1022	0.4721	4.45	<.0001
LDistant_sinceDep1_z3	1	3.9429	2.0185	1.95	0.0508
LDistant_sinceDep1_z4	1	1.1160	0.4514	2.47	0.0134
LDistant_sinceDep1_z5	1	0.1471	0.4450	0.33	0.7410
LDistant_sinceDep1_z6	1	-3.3810	2.8996	-1.17	0.2436
LDistant_sinceDep1_z7	1	-0.4014	0.4276	-0.94	0.3479
LDistant_sinceDep1_z8	1	-0.3927	0.4597	-0.85	0.3929
LDistant_sinceDep1_z9	1	-2.1102	0.6048	-3.49	0.0005
LDistant_sinceDep1_z10	1	-2.6633	0.5652	-4.71	<.0001
LDistant_sinceDep1_z11	1	-1.1662	0.6009	-1.94	0.0523
LDF_search_sinceDep1_z1	1	0.3905	0.1460	2.68	0.0075
LDF_search_sinceDep1_z2	1	1.6767	1.4429	1.16	0.2452
LDF_search_sinceDep1_z3	1	-1.1371	2.2241	-0.51	0.6092
LDF_search_sinceDep1_z4	1	1.6494	1.1633	1.42	0.1562
LDF_search_sinceDep1_z5	1	2.3636	1.0306	2.29	0.0218
LDF_search_sinceDep1_z6	1	3.9126	2.9960	1.31	0.1916
LDF_search_sinceDep1_z7	1	1.9358	0.7758	2.50	0.0126
LDF_search_sinceDep1_z8	1	2.2543	0.6643	3.39	0.0007
LDF_search_sinceDep1_z9	1	3.3048	0.7742	4.27	<.0001
LDF_search_sinceDep1_z10	1	3.5575	0.5932	6.00	<.0001
LDF_search_sinceDep1_z11	1	1.6697	0.5523	3.02	0.0025
LDF_travel_sinceDep1_z1	1	-0.7876	0.5919	-1.33	0.1833
LDF_travel_sinceDep1_z2	1	-0.3172	0.1556	-2.04	0.0415
LDF_travel_sinceDep1_z3	1	0.3760	0.6207	0.61	0.5446

Table B Estimation Result of the SRUM for Switching Region Sets by DML and Vessel Size of Tuna Purse Seine Fleet in EPO

The MDC Procedure

Conditional Logit Estimates

DML=0 Vessel_Size=4_XLarge (1,250-1,800t)

Parameter Estimates

Parameter	DF	Estimate	Standard Error	t Value	Approx Pr > |t|
LDF_travel_sinceDep1_z4	1	-0.1243	0.1502	-0.83	0.4080
LDF_travel_sinceDep1_z5	1	-0.1907	0.1485	-1.28	0.1992
LDF_travel_sinceDep1_z6	1	-0.5880	0.9403	-0.63	0.5318
LDF_travel_sinceDep1_z7	1	-0.1283	0.1610	-0.80	0.4255
LDF_travel_sinceDep1_z8	1	-0.3420	0.1558	-2.20	0.0281
LDF_travel_sinceDep1_z9	1	-0.3070	0.2268	-1.35	0.1758
LDF_travel_sinceDep1_z10	1	-0.1047	0.1955	-0.54	0.5923
LDF_travel_sinceDep1_z11	1	0.1000	0.2050	0.49	0.6255
lSKJ_sinceDep1_z1	1	0.0170	0.2649	0.06	0.9487
lSKJ_sinceDep1_z2	1	0.0466	0.0446	1.04	0.2969
lSKJ_sinceDep1_z3	1	-0.0417	0.1614	-0.26	0.7962
lSKJ_sinceDep1_z4	1	0.0163	0.0404	0.40	0.6862
lSKJ_sinceDep1_z5	1	-0.0389	0.0382	-1.02	0.3079
lSKJ_sinceDep1_z6	1	-0.1151	0.1729	-0.67	0.5055
lSKJ_sinceDep1_z7	1	-0.0212	0.0425	-0.50	0.6174
lSKJ_sinceDep1_z8	1	-0.0430	0.0413	-1.04	0.2969
lSKJ_sinceDep1_z9	1	-0.0928	0.0605	-1.53	0.1252
lSKJ_sinceDep1_z10	1	-0.0619	0.0510	-1.21	0.2248
lSKJ_sinceDep1_z11	1	-0.0814	0.0446	-1.82	0.0682
lYFT_sinceDep1_z1	1	0.4457	0.2996	1.49	0.1368
lYFT_sinceDep1_z2	1	0.0557	0.0336	1.66	0.0968
lYFT_sinceDep1_z3	1	0.6354	0.4160	1.53	0.1266
lYFT_sinceDep1_z4	1	0.0351	0.0318	1.10	0.2699
lYFT_sinceDep1_z5	1	0.002009	0.0310	0.06	0.9484
lYFT_sinceDep1_z6	1	1.6608	0.9645	1.72	0.0851
lYFT_sinceDep1_z7	1	0.0626	0.0360	1.74	0.0826
lYFT_sinceDep1_z8	1	0.0382	0.0358	1.07	0.2857
lYFT_sinceDep1_z9	1	0.1832	0.0895	2.05	0.0407
lYFT_sinceDep1_z10	1	0.0786	0.0511	1.54	0.1243

Table B Estimation Result of the SRUM for Switching Region Sets by DML and Vessel Size of Tuna Purse Seine Fleet in EPO

The MDC Procedure

Conditional Logit Estimates

DML=0 Vessel_Size=4_XLarge (1,250-1,800t)

Parameter Estimates

Parameter	DF	Estimate	Standard Error	t Value	Approx Pr > |t|
lYFT_sinceDep1_z11	1	0.0870	0.0427	2.04	0.0415
lBET_sinceDep1_z1	1	0.0827	0.0944	0.88	0.3811
lBET_sinceDep1_z2	1	0.000758	0.0259	0.03	0.9766
lBET_sinceDep1_z3	1	0.1023	0.1026	1.00	0.3186
lBET_sinceDep1_z4	1	0.0200	0.0244	0.82	0.4108
lBET_sinceDep1_z5	1	0.0536	0.0252	2.13	0.0335
lBET_sinceDep1_z6	1	0.1622	0.1631	0.99	0.3199
lBET_sinceDep1_z7	1	-0.001344	0.0264	-0.05	0.9593
lBET_sinceDep1_z8	1	-0.0103	0.0260	-0.40	0.6906
lBET_sinceDep1_z9	1	-0.0363	0.0458	-0.79	0.4289
lBET_sinceDep1_z10	1	-0.0688	0.0305	-2.25	0.0242
lBET_sinceDep1_z11	1	-0.0945	0.0293	-3.22	0.0013
LDF_search_LastRegion_z1	1	-0.0307	0.2907	-0.11	0.9158
LDF_search_LastRegion_z2	1	0.0321	0.0472	0.68	0.4973
LDF_search_LastRegion_z3	1	0.002721	0.1258	0.02	0.9828
LDF_search_LastRegion_z4	1	0.0772	0.0445	1.74	0.0826
LDF_search_LastRegion_z5	1	0.0279	0.0434	0.64	0.5198
LDF_search_LastRegion_z6	1	-0.6323	0.4208	-1.50	0.1330
LDF_search_LastRegion_z7	1	0.002909	0.0470	0.06	0.9506
LDF_search_LastRegion_z8	1	-0.0194	0.0505	-0.38	0.7011
LDF_search_LastRegion_z9	1	-0.0235	0.0890	-0.26	0.7916
LDF_search_LastRegion_z10	1	0.000365	0.0599	0.01	0.9951
LDF_search_LastRegion_z11	1	0.0714	0.0564	1.27	0.2057
LDF_travel_LastRegion_z1	1	0.3057	0.2893	1.06	0.2907
LDF_travel_LastRegion_z2	1	0.0919	0.0438	2.10	0.0359
LDF_travel_LastRegion_z3	1	-0.1690	0.1264	-1.34	0.1813
LDF_travel_LastRegion_z4	1	0.0233	0.0389	0.60	0.5488
LDF_travel_LastRegion_z5	1	0.0780	0.0399	1.96	0.0505
LDF_travel_LastRegion_z6	1	1.1324	0.6448	1.76	0.0790

Table B Estimation Result of the SRUM for Switching Region Sets by DML and Vessel Size of Tuna Purse Seine Fleet in EPO

The MDC Procedure

Conditional Logit Estimates

DML=0 Vessel_Size=4_XLarge (1,250-1,800t)

Parameter Estimates

Parameter	DF	Estimate	Standard Error	t Value	Approx Pr > |t|
LDF_travel_LastRegion_z7	1	0.0365	0.0442	0.83	0.4085
LDF_travel_LastRegion_z8	1	0.1250	0.0481	2.60	0.0093
LDF_travel_LastRegion_z9	1	0.1185	0.0922	1.28	0.1989
LDF_travel_LastRegion_z10	1	0.0381	0.0548	0.70	0.4864
LDF_travel_LastRegion_z11	1	0.001517	0.0497	0.03	0.9757
lDOL_LastRegion_z1	1	-0.7034	2.9921	-0.24	0.8141
lDOL_LastRegion_z2	1	-0.5080	1.7864	-0.28	0.7761
lDOL_LastRegion_z3	1	-1.2050	1262	-0.00	0.9992
lDOL_LastRegion_z4	1	4.9627	2.0928	2.37	0.0177
lDOL_LastRegion_z5	1	5.1091	1.9930	2.56	0.0104
lDOL_LastRegion_z6	1	-3.2295	4.5342	-0.71	0.4763
lDOL_LastRegion_z7	1	5.4813	2.1649	2.53	0.0113
lDOL_LastRegion_z8	1	5.3565	2.1671	2.47	0.0134
lDOL_LastRegion_z9	1	5.4768	2.1700	2.52	0.0116
lDOL_LastRegion_z10	1	5.0329	2.3479	2.14	0.0321
lDOL_LastRegion_z11	1	2.6675	2.3408	1.14	0.2545
lOBJ_LastRegion_z1	1	-0.1402	0.1825	-0.77	0.4422
lOBJ_LastRegion_z2	1	-0.1685	0.0517	-3.26	0.0011
lOBJ_LastRegion_z3	1	0.0624	0.2359	0.26	0.7915
lOBJ_LastRegion_z4	1	-0.0549	0.0443	-1.24	0.2150
lOBJ_LastRegion_z5	1	-0.1606	0.0456	-3.52	0.0004
lOBJ_LastRegion_z6	1	0.4539	0.2500	1.82	0.0694
lOBJ_LastRegion_z7	1	-0.0867	0.0498	-1.74	0.0819
lOBJ_LastRegion_z8	1	-0.0994	0.0472	-2.11	0.0351
lOBJ_LastRegion_z9	1	-0.0960	0.0836	-1.15	0.2510
lOBJ_LastRegion_z10	1	-0.0535	0.0545	-0.98	0.3269
lOBJ_LastRegion_z11	1	-0.2107	0.0587	-3.59	0.0003
lNOA_LastRegion_z1	1	-0.0600	0.1378	-0.44	0.6634
lNOA_LastRegion_z2	1	-0.1138	0.0399	-2.85	0.0044

Table B Estimation Result of the SRUM for Switching Region Sets by DML and Vessel Size of Tuna Purse Seine Fleet in EPO

The MDC Procedure

Conditional Logit Estimates

DML=0 Vessel_Size=4_XLarge (1,250-1,800t)

Parameter Estimates

Parameter	DF	Estimate	Standard Error	t Value	Approx Pr > |t|
lNOA_LastRegion_z3	1	-1.8858	1262	-0.00	0.9988
lNOA_LastRegion_z4	1	-0.0484	0.0337	-1.44	0.1511
lNOA_LastRegion_z5	1	-0.0258	0.0327	-0.79	0.4310
lNOA_LastRegion_z6	1	0.1121	0.1586	0.71	0.4795
lNOA_LastRegion_z7	1	-0.0143	0.0371	-0.39	0.6993
lNOA_LastRegion_z8	1	-0.0118	0.0353	-0.33	0.7378
lNOA_LastRegion_z9	1	-0.000583	0.0676	-0.01	0.9931
lNOA_LastRegion_z10	1	0.0263	0.0419	0.63	0.5300
lNOA_LastRegion_z11	1	0.0116	0.0464	0.25	0.8027
lYFT_LastRegion_z1	1	-0.1338	0.0892	-1.50	0.1337
lYFT_LastRegion_z2	1	-0.0497	0.0294	-1.69	0.0908
lYFT_LastRegion_z3	1	-0.0824	0.1211	-0.68	0.4961
lYFT_LastRegion_z4	1	-0.0368	0.0281	-1.31	0.1900
lYFT_LastRegion_z5	1	-0.0199	0.0282	-0.71	0.4802
lYFT_LastRegion_z6	1	-0.2251	0.1543	-1.46	0.1447
lYFT_LastRegion_z7	1	-0.0524	0.0316	-1.66	0.0972
lYFT_LastRegion_z8	1	-0.0380	0.0313	-1.21	0.2250
lYFT_LastRegion_z9	1	-0.0492	0.0632	-0.78	0.4360
lYFT_LastRegion_z10	1	-0.0677	0.0402	-1.69	0.0920
lYFT_LastRegion_z11	1	-0.0823	0.0406	-2.03	0.0428
lBET_LastRegion_z1	1	-0.1887	0.0660	-2.86	0.0043
lBET_LastRegion_z2	1	0.0660	0.0264	2.50	0.0125
lBET_LastRegion_z3	1	-0.2862	0.1078	-2.65	0.0079
lBET_LastRegion_z4	1	0.000879	0.0233	0.04	0.9699
lBET_LastRegion_z5	1	0.0544	0.0249	2.19	0.0287
lBET_LastRegion_z6	1	-0.2004	0.1494	-1.34	0.1798
lBET_LastRegion_z7	1	0.0110	0.0268	0.41	0.6814
lBET_LastRegion_z8	1	0.0114	0.0261	0.44	0.6619
lBET_LastRegion_z9	1	-0.0119	0.0545	-0.22	0.8268

Table B Estimation Result of the SRUM for Switching Region Sets by DML and Vessel Size of Tuna Purse Seine Fleet in EPO

The MDC Procedure

Conditional Logit Estimates

DML=0 Vessel_Size=4_XLarge (1,250-1,800t)

Parameter Estimates

Parameter	DF	Estimate	Standard Error	t Value	Approx Pr > |t|
lBET_LastRegion_z10	1	-0.0659	0.0350	-1.88	0.0595
lBET_LastRegion_z11	1	-0.0281	0.0380	-0.74	0.4606
lSKJ_LastRegion_z1	1	0.3237	0.1430	2.26	0.0236
lSKJ_LastRegion_z2	1	0.1162	0.0384	3.02	0.0025
lSKJ_LastRegion_z3	1	0.1884	0.1743	1.08	0.2798
lSKJ_LastRegion_z4	1	0.0705	0.0327	2.16	0.0311
lSKJ_LastRegion_z5	1	0.0723	0.0331	2.19	0.0289
lSKJ_LastRegion_z6	1	-0.1071	0.1405	-0.76	0.4461
lSKJ_LastRegion_z7	1	0.0732	0.0373	1.97	0.0494
lSKJ_LastRegion_z8	1	0.0552	0.0355	1.56	0.1197
lSKJ_LastRegion_z9	1	0.005531	0.0628	0.09	0.9299
lSKJ_LastRegion_z10	1	0.0694	0.0448	1.55	0.1215
lSKJ_LastRegion_z11	1	0.1250	0.0481	2.60	0.0093
SST_DOL_L	1	-0.001127	0.000572	-1.97	0.0490
SST_DOL_H	1	0.001094	0.001410	0.78	0.4377
O2_DOL_L	1	-1.1370	0.5013	-2.27	0.0233
O2_DOL_H	1	-0.1202	0.0412	-2.92	0.0035
SSH_DOL_L	1	-0.003316	0.001357	-2.44	0.0145
SSH_DOL_H	1	0.006827	0.000903	7.56	<.0001
MLD_DOL_L	1	-0.005462	0.002518	-2.17	0.0301
MLD_DOL_H	1	-0.001880	0.000680	-2.76	0.0057
CHLORO_DOL_L	1	0.003141	0.000667	4.71	<.0001
CHLORO_DOL_H	1	0.001589	0.002576	0.62	0.5373
MEI_z1	1	0.8088	0.4804	1.68	0.0923
MEI_z2	1	-0.5357	0.1265	-4.24	<.0001
MEI_z3	1	0.3757	0.5118	0.73	0.4630
MEI_z4	1	-0.1249	0.1051	-1.19	0.2347
MEI_z5	1	-0.0933	0.1054	-0.89	0.3760
MEI_z6	1	-0.4539	0.8197	-0.55	0.5797

Table B Estimation Result of the SRUM for Switching Region Sets by DML and Vessel Size of Tuna Purse Seine Fleet in EPO

The MDC Procedure

Conditional Logit Estimates

DML=0 Vessel_Size=4_XLarge (1,250-1,800t)

Parameter Estimates

Parameter	DF	Estimate	Standard Error	t Value	Approx Pr > |t|
MEI_z7	1	-0.0295	0.1202	-0.25	0.8060
MEI_z8	1	0.1208	0.1219	0.99	0.3216
MEI_z9	1	0.3039	0.2505	1.21	0.2251
MEI_z10	1	0.3042	0.1653	1.84	0.0658
MEI_z11	1	0.2153	0.1567	1.37	0.1694

Table B Estimation Result of the SRUM for Switching Region Sets by DML and Vessel Size of Tuna Purse Seine Fleet in EPO

The MDC Procedure

Conditional Logit Estimates

DML=1 Vessel_Size=1_Small (363-700t)

Algorithm converged.

Model Fit Summary	
Dependent Variable	Decision
Number of Observations	855
Number of Cases	10260
Log Likelihood	-1369
Log Likelihood Null (LogL(0))	-2125
Maximum Absolute Gradient	8.58080
Number of Iterations	431
Optimization Method	Dual Quasi-Newton
AIC	3097
Schwarz Criterion	3947

Discrete Response Profile			
Index	CHOICE	Frequency	Percent
0	1	29	3.39
1	2	10	1.17
2	3	142	16.61
3	4	76	8.89
4	5	37	4.33
5	6	106	12.40
6	7	134	15.67
7	8	125	14.62
8	9	62	7.25
9	10	106	12.40
10	11	22	2.57
11	12	6	0.70

Table B Estimation Result of the SRUM for Switching Region Sets by DML and Vessel Size of Tuna Purse Seine Fleet in EPO

The MDC Procedure

Conditional Logit Estimates

DML=1 Vessel_Size=1_Small (363-700t)

Goodness-of-Fit Measures		
Measure	Value	Formula
Likelihood Ratio (R)	1510.2	2 * (LogL - LogL0)
Upper Bound of R (U)	4249.2	- 2 * LogL0
Aldrich-Nelson	0.6385	R / (R+N)
Cragg-Uhler 1	0.829	1 - exp(-R/N)
Cragg-Uhler 2	0.8348	(1-exp(-R/N)) / (1-exp(-U/N))
Estrella	0.8872	1 - (1-R/U)^(U/N)
Adjusted Estrella	0.7924	1 - ((LogL-K)/LogL0)^(-2/N*LogL0)
McFadden's LRI	0.3554	R / U
Veall-Zimmermann	0.767	(R * (U+N)) / (U * (R+N))
N = # of observations, K = # of regressors		

Table B Estimation Result of the SRUM for Switching Region Sets by DML and Vessel Size of Tuna Purse Seine Fleet in EPO

The MDC Procedure

Conditional Logit Estimates

DML=1 Vessel_Size=1_Small (363-700t)

Parameter Estimates

Parameter	DF	Estimate	Standard Error	t Value	Approx Pr > |t|
LDistant_Expected	1	-0.4091	0.0698	-5.86	<.0001
LDistant_Arrive	1	-1.9818	0.1727	-11.48	<.0001
lCPUE_All_1	1	0.0221	0.1795	0.12	0.9019
lRPUE_All_1	1	-0.0106	0.1165	-0.09	0.9277
LDistant_sinceDep1_z1	1	-1.2681	2.1821	-0.58	0.5611
LDistant_sinceDep1_z2	1	-0.3983	2.3515	-0.17	0.8655
LDistant_sinceDep1_z3	1	-1.5805	2.0852	-0.76	0.4485
LDistant_sinceDep1_z4	1	-1.5201	2.0526	-0.74	0.4590
LDistant_sinceDep1_z5	1	-0.5277	2.0687	-0.26	0.7986
LDistant_sinceDep1_z6	1	-2.3441	2.0753	-1.13	0.2587
LDistant_sinceDep1_z7	1	-1.4824	2.0509	-0.72	0.4698
LDistant_sinceDep1_z8	1	-1.5907	2.0506	-0.78	0.4379
LDistant_sinceDep1_z9	1	-2.4561	2.0574	-1.19	0.2326
LDistant_sinceDep1_z10	1	-2.5184	2.0558	-1.23	0.2206
LDistant_sinceDep1_z11	1	-2.3883	2.0620	-1.16	0.2468
LDF_search_sinceDep1_z1	1	0.0894	0.1664	0.54	0.5910
LDF_search_sinceDep1_z2	1	0.1145	1.2672	0.09	0.9280
LDF_search_sinceDep1_z3	1	0.6102	0.6846	0.89	0.3727
LDF_search_sinceDep1_z4	1	0.2193	0.7330	0.30	0.7648
LDF_search_sinceDep1_z5	1	-1.0461	0.8606	-1.22	0.2241
LDF_search_sinceDep1_z6	1	0.5737	0.9297	0.62	0.5371
LDF_search_sinceDep1_z7	1	-0.1169	1.0027	-0.12	0.9072
LDF_search_sinceDep1_z8	1	-0.0264	1.1363	-0.02	0.9815
LDF_search_sinceDep1_z9	1	0.8374	1.3090	0.64	0.5224
LDF_search_sinceDep1_z10	1	0.7400	1.4295	0.52	0.6047
LDF_search_sinceDep1_z11	1	0.2984	1.6643	0.18	0.8577
LDF_travel_sinceDep1_z1	1	0.5089	0.7139	0.71	0.4760
LDF_travel_sinceDep1_z2	1	0.1073	0.7689	0.14	0.8891
LDF_travel_sinceDep1_z3	1	0.4457	0.6863	0.65	0.5161

Table B Estimation Result of the SRUM for Switching Region Sets by DML and Vessel Size of Tuna Purse Seine Fleet in EPO

The MDC Procedure

Conditional Logit Estimates

DML=1 Vessel_Size=1_Small (363-700t)

Parameter Estimates

Parameter	DF	Estimate	Standard Error	t Value	Approx Pr > |t|
LDF_travel_sinceDep1_z4	1	0.4773	0.6864	0.70	0.4868
LDF_travel_sinceDep1_z5	1	0.6139	0.6990	0.88	0.3798
LDF_travel_sinceDep1_z6	1	0.3488	0.6873	0.51	0.6118
LDF_travel_sinceDep1_z7	1	0.3953	0.6763	0.58	0.5589
LDF_travel_sinceDep1_z8	1	0.3130	0.6743	0.46	0.6426
LDF_travel_sinceDep1_z9	1	0.2684	0.6853	0.39	0.6954
LDF_travel_sinceDep1_z10	1	0.1384	0.6782	0.20	0.8383
LDF_travel_sinceDep1_z11	1	0.7921	0.7456	1.06	0.2880
lSKJ_sinceDep1_z1	1	-0.1256	0.1597	-0.79	0.4316
lSKJ_sinceDep1_z2	1	-0.0717	0.1675	-0.43	0.6688
lSKJ_sinceDep1_z3	1	-0.0839	0.1506	-0.56	0.5775
lSKJ_sinceDep1_z4	1	-0.0664	0.1510	-0.44	0.6599
lSKJ_sinceDep1_z5	1	-0.0508	0.1541	-0.33	0.7419
lSKJ_sinceDep1_z6	1	-0.0921	0.1520	-0.61	0.5448
lSKJ_sinceDep1_z7	1	-0.1162	0.1496	-0.78	0.4374
lSKJ_sinceDep1_z8	1	0.008605	0.1501	0.06	0.9543
lSKJ_sinceDep1_z9	1	-0.0776	0.1518	-0.51	0.6092
lSKJ_sinceDep1_z10	1	0.0351	0.1535	0.23	0.8190
lSKJ_sinceDep1_z11	1	-0.0705	0.1686	-0.42	0.6757
lYFT_sinceDep1_z1	1	0.2855	0.1654	1.73	0.0843
lYFT_sinceDep1_z2	1	0.0912	0.1413	0.64	0.5189
lYFT_sinceDep1_z3	1	0.1490	0.1113	1.34	0.1806
lYFT_sinceDep1_z4	1	0.0715	0.1061	0.67	0.5001
lYFT_sinceDep1_z5	1	0.1414	0.1093	1.29	0.1957
lYFT_sinceDep1_z6	1	0.0729	0.1153	0.63	0.5269
lYFT_sinceDep1_z7	1	0.1117	0.1018	1.10	0.2722
lYFT_sinceDep1_z8	1	0.0746	0.1002	0.74	0.4570
lYFT_sinceDep1_z9	1	0.0919	0.1025	0.90	0.3697
lYFT_sinceDep1_z10	1	0.1393	0.1015	1.37	0.1698

Table B Estimation Result of the SRUM for Switching Region Sets by DML and Vessel Size of Tuna Purse Seine Fleet in EPO

The MDC Procedure

Conditional Logit Estimates

DML=1 Vessel_Size=1_Small (363-700t)

Parameter Estimates

Parameter	DF	Estimate	Standard Error	t Value	Approx Pr > |t|
lYFT_sinceDep1_z11	1	-0.0216	0.1199	-0.18	0.8569
lBET_sinceDep1_z1	1	-2.2594	1.4656	-1.54	0.1232
lBET_sinceDep1_z2	1	0.2037	0.1521	1.34	0.1806
lBET_sinceDep1_z3	1	-1.0255	0.2091	-4.91	<.0001
lBET_sinceDep1_z4	1	0.1981	0.1253	1.58	0.1139
lBET_sinceDep1_z5	1	0.1779	0.1272	1.40	0.1619
lBET_sinceDep1_z6	1	-1.2580	0.1534	-8.20	<.0001
lBET_sinceDep1_z7	1	0.1951	0.1231	1.59	0.1128
lBET_sinceDep1_z8	1	0.1316	0.1224	1.07	0.2826
lBET_sinceDep1_z9	1	0.1636	0.1241	1.32	0.1873
lBET_sinceDep1_z10	1	0.0988	0.1230	0.80	0.4220
lBET_sinceDep1_z11	1	0.0954	0.1319	0.72	0.4695
LDF_search_LastRegion_z1	1	0.3557	0.3039	1.17	0.2417
LDF_search_LastRegion_z2	1	0.4630	0.4703	0.98	0.3248
LDF_search_LastRegion_z3	1	0.2066	0.2721	0.76	0.4476
LDF_search_LastRegion_z4	1	0.2422	0.2712	0.89	0.3719
LDF_search_LastRegion_z5	1	0.3666	0.2742	1.34	0.1812
LDF_search_LastRegion_z6	1	0.1372	0.2773	0.49	0.6207
LDF_search_LastRegion_z7	1	0.2708	0.2675	1.01	0.3114
LDF_search_LastRegion_z8	1	0.2012	0.2654	0.76	0.4483
LDF_search_LastRegion_z9	1	0.3115	0.2721	1.14	0.2522
LDF_search_LastRegion_z10	1	0.2358	0.2670	0.88	0.3772
LDF_search_LastRegion_z11	1	0.6788	0.4520	1.50	0.1332
LDF_travel_LastRegion_z1	1	-0.2120	0.3224	-0.66	0.5109
LDF_travel_LastRegion_z2	1	-0.0130	0.3715	-0.04	0.9721
LDF_travel_LastRegion_z3	1	-0.1297	0.3122	-0.42	0.6777
LDF_travel_LastRegion_z4	1	-0.1251	0.3115	-0.40	0.6880
LDF_travel_LastRegion_z5	1	-0.2379	0.3118	-0.76	0.4455
LDF_travel_LastRegion_z6	1	-0.1277	0.3144	-0.41	0.6846

Table B Estimation Result of the SRUM for Switching Region Sets by DML and Vessel Size of Tuna Purse Seine Fleet in EPO

The MDC Procedure

Conditional Logit Estimates

DML=1 Vessel_Size=1_Small (363-700t)

Parameter Estimates

Parameter	DF	Estimate	Standard Error	t Value	Approx Pr > |t|
LDF_travel_LastRegion_z7	1	-0.1222	0.3084	-0.40	0.6918
LDF_travel_LastRegion_z8	1	-0.1581	0.3073	-0.51	0.6069
LDF_travel_LastRegion_z9	1	-0.0950	0.3105	-0.31	0.7598
LDF_travel_LastRegion_z10	1	-0.1175	0.3077	-0.38	0.7025
LDF_travel_LastRegion_z11	1	-0.3377	0.3198	-1.06	0.2909
lDOL_LastRegion_z1	1	2.0340	2.1207	0.96	0.3375
lDOL_LastRegion_z2	1	1.3753	2.0497	0.67	0.5022
lDOL_LastRegion_z3	1	2.1065	2.1192	0.99	0.3202
lDOL_LastRegion_z4	1	1.9345	2.1191	0.91	0.3613
lDOL_LastRegion_z5	1	1.9795	2.1189	0.93	0.3502
lDOL_LastRegion_z6	1	2.2143	2.1217	1.04	0.2967
lDOL_LastRegion_z7	1	1.9313	2.1190	0.91	0.3621
lDOL_LastRegion_z8	1	2.0569	2.1193	0.97	0.3318
lDOL_LastRegion_z9	1	1.9494	2.1196	0.92	0.3577
lDOL_LastRegion_z10	1	1.9149	2.1196	0.90	0.3663
lDOL_LastRegion_z11	1	1.8744	2.1196	0.88	0.3765
lOBJ_LastRegion_z1	1	0.1762	0.3064	0.58	0.5652
lOBJ_LastRegion_z2	1	-0.8732	0.8205	-1.06	0.2872
lOBJ_LastRegion_z3	1	0.1456	0.3034	0.48	0.6313
lOBJ_LastRegion_z4	1	0.1926	0.3027	0.64	0.5247
lOBJ_LastRegion_z5	1	0.1643	0.3053	0.54	0.5905
lOBJ_LastRegion_z6	1	0.1500	0.3034	0.49	0.6209
lOBJ_LastRegion_z7	1	0.1654	0.3004	0.55	0.5819
lOBJ_LastRegion_z8	1	0.2017	0.3001	0.67	0.5015
lOBJ_LastRegion_z9	1	0.1744	0.3012	0.58	0.5625
lOBJ_LastRegion_z10	1	0.1290	0.2999	0.43	0.6670
lOBJ_LastRegion_z11	1	0.2314	0.3074	0.75	0.4517
lNOA_LastRegion_z1	1	0.2847	0.2928	0.97	0.3308
lNOA_LastRegion_z2	1	-0.4504	0.5831	-0.77	0.4399

Table B Estimation Result of the SRUM for Switching Region Sets by DML and Vessel Size of Tuna Purse Seine Fleet in EPO

The MDC Procedure

Conditional Logit Estimates

DML=1 Vessel_Size=1_Small (363-700t)

Parameter Estimates

Parameter	DF	Estimate	Standard Error	t Value	Approx Pr > |t|
lNOA_LastRegion_z3	1	0.3479	0.2896	1.20	0.2296
lNOA_LastRegion_z4	1	0.1937	0.2907	0.67	0.5053
lNOA_LastRegion_z5	1	0.2742	0.2918	0.94	0.3473
lNOA_LastRegion_z6	1	0.4332	0.2895	1.50	0.1346
lNOA_LastRegion_z7	1	0.3056	0.2881	1.06	0.2888
lNOA_LastRegion_z8	1	0.2716	0.2882	0.94	0.3459
lNOA_LastRegion_z9	1	0.3727	0.2888	1.29	0.1969
lNOA_LastRegion_z10	1	0.3178	0.2879	1.10	0.2698
lNOA_LastRegion_z11	1	0.2474	0.2933	0.84	0.3989
lYFT_LastRegion_z1	1	0.1154	0.1579	0.73	0.4649
lYFT_LastRegion_z2	1	0.6892	0.4216	1.63	0.1022
lYFT_LastRegion_z3	1	0.0415	0.1418	0.29	0.7698
lYFT_LastRegion_z4	1	0.2412	0.1400	1.72	0.0849
lYFT_LastRegion_z5	1	0.1114	0.1417	0.79	0.4315
lYFT_LastRegion_z6	1	0.1364	0.1449	0.94	0.3464
lYFT_LastRegion_z7	1	0.2022	0.1356	1.49	0.1359
lYFT_LastRegion_z8	1	0.1517	0.1353	1.12	0.2622
lYFT_LastRegion_z9	1	0.0992	0.1377	0.72	0.4712
lYFT_LastRegion_z10	1	0.1503	0.1348	1.12	0.2647
lYFT_LastRegion_z11	1	0.1688	0.1482	1.14	0.2549
lBET_LastRegion_z1	0	-1.8118	.	.	.
lBET_LastRegion_z2	1	0.0517	0.6046	0.09	0.9319
lBET_LastRegion_z3	0	1.0072	.	.	.
lBET_LastRegion_z4	1	-0.0805	0.1625	-0.50	0.6204
lBET_LastRegion_z5	1	0.0000159	0.1656	0.00	0.9999
lBET_LastRegion_z6	0	1.3795	.	.	.
lBET_LastRegion_z7	1	-0.0293	0.1576	-0.19	0.8527
lBET_LastRegion_z8	1	0.0225	0.1569	0.14	0.8858
lBET_LastRegion_z9	1	-0.0298	0.1602	-0.19	0.8522

Table B Estimation Result of the SRUM for Switching Region Sets by DML and Vessel Size of Tuna Purse Seine Fleet in EPO

The MDC Procedure

Conditional Logit Estimates

DML=1 Vessel_Size=1_Small (363-700t)

Parameter Estimates

Parameter	DF	Estimate	Standard Error	t Value	Approx Pr > |t|
lBET_LastRegion_z10	1	0.0443	0.1570	0.28	0.7777
lBET_LastRegion_z11	1	-0.2204	0.1817	-1.21	0.2251
lSKJ_LastRegion_z1	1	-0.2754	0.2584	-1.07	0.2864
lSKJ_LastRegion_z2	1	-0.3346	0.2701	-1.24	0.2155
lSKJ_LastRegion_z3	1	-0.4091	0.2525	-1.62	0.1051
lSKJ_LastRegion_z4	1	-0.4103	0.2525	-1.62	0.1042
lSKJ_LastRegion_z5	1	-0.3650	0.2542	-1.44	0.1510
lSKJ_LastRegion_z6	1	-0.4387	0.2531	-1.73	0.0831
lSKJ_LastRegion_z7	1	-0.3972	0.2508	-1.58	0.1133
lSKJ_LastRegion_z8	1	-0.4267	0.2505	-1.70	0.0885
lSKJ_LastRegion_z9	1	-0.4244	0.2516	-1.69	0.0917
lSKJ_LastRegion_z10	1	-0.3615	0.2504	-1.44	0.1488
lSKJ_LastRegion_z11	1	-0.2407	0.2602	-0.93	0.3549
SST_DOL_L	1	0.001677	0.001521	1.10	0.2704
SST_DOL_H	1	-0.008820	0.002903	-3.04	0.0024
O2_DOL_L	1	-0.0134	0.0351	-0.38	0.7028
O2_DOL_H	1	-0.0800	0.0224	-3.57	0.0004
SSH_DOL_L	1	-0.002331	0.002342	-1.00	0.3196
SSH_DOL_H	1	0.007505	0.001958	3.83	0.0001
MLD_DOL_L	1	0.003890	0.003565	1.09	0.2753
MLD_DOL_H	1	-0.003398	0.001074	-3.16	0.0016
CHLORO_DOL_L	1	-0.000084	0.002015	-0.04	0.9668
CHLORO_DOL_H	1	0.0000214	0.003850	0.01	0.9956
MEI_z1	1	-0.8869	0.5448	-1.63	0.1035
MEI_z2	1	-0.9675	0.7214	-1.34	0.1799
MEI_z3	1	-1.0922	0.5014	-2.18	0.0294
MEI_z4	1	-0.8917	0.5100	-1.75	0.0803
MEI_z5	1	-0.4990	0.5139	-0.97	0.3315
MEI_z6	1	-0.5618	0.5014	-1.12	0.2625

Table B Estimation Result of the SRUM for Switching Region Sets by DML and Vessel Size of Tuna Purse Seine Fleet in EPO

The MDC Procedure

Conditional Logit Estimates

DML=1 Vessel_Size=1_Small (363-700t)

Parameter Estimates

Parameter	DF	Estimate	Standard Error	t Value	Approx Pr > |t|
MEI_z7	1	-0.7964	0.4982	-1.60	0.1099
MEI_z8	1	-0.5860	0.4904	-1.19	0.2321
MEI_z9	1	-0.2354	0.4969	-0.47	0.6356
MEI_z10	1	-0.1234	0.4903	-0.25	0.8013
MEI_z11	1	0.2251	0.5146	0.44	0.6617

Table B Estimation Result of the SRUM for Switching Region Sets by DML and Vessel Size of Tuna Purse Seine Fleet in EPO

The MDC Procedure

Conditional Logit Estimates

DML=1 Vessel_Size=2_Median (700-1,050t)

Algorithm converged.

Model Fit Summary	
Dependent Variable	Decision
Number of Observations	5332
Number of Cases	63984
Log Likelihood	-10177
Log Likelihood Null (LogL(0))	-13250
Maximum Absolute Gradient	28.58104
Number of Iterations	311
Optimization Method	Dual Quasi-Newton
AIC	20711
Schwarz Criterion	21889

Discrete Response Profile

Index	CHOICE	Frequency	Percent
0	1	285	5.35
1	2	333	6.25
2	3	470	8.81
3	4	694	13.02
4	5	252	4.73
5	6	479	8.98
6	7	1096	20.56
7	8	498	9.34
8	9	564	10.58
9	10	389	7.30
10	11	180	3.38
11	12	92	1.73

Table B Estimation Result of the SRUM for Switching Region Sets by DML and Vessel Size of Tuna Purse Seine Fleet in EPO

The MDC Procedure

Conditional Logit Estimates

DML=1 Vessel_Size=2_Median (700-1,050t)

Goodness-of-Fit Measures		
Measure	Value	Formula
Likelihood Ratio (R)	6145.7	2 * (LogL - LogL0)
Upper Bound of R (U)	26499	- 2 * LogL0
Aldrich-Nelson	0.5354	R / (R+N)
Cragg-Uhler 1	0.6842	1 - exp(-R/N)
Cragg-Uhler 2	0.689	(1-exp(-R/N)) / (1-exp(-U/N))
Estrella	0.7305	1 - (1-R/U)^(U/N)
Adjusted Estrella	0.7062	1 - ((LogL-K)/LogL0)^(-2/N*LogL0)
McFadden's LRI	0.2319	R / U
Veall-Zimmermann	0.6432	(R * (U+N)) / (U * (R+N))
N = # of observations, K = # of regressors		

Table B Estimation Result of the SRUM for Switching Region Sets by DML and Vessel Size of Tuna Purse Seine Fleet in EPO

The MDC Procedure

Conditional Logit Estimates

DML=1 Vessel_Size=2_Median (700-1,050t)

Parameter Estimates

Parameter	DF	Estimate	Standard Error	t Value	Approx Pr > |t|
LDistant_Expected	1	-0.8030	0.0238	-33.72	<.0001
LDistant_Arrive	1	-1.2860	0.0616	-20.87	<.0001
lCPUE_All_1	1	-0.0298	0.0631	-0.47	0.6369
lRPUE_All_1	1	0.0491	0.0411	1.20	0.2315
LDistant_sinceDep1_z1	1	-0.0671	0.1324	-0.51	0.6124
LDistant_sinceDep1_z2	1	-0.5776	0.1288	-4.48	<.0001
LDistant_sinceDep1_z3	1	-2.2018	0.2932	-7.51	<.0001
LDistant_sinceDep1_z4	1	-1.7331	0.2092	-8.29	<.0001
LDistant_sinceDep1_z5	1	-1.3516	0.1785	-7.57	<.0001
LDistant_sinceDep1_z6	1	-2.9815	0.2676	-11.14	<.0001
LDistant_sinceDep1_z7	1	-1.8530	0.2052	-9.03	<.0001
LDistant_sinceDep1_z8	1	-1.8831	0.2155	-8.74	<.0001
LDistant_sinceDep1_z9	1	-2.7127	0.2272	-11.94	<.0001
LDistant_sinceDep1_z10	1	-2.4504	0.2288	-10.71	<.0001
LDistant_sinceDep1_z11	1	-2.0188	0.2399	-8.41	<.0001
LDF_search_sinceDep1_z1	1	-0.0152	0.0191	-0.80	0.4254
LDF_search_sinceDep1_z2	1	0.1899	0.1828	1.04	0.2989
LDF_search_sinceDep1_z3	1	1.1548	0.2313	4.99	<.0001
LDF_search_sinceDep1_z4	1	0.7007	0.1896	3.69	0.0002
LDF_search_sinceDep1_z5	1	0.3238	0.1988	1.63	0.1033
LDF_search_sinceDep1_z6	1	1.6473	0.2249	7.33	<.0001
LDF_search_sinceDep1_z7	1	1.0239	0.1795	5.70	<.0001
LDF_search_sinceDep1_z8	1	0.9448	0.2033	4.65	<.0001
LDF_search_sinceDep1_z9	1	2.4332	0.2239	10.87	<.0001
LDF_search_sinceDep1_z10	1	1.8208	0.2325	7.83	<.0001
LDF_search_sinceDep1_z11	1	0.6980	0.2638	2.65	0.0081
LDF_travel_sinceDep1_z1	1	0.1374	0.0966	1.42	0.1551
LDF_travel_sinceDep1_z2	1	0.2402	0.0905	2.65	0.0079
LDF_travel_sinceDep1_z3	1	0.4376	0.1028	4.26	<.0001

Table B Estimation Result of the SRUM for Switching Region Sets by DML and Vessel Size of Tuna Purse Seine Fleet in EPO

The MDC Procedure

Conditional Logit Estimates

DML=1 Vessel_Size=2_Median (700-1,050t)

Parameter Estimates

Parameter	DF	Estimate	Standard Error	t Value	Approx Pr > |t|
LDF_travel_sinceDep1_z4	1	0.3522	0.0873	4.03	<.0001
LDF_travel_sinceDep1_z5	1	0.3175	0.0938	3.38	0.0007
LDF_travel_sinceDep1_z6	1	0.4018	0.0980	4.10	<.0001
LDF_travel_sinceDep1_z7	1	0.2283	0.0793	2.88	0.0040
LDF_travel_sinceDep1_z8	1	0.2457	0.0859	2.86	0.0042
LDF_travel_sinceDep1_z9	1	0.2537	0.0945	2.68	0.0073
LDF_travel_sinceDep1_z10	1	0.2505	0.0940	2.66	0.0077
LDF_travel_sinceDep1_z11	1	0.3267	0.1069	3.06	0.0022
lSKJ_sinceDep1_z1	1	0.0117	0.0342	0.34	0.7329
lSKJ_sinceDep1_z2	1	0.0386	0.0336	1.15	0.2501
lSKJ_sinceDep1_z3	1	0.0217	0.0335	0.65	0.5167
lSKJ_sinceDep1_z4	1	0.0265	0.0327	0.81	0.4179
lSKJ_sinceDep1_z5	1	0.0760	0.0360	2.11	0.0347
lSKJ_sinceDep1_z6	1	0.007876	0.0334	0.24	0.8138
lSKJ_sinceDep1_z7	1	0.008453	0.0323	0.26	0.7936
lSKJ_sinceDep1_z8	1	0.0461	0.0335	1.38	0.1680
lSKJ_sinceDep1_z9	1	0.0144	0.0331	0.44	0.6628
lSKJ_sinceDep1_z10	1	0.0161	0.0340	0.47	0.6358
lSKJ_sinceDep1_z11	1	0.0688	0.0368	1.87	0.0618
lYFT_sinceDep1_z1	1	0.0519	0.0773	0.67	0.5018
lYFT_sinceDep1_z2	1	-0.0545	0.0595	-0.92	0.3593
lYFT_sinceDep1_z3	1	-0.0102	0.0610	-0.17	0.8669
lYFT_sinceDep1_z4	1	-0.0459	0.0557	-0.82	0.4099
lYFT_sinceDep1_z5	1	-0.0906	0.0568	-1.60	0.1103
lYFT_sinceDep1_z6	1	-0.006418	0.0620	-0.10	0.9176
lYFT_sinceDep1_z7	1	-0.0263	0.0546	-0.48	0.6295
lYFT_sinceDep1_z8	1	-0.0760	0.0551	-1.38	0.1677
lYFT_sinceDep1_z9	1	0.002604	0.0602	0.04	0.9655
lYFT_sinceDep1_z10	1	-0.0235	0.0568	-0.41	0.6783

Table B Estimation Result of the SRUM for Switching Region Sets by DML and Vessel Size of Tuna Purse Seine Fleet in EPO

The MDC Procedure

Conditional Logit Estimates

DML=1 Vessel_Size=2_Median (700-1,050t)

Parameter Estimates

Parameter	DF	Estimate	Standard Error	t Value	Approx Pr > |t|
lYFT_sinceDep1_z11	1	-0.0513	0.0587	-0.87	0.3824
lBET_sinceDep1_z1	1	-0.2657	0.0482	-5.52	<.0001
lBET_sinceDep1_z2	1	-0.1584	0.0330	-4.80	<.0001
lBET_sinceDep1_z3	1	-0.2018	0.0402	-5.02	<.0001
lBET_sinceDep1_z4	1	-0.1040	0.0302	-3.44	0.0006
lBET_sinceDep1_z5	1	-0.0394	0.0312	-1.26	0.2064
lBET_sinceDep1_z6	1	-0.2109	0.0447	-4.72	<.0001
lBET_sinceDep1_z7	1	-0.1169	0.0296	-3.94	<.0001
lBET_sinceDep1_z8	1	-0.0571	0.0298	-1.91	0.0555
lBET_sinceDep1_z9	1	-0.1344	0.0314	-4.28	<.0001
lBET_sinceDep1_z10	1	-0.1475	0.0317	-4.66	<.0001
lBET_sinceDep1_z11	1	-0.1301	0.0356	-3.66	0.0003
LDF_search_LastRegion_z1	1	-0.1036	0.0547	-1.89	0.0582
LDF_search_LastRegion_z2	1	-0.0511	0.0509	-1.00	0.3154
LDF_search_LastRegion_z3	1	-0.1264	0.0513	-2.46	0.0137
LDF_search_LastRegion_z4	1	-0.0473	0.0495	-0.96	0.3390
LDF_search_LastRegion_z5	1	-0.0103	0.0529	-0.19	0.8459
LDF_search_LastRegion_z6	1	-0.1663	0.0522	-3.18	0.0015
LDF_search_LastRegion_z7	1	-0.0402	0.0476	-0.84	0.3983
LDF_search_LastRegion_z8	1	-0.0629	0.0502	-1.25	0.2100
LDF_search_LastRegion_z9	1	-0.0832	0.0504	-1.65	0.0989
LDF_search_LastRegion_z10	1	-0.0172	0.0524	-0.33	0.7426
LDF_search_LastRegion_z11	1	0.0179	0.0588	0.30	0.7616
LDF_travel_LastRegion_z1	1	0.0509	0.0481	1.06	0.2898
LDF_travel_LastRegion_z2	1	0.0251	0.0455	0.55	0.5807
LDF_travel_LastRegion_z3	1	0.1390	0.0472	2.95	0.0032
LDF_travel_LastRegion_z4	1	0.1291	0.0446	2.89	0.0038
LDF_travel_LastRegion_z5	1	0.0634	0.0466	1.36	0.1735
LDF_travel_LastRegion_z6	1	0.1518	0.0470	3.23	0.0012

Table B Estimation Result of the SRUM for Switching Region Sets by DML and Vessel Size of Tuna Purse Seine Fleet in EPO

The MDC Procedure

Conditional Logit Estimates

DML=1 Vessel_Size=2_Median (700-1,050t)

Parameter Estimates

Parameter	DF	Estimate	Standard Error	t Value	Approx Pr > |t|
LDF_travel_LastRegion_z7	1	0.1022	0.0430	2.38	0.0175
LDF_travel_LastRegion_z8	1	0.1459	0.0456	3.20	0.0014
LDF_travel_LastRegion_z9	1	0.1358	0.0455	2.99	0.0028
LDF_travel_LastRegion_z10	1	0.1243	0.0473	2.63	0.0086
LDF_travel_LastRegion_z11	1	0.0674	0.0512	1.32	0.1881
lDOL_LastRegion_z1	1	0.2239	0.0615	3.64	0.0003
lDOL_LastRegion_z2	1	0.0997	0.0510	1.95	0.0508
lDOL_LastRegion_z3	1	0.1895	0.0533	3.56	0.0004
lDOL_LastRegion_z4	1	0.1470	0.0473	3.11	0.0019
lDOL_LastRegion_z5	1	0.1393	0.0487	2.86	0.0043
lDOL_LastRegion_z6	1	0.2894	0.0529	5.47	<.0001
lDOL_LastRegion_z7	1	0.1714	0.0466	3.68	0.0002
lDOL_LastRegion_z8	1	0.1486	0.0473	3.15	0.0017
lDOL_LastRegion_z9	1	0.1772	0.0490	3.62	0.0003
lDOL_LastRegion_z10	1	0.0788	0.0482	1.64	0.1020
lDOL_LastRegion_z11	1	0.0973	0.0511	1.90	0.0571
lOBJ_LastRegion_z1	1	-0.1302	0.0548	-2.38	0.0175
lOBJ_LastRegion_z2	1	-0.1343	0.0553	-2.43	0.0152
lOBJ_LastRegion_z3	1	-0.1637	0.0543	-3.01	0.0026
lOBJ_LastRegion_z4	1	-0.1256	0.0518	-2.43	0.0153
lOBJ_LastRegion_z5	1	-0.0878	0.0546	-1.61	0.1082
lOBJ_LastRegion_z6	1	-0.1204	0.0536	-2.25	0.0247
lOBJ_LastRegion_z7	1	-0.1216	0.0510	-2.38	0.0172
lOBJ_LastRegion_z8	1	-0.1011	0.0519	-1.95	0.0515
lOBJ_LastRegion_z9	1	-0.1247	0.0522	-2.39	0.0168
lOBJ_LastRegion_z10	1	-0.1245	0.0531	-2.34	0.0192
lOBJ_LastRegion_z11	1	-0.1834	0.0558	-3.29	0.0010
lNOA_LastRegion_z1	1	-0.0193	0.0547	-0.35	0.7245
lNOA_LastRegion_z2	1	-0.0757	0.0563	-1.34	0.1788

Table B Estimation Result of the SRUM for Switching Region Sets by DML and Vessel Size of Tuna Purse Seine Fleet in EPO

The MDC Procedure

Conditional Logit Estimates

DML=1 Vessel_Size=2_Median (700-1,050t)

Parameter Estimates

Parameter	DF	Estimate	Standard Error	t Value	Approx Pr > |t|
lNOA_LastRegion_z3	1	0.005395	0.0518	0.10	0.9171
lNOA_LastRegion_z4	1	0.0247	0.0502	0.49	0.6223
lNOA_LastRegion_z5	1	0.0504	0.0520	0.97	0.3325
lNOA_LastRegion_z6	1	0.0689	0.0511	1.35	0.1773
lNOA_LastRegion_z7	1	0.0666	0.0495	1.34	0.1786
lNOA_LastRegion_z8	1	0.0453	0.0502	0.90	0.3670
lNOA_LastRegion_z9	1	0.0678	0.0505	1.34	0.1788
lNOA_LastRegion_z10	1	0.0829	0.0508	1.63	0.1028
lNOA_LastRegion_z11	1	0.1157	0.0530	2.18	0.0291
lYFT_LastRegion_z1	1	-0.0815	0.0518	-1.57	0.1153
lYFT_LastRegion_z2	1	-0.0220	0.0435	-0.50	0.6141
lYFT_LastRegion_z3	1	-0.1219	0.0450	-2.71	0.0068
lYFT_LastRegion_z4	1	-0.0780	0.0402	-1.94	0.0526
lYFT_LastRegion_z5	1	-0.0901	0.0416	-2.17	0.0302
lYFT_LastRegion_z6	1	-0.1439	0.0442	-3.26	0.0011
lYFT_LastRegion_z7	1	-0.1143	0.0395	-2.90	0.0038
lYFT_LastRegion_z8	1	-0.0924	0.0403	-2.30	0.0217
lYFT_LastRegion_z9	1	-0.1199	0.0417	-2.88	0.0040
lYFT_LastRegion_z10	1	-0.1041	0.0412	-2.53	0.0116
lYFT_LastRegion_z11	1	-0.0819	0.0446	-1.84	0.0661
lBET_LastRegion_z1	1	-0.004782	0.0789	-0.06	0.9517
lBET_LastRegion_z2	1	0.0964	0.0436	2.21	0.0271
lBET_LastRegion_z3	1	-0.0218	0.0728	-0.30	0.7648
lBET_LastRegion_z4	1	0.0461	0.0391	1.18	0.2384
lBET_LastRegion_z5	1	0.0569	0.0393	1.45	0.1478
lBET_LastRegion_z6	1	-0.3384	0.1654	-2.05	0.0408
lBET_LastRegion_z7	1	0.0647	0.0383	1.69	0.0918
lBET_LastRegion_z8	1	-0.003784	0.0380	-0.10	0.9206
lBET_LastRegion_z9	1	-0.008772	0.0449	-0.20	0.8451

Table B Estimation Result of the SRUM for Switching Region Sets by DML and Vessel Size of Tuna Purse Seine Fleet in EPO

The MDC Procedure

Conditional Logit Estimates

DML=1 Vessel_Size=2_Median (700-1,050t)

Parameter Estimates

Parameter	DF	Estimate	Standard Error	t Value	Approx Pr > |t|
lBET_LastRegion_z10	1	0.0232	0.0416	0.56	0.5779
lBET_LastRegion_z11	1	-0.0923	0.0599	-1.54	0.1232
lSKJ_LastRegion_z1	1	0.1553	0.0455	3.42	0.0006
lSKJ_LastRegion_z2	1	0.0927	0.0452	2.05	0.0402
lSKJ_LastRegion_z3	1	0.1360	0.0446	3.05	0.0023
lSKJ_LastRegion_z4	1	0.0941	0.0433	2.17	0.0299
lSKJ_LastRegion_z5	1	0.0806	0.0457	1.77	0.0774
lSKJ_LastRegion_z6	1	0.0792	0.0447	1.77	0.0765
lSKJ_LastRegion_z7	1	0.0834	0.0429	1.94	0.0519
lSKJ_LastRegion_z8	1	0.1033	0.0437	2.36	0.0182
lSKJ_LastRegion_z9	1	0.1017	0.0439	2.32	0.0206
lSKJ_LastRegion_z10	1	0.1090	0.0449	2.43	0.0153
lSKJ_LastRegion_z11	1	0.1430	0.0472	3.03	0.0024
SST_DOL_L	1	0.000371	0.000469	0.79	0.4290
SST_DOL_H	1	-0.005377	0.000947	-5.68	<.0001
O2_DOL_L	1	0.0113	0.0152	0.74	0.4570
O2_DOL_H	1	-0.0308	0.003340	-9.22	<.0001
SSH_DOL_L	1	-0.003169	0.000915	-3.47	0.0005
SSH_DOL_H	1	0.001637	0.000554	2.96	0.0031
MLD_DOL_L	1	0.004616	0.001304	3.54	0.0004
MLD_DOL_H	1	-0.002904	0.000362	-8.02	<.0001
CHLORO_DOL_L	1	-0.000326	0.000584	-0.56	0.5766
CHLORO_DOL_H	1	-0.002742	0.001379	-1.99	0.0468
MEI_z1	1	-0.2755	0.1329	-2.07	0.0382
MEI_z2	1	-0.2075	0.1322	-1.57	0.1166
MEI_z3	1	-0.4051	0.1196	-3.39	0.0007
MEI_z4	1	-0.4264	0.1111	-3.84	0.0001
MEI_z5	1	-0.2535	0.1122	-2.26	0.0238
MEI_z6	1	-0.3359	0.1159	-2.90	0.0037

Table B Estimation Result of the SRUM for Switching Region Sets by DML and Vessel Size of Tuna Purse Seine Fleet in EPO

The MDC Procedure

Conditional Logit Estimates

DML=1 Vessel_Size=2_Median (700-1,050t)

Parameter Estimates

Parameter	DF	Estimate	Standard Error	t Value	Approx Pr > |t|
MEI_z7	1	-0.3495	0.1078	-3.24	0.0012
MEI_z8	1	-0.4150	0.1118	-3.71	0.0002
MEI_z9	1	-0.4023	0.1133	-3.55	0.0004
MEI_z10	1	-0.5024	0.1173	-4.28	<.0001
MEI_z11	1	-0.4119	0.1320	-3.12	0.0018

Table B Estimation Result of the SRUM for Switching Region Sets by DML and Vessel Size of Tuna Purse Seine Fleet in EPO

The MDC Procedure

Conditional Logit Estimates

DML=1 Vessel_Size=3_Large (1,050-1,250t)

Algorithm converged.

Model Fit Summary	
Dependent Variable	Decision
Number of Observations	8616
Number of Cases	103392
Log Likelihood	-16338
Log Likelihood Null (LogL(0))	-21410
Maximum Absolute Gradient	15.16889
Number of Iterations	316
Optimization Method	Dual Quasi-Newton
AIC	33034
Schwarz Criterion	34298

Discrete Response Profile

Index	CHOICE	Frequency	Percent
0	1	566	6.57
1	2	532	6.17
2	3	977	11.34
3	4	1134	13.16
4	5	384	4.46
5	6	924	10.72
6	7	1677	19.46
7	8	838	9.73
8	9	738	8.57
9	10	532	6.17
10	11	225	2.61
11	12	89	1.03

Table B Estimation Result of the SRUM for Switching Region Sets by DML and Vessel Size of Tuna Purse Seine Fleet in EPO

The MDC Procedure

Conditional Logit Estimates

DML=1 Vessel_Size=3_Large (1,050-1,250t)

Goodness-of-Fit Measures		
Measure	Value	Formula
Likelihood Ratio (R)	10144	2 * (LogL - LogL0)
Upper Bound of R (U)	42820	- 2 * LogL0
Aldrich-Nelson	0.5407	R / (R+N)
Cragg-Uhler 1	0.6919	1 - exp(-R/N)
Cragg-Uhler 2	0.6967	(1-exp(-R/N)) / (1-exp(-U/N))
Estrella	0.7391	1 - (1-R/U)^(U/N)
Adjusted Estrella	0.7246	1 - ((LogL-K)/LogL0)^(-2/N*LogL0)
McFadden's LRI	0.2369	R / U
Veall-Zimmermann	0.6495	(R * (U+N)) / (U * (R+N))
N = # of observations, K = # of regressors		

Table B Estimation Result of the SRUM for Switching Region Sets by DML and Vessel Size of Tuna Purse Seine Fleet in EPO

The MDC Procedure

Conditional Logit Estimates

DML=1 Vessel_Size=3_Large (1,050-1,250t)

Parameter Estimates

Parameter	DF	Estimate	Standard Error	t Value	Approx Pr > |t|
LDistant_Expected	1	-0.8015	0.0190	-42.11	<.0001
LDistant_Arrive	1	-1.3011	0.0396	-32.86	<.0001
lCPUE_All_1	1	0.0991	0.0516	1.92	0.0546
lRPUE_All_1	1	-0.0295	0.0334	-0.88	0.3774
LDistant_sinceDep1_z1	1	0.1698	0.1403	1.21	0.2262
LDistant_sinceDep1_z2	1	-0.3848	0.1418	-2.71	0.0066
LDistant_sinceDep1_z3	1	-2.0156	0.2294	-8.79	<.0001
LDistant_sinceDep1_z4	1	-1.5164	0.1871	-8.10	<.0001
LDistant_sinceDep1_z5	1	-1.3535	0.1751	-7.73	<.0001
LDistant_sinceDep1_z6	1	-2.3194	0.2280	-10.17	<.0001
LDistant_sinceDep1_z7	1	-1.6933	0.1867	-9.07	<.0001
LDistant_sinceDep1_z8	1	-1.7750	0.1927	-9.21	<.0001
LDistant_sinceDep1_z9	1	-2.4739	0.2020	-12.24	<.0001
LDistant_sinceDep1_z10	1	-2.2191	0.2033	-10.92	<.0001
LDistant_sinceDep1_z11	1	-1.9051	0.2071	-9.20	<.0001
LDF_search_sinceDep1_z1	1	0.002055	0.0293	0.07	0.9441
LDF_search_sinceDep1_z2	1	0.3845	0.1684	2.28	0.0224
LDF_search_sinceDep1_z3	1	1.9177	0.1862	10.30	<.0001
LDF_search_sinceDep1_z4	1	0.9777	0.1717	5.70	<.0001
LDF_search_sinceDep1_z5	1	1.1707	0.2113	5.54	<.0001
LDF_search_sinceDep1_z6	1	1.8471	0.2088	8.85	<.0001
LDF_search_sinceDep1_z7	1	1.4274	0.2029	7.04	<.0001
LDF_search_sinceDep1_z8	1	1.5677	0.2331	6.73	<.0001
LDF_search_sinceDep1_z9	1	2.7506	0.2634	10.44	<.0001
LDF_search_sinceDep1_z10	1	2.0834	0.2869	7.26	<.0001
LDF_search_sinceDep1_z11	1	1.4307	0.3314	4.32	<.0001
LDF_travel_sinceDep1_z1	1	-0.0934	0.1324	-0.71	0.4805
LDF_travel_sinceDep1_z2	1	-0.001103	0.1307	-0.01	0.9933
LDF_travel_sinceDep1_z3	1	0.1205	0.1351	0.89	0.3725

Table B Estimation Result of the SRUM for Switching Region Sets by DML and Vessel Size of Tuna Purse Seine Fleet in EPO

The MDC Procedure

Conditional Logit Estimates

DML=1 Vessel_Size=3_Large (1,050-1,250t)

Parameter Estimates

Parameter	DF	Estimate	Standard Error	t Value	Approx Pr > |t|
LDF_travel_sinceDep1_z4	1	0.1287	0.1284	1.00	0.3162
LDF_travel_sinceDep1_z5	1	-0.008065	0.1348	-0.06	0.9523
LDF_travel_sinceDep1_z6	1	0.0336	0.1293	0.26	0.7950
LDF_travel_sinceDep1_z7	1	-0.0139	0.1244	-0.11	0.9108
LDF_travel_sinceDep1_z8	1	-0.0374	0.1273	-0.29	0.7687
LDF_travel_sinceDep1_z9	1	-0.0390	0.1298	-0.30	0.7638
LDF_travel_sinceDep1_z10	1	-0.0832	0.1304	-0.64	0.5234
LDF_travel_sinceDep1_z11	1	0.0684	0.1452	0.47	0.6375
lSKJ_sinceDep1_z1	1	-0.0200	0.0326	-0.61	0.5389
lSKJ_sinceDep1_z2	1	-0.0262	0.0325	-0.81	0.4204
lSKJ_sinceDep1_z3	1	-0.0271	0.0321	-0.85	0.3978
lSKJ_sinceDep1_z4	1	-0.0257	0.0318	-0.81	0.4194
lSKJ_sinceDep1_z5	1	-0.0144	0.0336	-0.43	0.6677
lSKJ_sinceDep1_z6	1	-0.0641	0.0321	-1.99	0.0461
lSKJ_sinceDep1_z7	1	-0.0629	0.0316	-1.99	0.0462
lSKJ_sinceDep1_z8	1	-0.0241	0.0321	-0.75	0.4526
lSKJ_sinceDep1_z9	1	-0.0738	0.0323	-2.29	0.0222
lSKJ_sinceDep1_z10	1	-0.0388	0.0328	-1.18	0.2373
lSKJ_sinceDep1_z11	1	0.0117	0.0351	0.33	0.7393
lYFT_sinceDep1_z1	1	0.2565	0.0614	4.18	<.0001
lYFT_sinceDep1_z2	1	0.0777	0.0428	1.82	0.0694
lYFT_sinceDep1_z3	1	0.1014	0.0458	2.21	0.0270
lYFT_sinceDep1_z4	1	0.0750	0.0400	1.88	0.0605
lYFT_sinceDep1_z5	1	0.0107	0.0403	0.27	0.7904
lYFT_sinceDep1_z6	1	0.0796	0.0420	1.90	0.0580
lYFT_sinceDep1_z7	1	0.0839	0.0386	2.17	0.0299
lYFT_sinceDep1_z8	1	0.0455	0.0391	1.16	0.2441
lYFT_sinceDep1_z9	1	0.0936	0.0426	2.20	0.0278
lYFT_sinceDep1_z10	1	0.0904	0.0413	2.19	0.0288

Table B Estimation Result of the SRUM for Switching Region Sets by DML and Vessel Size of Tuna Purse Seine Fleet in EPO

The MDC Procedure

Conditional Logit Estimates

DML=1 Vessel_Size=3_Large (1,050-1,250t)

Parameter Estimates

Parameter	DF	Estimate	Standard Error	t Value	Approx Pr > |t|
lYFT_sinceDep1_z11	1	0.0303	0.0434	0.70	0.4853
lBET_sinceDep1_z1	1	-0.2083	0.0341	-6.10	<.0001
lBET_sinceDep1_z2	1	-0.1128	0.0282	-4.01	<.0001
lBET_sinceDep1_z3	1	-0.1499	0.0300	-5.00	<.0001
lBET_sinceDep1_z4	1	-0.0725	0.0263	-2.76	0.0058
lBET_sinceDep1_z5	1	-0.0363	0.0273	-1.33	0.1828
lBET_sinceDep1_z6	1	-0.1644	0.0320	-5.14	<.0001
lBET_sinceDep1_z7	1	-0.0791	0.0257	-3.07	0.0021
lBET_sinceDep1_z8	1	-0.0594	0.0259	-2.29	0.0220
lBET_sinceDep1_z9	1	-0.1022	0.0270	-3.78	0.0002
lBET_sinceDep1_z10	1	-0.0984	0.0272	-3.62	0.0003
lBET_sinceDep1_z11	1	-0.0855	0.0296	-2.89	0.0039
LDF_search_LastRegion_z1	1	-0.0460	0.0510	-0.90	0.3669
LDF_search_LastRegion_z2	1	-0.0152	0.0494	-0.31	0.7588
LDF_search_LastRegion_z3	1	-0.0758	0.0489	-1.55	0.1212
LDF_search_LastRegion_z4	1	-0.0389	0.0481	-0.81	0.4177
LDF_search_LastRegion_z5	1	-0.006196	0.0517	-0.12	0.9046
LDF_search_LastRegion_z6	1	-0.1036	0.0493	-2.10	0.0357
LDF_search_LastRegion_z7	1	-0.0255	0.0470	-0.54	0.5864
LDF_search_LastRegion_z8	1	0.0170	0.0485	0.35	0.7258
LDF_search_LastRegion_z9	1	-0.0853	0.0494	-1.73	0.0841
LDF_search_LastRegion_z10	1	-0.0323	0.0507	-0.64	0.5237
LDF_search_LastRegion_z11	1	0.0173	0.0565	0.31	0.7602
LDF_travel_LastRegion_z1	1	0.1016	0.0446	2.28	0.0227
LDF_travel_LastRegion_z2	1	0.0453	0.0437	1.04	0.3002
LDF_travel_LastRegion_z3	1	0.1364	0.0437	3.12	0.0018
LDF_travel_LastRegion_z4	1	0.1114	0.0428	2.61	0.0092
LDF_travel_LastRegion_z5	1	0.1045	0.0460	2.27	0.0231
LDF_travel_LastRegion_z6	1	0.1341	0.0437	3.07	0.0022

Table B Estimation Result of the SRUM for Switching Region Sets by DML and Vessel Size of Tuna Purse Seine Fleet in EPO

The MDC Procedure

Conditional Logit Estimates

DML=1 Vessel_Size=3_Large (1,050-1,250t)

Parameter Estimates

Parameter	DF	Estimate	Standard Error	t Value	Approx Pr > |t|
LDF_travel_LastRegion_z7	1	0.0988	0.0419	2.36	0.0185
LDF_travel_LastRegion_z8	1	0.0895	0.0429	2.09	0.0368
LDF_travel_LastRegion_z9	1	0.1503	0.0444	3.39	0.0007
LDF_travel_LastRegion_z10	1	0.1320	0.0455	2.90	0.0037
LDF_travel_LastRegion_z11	1	0.0912	0.0491	1.86	0.0632
lDOL_LastRegion_z1	1	0.2883	0.0531	5.43	<.0001
lDOL_LastRegion_z2	1	0.1876	0.0491	3.82	0.0001
lDOL_LastRegion_z3	1	0.2245	0.0485	4.63	<.0001
lDOL_LastRegion_z4	1	0.2170	0.0452	4.80	<.0001
lDOL_LastRegion_z5	1	0.1900	0.0469	4.05	<.0001
lDOL_LastRegion_z6	1	0.3191	0.0479	6.67	<.0001
lDOL_LastRegion_z7	1	0.2344	0.0447	5.25	<.0001
lDOL_LastRegion_z8	1	0.2000	0.0447	4.47	<.0001
lDOL_LastRegion_z9	1	0.1970	0.0460	4.28	<.0001
lDOL_LastRegion_z10	1	0.1659	0.0456	3.64	0.0003
lDOL_LastRegion_z11	1	0.0969	0.0479	2.02	0.0434
lOBJ_LastRegion_z1	1	0.001106	0.0520	0.02	0.9830
lOBJ_LastRegion_z2	1	-0.0297	0.0529	-0.56	0.5745
lOBJ_LastRegion_z3	1	-0.0199	0.0516	-0.39	0.6992
lOBJ_LastRegion_z4	1	-0.0346	0.0508	-0.68	0.4962
lOBJ_LastRegion_z5	1	-0.001744	0.0529	-0.03	0.9737
lOBJ_LastRegion_z6	1	-0.0250	0.0515	-0.49	0.6269
lOBJ_LastRegion_z7	1	-0.0121	0.0504	-0.24	0.8103
lOBJ_LastRegion_z8	1	0.0464	0.0507	0.92	0.3598
lOBJ_LastRegion_z9	1	0.0254	0.0513	0.49	0.6210
lOBJ_LastRegion_z10	1	0.0687	0.0516	1.33	0.1829
lOBJ_LastRegion_z11	1	-0.0225	0.0543	-0.41	0.6782
lNOA_LastRegion_z1	1	0.0532	0.0480	1.11	0.2674
lNOA_LastRegion_z2	1	-0.0220	0.0500	-0.44	0.6599

Table B Estimation Result of the SRUM for Switching Region Sets by DML and Vessel Size of Tuna Purse Seine Fleet in EPO

The MDC Procedure

Conditional Logit Estimates

DML=1 Vessel_Size=3_Large (1,050-1,250t)

Parameter Estimates

Parameter	DF	Estimate	Standard Error	t Value	Approx Pr > |t|
lNOA_LastRegion_z3	1	0.0466	0.0467	1.00	0.3189
lNOA_LastRegion_z4	1	0.0855	0.0460	1.86	0.0632
lNOA_LastRegion_z5	1	0.0672	0.0477	1.41	0.1592
lNOA_LastRegion_z6	1	0.1293	0.0462	2.80	0.0051
lNOA_LastRegion_z7	1	0.1030	0.0457	2.25	0.0242
lNOA_LastRegion_z8	1	0.0953	0.0460	2.07	0.0381
lNOA_LastRegion_z9	1	0.1336	0.0464	2.88	0.0040
lNOA_LastRegion_z10	1	0.1382	0.0467	2.96	0.0031
lNOA_LastRegion_z11	1	0.1030	0.0488	2.11	0.0347
lYFT_LastRegion_z1	1	-0.1967	0.0457	-4.31	<.0001
lYFT_LastRegion_z2	1	-0.1267	0.0428	-2.96	0.0031
lYFT_LastRegion_z3	1	-0.1814	0.0422	-4.30	<.0001
lYFT_LastRegion_z4	1	-0.1510	0.0397	-3.80	0.0001
lYFT_LastRegion_z5	1	-0.1732	0.0410	-4.22	<.0001
lYFT_LastRegion_z6	1	-0.2120	0.0417	-5.08	<.0001
lYFT_LastRegion_z7	1	-0.1895	0.0391	-4.84	<.0001
lYFT_LastRegion_z8	1	-0.1691	0.0393	-4.31	<.0001
lYFT_LastRegion_z9	1	-0.1638	0.0404	-4.05	<.0001
lYFT_LastRegion_z10	1	-0.1661	0.0402	-4.13	<.0001
lYFT_LastRegion_z11	1	-0.0960	0.0426	-2.25	0.0244
lBET_LastRegion_z1	1	0.0849	0.0532	1.59	0.1109
lBET_LastRegion_z2	1	0.1127	0.0413	2.73	0.0063
lBET_LastRegion_z3	1	0.1043	0.0465	2.24	0.0249
lBET_LastRegion_z4	1	0.1087	0.0373	2.92	0.0035
lBET_LastRegion_z5	1	0.0951	0.0383	2.49	0.0129
lBET_LastRegion_z6	1	0.0409	0.0552	0.74	0.4588
lBET_LastRegion_z7	1	0.1054	0.0366	2.88	0.0040
lBET_LastRegion_z8	1	0.0686	0.0361	1.90	0.0575
lBET_LastRegion_z9	1	0.0489	0.0397	1.23	0.2186

Table B Estimation Result of the SRUM for Switching Region Sets by DML and Vessel Size of Tuna Purse Seine Fleet in EPO

The MDC Procedure

Conditional Logit Estimates

DML=1 Vessel_Size=3_Large (1,050-1,250t)

Parameter Estimates

Parameter	DF	Estimate	Standard Error	t Value	Approx Pr > |t|
lBET_LastRegion_z10	1	0.0351	0.0387	0.91	0.3650
lBET_LastRegion_z11	1	0.0292	0.0434	0.67	0.5022
lSKJ_LastRegion_z1	1	0.0175	0.0382	0.46	0.6468
lSKJ_LastRegion_z2	1	0.002441	0.0384	0.06	0.9493
lSKJ_LastRegion_z3	1	-0.0170	0.0377	-0.45	0.6516
lSKJ_LastRegion_z4	1	-0.001359	0.0372	-0.04	0.9708
lSKJ_LastRegion_z5	1	0.009983	0.0390	0.26	0.7979
lSKJ_LastRegion_z6	1	0.005297	0.0377	0.14	0.8884
lSKJ_LastRegion_z7	1	-0.0194	0.0370	-0.52	0.6002
lSKJ_LastRegion_z8	1	-0.0306	0.0374	-0.82	0.4130
lSKJ_LastRegion_z9	1	-0.0296	0.0380	-0.78	0.4357
lSKJ_LastRegion_z10	1	-0.0417	0.0385	-1.08	0.2790
lSKJ_LastRegion_z11	1	-0.009568	0.0406	-0.24	0.8138
SST_DOL_L	1	0.000192	0.000366	0.53	0.5995
SST_DOL_H	1	-0.005683	0.000736	-7.72	<.0001
O2_DOL_L	1	0.002797	0.0104	0.27	0.7882
O2_DOL_H	1	-0.0301	0.002475	-12.16	<.0001
SSH_DOL_L	1	-0.003515	0.000719	-4.89	<.0001
SSH_DOL_H	1	0.002312	0.000442	5.24	<.0001
MLD_DOL_L	1	0.000850	0.001030	0.83	0.4090
MLD_DOL_H	1	-0.002689	0.000267	-10.08	<.0001
CHLORO_DOL_L	1	0.001075	0.000437	2.46	0.0140
CHLORO_DOL_H	1	0.0000692	0.001081	0.06	0.9489
MEI_z1	1	-0.1878	0.1194	-1.57	0.1156
MEI_z2	1	-0.1456	0.1233	-1.18	0.2378
MEI_z3	1	-0.4248	0.1120	-3.79	0.0001
MEI_z4	1	-0.3800	0.1091	-3.48	0.0005
MEI_z5	1	-0.2651	0.1145	-2.32	0.0206
MEI_z6	1	-0.2847	0.1106	-2.57	0.0101

Table B Estimation Result of the SRUM for Switching Region Sets by DML and Vessel Size of Tuna Purse Seine Fleet in EPO

The MDC Procedure

Conditional Logit Estimates

DML=1 Vessel_Size=3_Large (1,050-1,250t)

Parameter Estimates

Parameter	DF	Estimate	Standard Error	t Value	Approx Pr > |t|
MEI_z7	1	-0.3184	0.1071	-2.97	0.0029
MEI_z8	1	-0.3578	0.1095	-3.27	0.0011
MEI_z9	1	-0.4538	0.1125	-4.04	<.0001
MEI_z10	1	-0.4878	0.1135	-4.30	<.0001
MEI_z11	1	-0.3760	0.1232	-3.05	0.0023

Table B Estimation Result of the SRUM for Switching Region Sets by DML and Vessel Size of Tuna Purse Seine Fleet in EPO

The MDC Procedure

Conditional Logit Estimates

DML=1 Vessel_Size=4_XLarge (1,250-1,800t)

Algorithm converged.

Model Fit Summary	
Dependent Variable	Decision
Number of Observations	2385
Number of Cases	28620
Log Likelihood	-4680
Log Likelihood Null (LogL(0))	-5927
Maximum Absolute Gradient	24.56521
Number of Iterations	315
Optimization Method	Dual Quasi-Newton
AIC	9718
Schwarz Criterion	10752

Discrete Response Profile

Index	CHOICE	Frequency	Percent
0	1	89	3.73
1	2	207	8.68
2	3	93	3.90
3	4	325	13.63
4	5	192	8.05
5	6	93	3.90
6	7	449	18.83
7	8	309	12.96
8	9	213	8.93
9	10	204	8.55
10	11	106	4.44
11	12	105	4.40

Table B Estimation Result of the SRUM for Switching Region Sets by DML and Vessel Size of Tuna Purse Seine Fleet in EPO

The MDC Procedure

Conditional Logit Estimates

DML=1 Vessel_Size=4_XLarge (1,250-1,800t)

Goodness-of-Fit Measures

Measure	Value	Formula
Likelihood Ratio (R)	2493.3	2 * (LogL - LogL0)
Upper Bound of R (U)	11853	- 2 * LogL0
Aldrich-Nelson	0.5111	R / (R+N)
Cragg-Uhler 1	0.6485	1 - exp(-R/N)
Cragg-Uhler 2	0.653	(1-exp(-R/N)) / (1-exp(-U/N))
Estrella	0.6908	1 - (1-R/U)^(U/N)
Adjusted Estrella	0.6274	1 - ((LogL-K)/LogL0)^(-2/N*LogL0)
McFadden's LRI	0.2104	R / U
Veall-Zimmermann	0.6139	(R * (U+N)) / (U * (R+N))

N = # of observations, K = # of regressors

Table B Estimation Result of the SRUM for Switching Region Sets by DML and Vessel Size of Tuna Purse Seine Fleet in EPO

The MDC Procedure

Conditional Logit Estimates

DML=1 Vessel_Size=4_XLarge (1,250-1,800t)

Parameter Estimates

Parameter	DF	Estimate	Standard Error	t Value	Approx Pr > |t|
LDistant_Expected	1	-0.6991	0.0353	-19.80	<.0001
LDistant_Arrive	1	-1.0974	0.1012	-10.85	<.0001
lCPUE_All_1	1	0.2993	0.0940	3.18	0.0015
lRPUE_All_1	1	-0.1606	0.0605	-2.65	0.0080
LDistant_sinceDep1_z1	1	0.5239	0.2056	2.55	0.0108
LDistant_sinceDep1_z2	1	0.1164	0.1913	0.61	0.5427
LDistant_sinceDep1_z3	1	-0.6124	0.4424	-1.38	0.1662
LDistant_sinceDep1_z4	1	-0.8362	0.2778	-3.01	0.0026
LDistant_sinceDep1_z5	1	-0.8394	0.2431	-3.45	0.0006
LDistant_sinceDep1_z6	1	-1.2427	0.4019	-3.09	0.0020
LDistant_sinceDep1_z7	1	-1.1527	0.2728	-4.23	<.0001
LDistant_sinceDep1_z8	1	-1.0878	0.2834	-3.84	0.0001
LDistant_sinceDep1_z9	1	-1.8612	0.3012	-6.18	<.0001
LDistant_sinceDep1_z10	1	-1.9362	0.3051	-6.35	<.0001
LDistant_sinceDep1_z11	1	-1.1315	0.3177	-3.56	0.0004
LDF_search_sinceDep1_z1	1	0.0409	0.0379	1.08	0.2804
LDF_search_sinceDep1_z2	1	0.2182	0.3334	0.65	0.5128
LDF_search_sinceDep1_z3	1	1.2545	0.4306	2.91	0.0036
LDF_search_sinceDep1_z4	1	0.6435	0.3024	2.13	0.0334
LDF_search_sinceDep1_z5	1	0.7133	0.3174	2.25	0.0246
LDF_search_sinceDep1_z6	1	1.0774	0.3984	2.70	0.0069
LDF_search_sinceDep1_z7	1	1.1397	0.2796	4.08	<.0001
LDF_search_sinceDep1_z8	1	1.1020	0.2950	3.74	0.0002
LDF_search_sinceDep1_z9	1	2.2780	0.3416	6.67	<.0001
LDF_search_sinceDep1_z10	1	2.5805	0.3627	7.12	<.0001
LDF_search_sinceDep1_z11	1	1.1611	0.3995	2.91	0.0037
LDF_travel_sinceDep1_z1	1	0.2857	0.1779	1.61	0.1082
LDF_travel_sinceDep1_z2	1	0.1833	0.1373	1.34	0.1818
LDF_travel_sinceDep1_z3	1	0.0817	0.1653	0.49	0.6210

Table B Estimation Result of the SRUM for Switching Region Sets by DML and Vessel Size of Tuna Purse Seine Fleet in EPO

The MDC Procedure

Conditional Logit Estimates

DML=1 Vessel_Size=4_XLarge (1,250-1,800t)

Parameter Estimates

Parameter	DF	Estimate	Standard Error	t Value	Approx Pr > |t|
LDF_travel_sinceDep1_z4	1	0.2238	0.1275	1.76	0.0791
LDF_travel_sinceDep1_z5	1	0.1538	0.1366	1.13	0.2603
LDF_travel_sinceDep1_z6	1	0.0427	0.1354	0.32	0.7526
LDF_travel_sinceDep1_z7	1	0.2227	0.1199	1.86	0.0632
LDF_travel_sinceDep1_z8	1	0.0668	0.1179	0.57	0.5710
LDF_travel_sinceDep1_z9	1	0.2020	0.1336	1.51	0.1303
LDF_travel_sinceDep1_z10	1	0.0380	0.1368	0.28	0.7813
LDF_travel_sinceDep1_z11	1	-0.0313	0.1403	-0.22	0.8235
lSKJ_sinceDep1_z1	1	-0.0334	0.0487	-0.69	0.4927
lSKJ_sinceDep1_z2	1	-0.007322	0.0455	-0.16	0.8721
lSKJ_sinceDep1_z3	1	-0.0659	0.0479	-1.37	0.1692
lSKJ_sinceDep1_z4	1	-0.0202	0.0428	-0.47	0.6374
lSKJ_sinceDep1_z5	1	-0.0109	0.0462	-0.23	0.8142
lSKJ_sinceDep1_z6	1	-0.0812	0.0473	-1.72	0.0856
lSKJ_sinceDep1_z7	1	-0.0769	0.0419	-1.83	0.0666
lSKJ_sinceDep1_z8	1	-0.0279	0.0429	-0.65	0.5159
lSKJ_sinceDep1_z9	1	-0.0972	0.0438	-2.22	0.0263
lSKJ_sinceDep1_z10	1	-0.0955	0.0449	-2.13	0.0333
lSKJ_sinceDep1_z11	1	0.0186	0.0488	0.38	0.7025
lYFT_sinceDep1_z1	1	0.0519	0.0703	0.74	0.4598
lYFT_sinceDep1_z2	1	0.001883	0.0438	0.04	0.9657
lYFT_sinceDep1_z3	1	0.0512	0.0713	0.72	0.4731
lYFT_sinceDep1_z4	1	0.0234	0.0411	0.57	0.5683
lYFT_sinceDep1_z5	1	0.002995	0.0431	0.07	0.9446
lYFT_sinceDep1_z6	1	0.2609	0.1109	2.35	0.0187
lYFT_sinceDep1_z7	1	0.0318	0.0408	0.78	0.4351
lYFT_sinceDep1_z8	1	0.0133	0.0407	0.33	0.7433
lYFT_sinceDep1_z9	1	0.1099	0.0544	2.02	0.0433
lYFT_sinceDep1_z10	1	0.002317	0.0454	0.05	0.9593

Table B Estimation Result of the SRUM for Switching Region Sets by DML and Vessel Size of Tuna Purse Seine Fleet in EPO

The MDC Procedure

Conditional Logit Estimates

DML=1 Vessel_Size=4_XLarge (1,250-1,800t)

Parameter Estimates

Parameter	DF	Estimate	Standard Error	t Value	Approx Pr > |t|
lYFT_sinceDep1_z11	1	0.0282	0.0487	0.58	0.5633
lBET_sinceDep1_z1	1	-0.1520	0.0410	-3.71	0.0002
lBET_sinceDep1_z2	1	-0.0764	0.0312	-2.44	0.0145
lBET_sinceDep1_z3	1	-0.1024	0.0384	-2.67	0.0077
lBET_sinceDep1_z4	1	-0.0767	0.0299	-2.57	0.0103
lBET_sinceDep1_z5	1	-0.0218	0.0319	-0.68	0.4953
lBET_sinceDep1_z6	1	-0.1693	0.0527	-3.21	0.0013
lBET_sinceDep1_z7	1	-0.0982	0.0297	-3.31	0.0009
lBET_sinceDep1_z8	1	-0.0874	0.0297	-2.94	0.0033
lBET_sinceDep1_z9	1	-0.1558	0.0339	-4.60	<.0001
lBET_sinceDep1_z10	1	-0.1169	0.0318	-3.68	0.0002
lBET_sinceDep1_z11	1	-0.1515	0.0357	-4.24	<.0001
LDF_search_LastRegion_z1	1	-0.0810	0.0669	-1.21	0.2258
LDF_search_LastRegion_z2	1	-0.0197	0.0539	-0.37	0.7143
LDF_search_LastRegion_z3	1	-0.0771	0.0674	-1.14	0.2523
LDF_search_LastRegion_z4	1	0.0123	0.0521	0.24	0.8131
LDF_search_LastRegion_z5	1	0.0497	0.0547	0.91	0.3637
LDF_search_LastRegion_z6	1	-0.003236	0.0685	-0.05	0.9623
LDF_search_LastRegion_z7	1	0.0286	0.0502	0.57	0.5686
LDF_search_LastRegion_z8	1	0.009073	0.0500	0.18	0.8561
LDF_search_LastRegion_z9	1	-0.0424	0.0555	-0.76	0.4455
LDF_search_LastRegion_z10	1	0.0113	0.0597	0.19	0.8505
LDF_search_LastRegion_z11	1	0.1281	0.0735	1.74	0.0815
LDF_travel_LastRegion_z1	1	-0.0233	0.0631	-0.37	0.7116
LDF_travel_LastRegion_z2	1	-0.0183	0.0546	-0.33	0.7376
LDF_travel_LastRegion_z3	1	0.0645	0.0668	0.97	0.3337
LDF_travel_LastRegion_z4	1	0.0517	0.0524	0.99	0.3242
LDF_travel_LastRegion_z5	1	-0.003095	0.0540	-0.06	0.9543
LDF_travel_LastRegion_z6	1	0.0141	0.0614	0.23	0.8189

Table B Estimation Result of the SRUM for Switching Region Sets by DML and Vessel Size of Tuna Purse Seine Fleet in EPO

The MDC Procedure

Conditional Logit Estimates

DML=1 Vessel_Size=4_XLarge (1,250-1,800t)

Parameter Estimates

Parameter	DF	Estimate	Standard Error	t Value	Approx Pr > |t|
LDF_travel_LastRegion_z7	1	-0.007875	0.0501	-0.16	0.8752
LDF_travel_LastRegion_z8	1	-0.0159	0.0509	-0.31	0.7550
LDF_travel_LastRegion_z9	1	0.0238	0.0552	0.43	0.6666
LDF_travel_LastRegion_z10	1	0.0868	0.0591	1.47	0.1424
LDF_travel_LastRegion_z11	1	0.0486	0.0640	0.76	0.4483
lDOL_LastRegion_z1	1	0.2074	0.0662	3.13	0.0017
lDOL_LastRegion_z2	1	0.1563	0.0527	2.96	0.0030
lDOL_LastRegion_z3	1	0.3058	0.0804	3.80	0.0001
lDOL_LastRegion_z4	1	0.1613	0.0503	3.21	0.0013
lDOL_LastRegion_z5	1	0.1446	0.0523	2.77	0.0057
lDOL_LastRegion_z6	1	0.2593	0.0747	3.47	0.0005
lDOL_LastRegion_z7	1	0.1930	0.0501	3.85	0.0001
lDOL_LastRegion_z8	1	0.1584	0.0498	3.18	0.0015
lDOL_LastRegion_z9	1	0.1548	0.0540	2.87	0.0041
lDOL_LastRegion_z10	1	0.0539	0.0526	1.02	0.3057
lDOL_LastRegion_z11	1	0.0921	0.0569	1.62	0.1054
lOBJ_LastRegion_z1	1	0.0283	0.0634	0.45	0.6557
lOBJ_LastRegion_z2	1	0.002390	0.0586	0.04	0.9675
lOBJ_LastRegion_z3	1	-0.0573	0.0652	-0.88	0.3796
lOBJ_LastRegion_z4	1	-0.0136	0.0549	-0.25	0.8039
lOBJ_LastRegion_z5	1	-0.0107	0.0584	-0.18	0.8552
lOBJ_LastRegion_z6	1	-0.0488	0.0673	-0.72	0.4685
lOBJ_LastRegion_z7	1	-0.0219	0.0539	-0.41	0.6852
lOBJ_LastRegion_z8	1	0.0443	0.0543	0.82	0.4144
lOBJ_LastRegion_z9	1	0.0564	0.0568	0.99	0.3201
lOBJ_LastRegion_z10	1	-0.0306	0.0566	-0.54	0.5881
lOBJ_LastRegion_z11	1	-0.0416	0.0611	-0.68	0.4958
lNOA_LastRegion_z1	1	0.008271	0.0588	0.14	0.8881
lNOA_LastRegion_z2	1	-0.0522	0.0521	-1.00	0.3165

Table B Estimation Result of the SRUM for Switching Region Sets by DML and Vessel Size of Tuna Purse Seine Fleet in EPO

The MDC Procedure

Conditional Logit Estimates

DML=1 Vessel_Size=4_XLarge (1,250-1,800t)

Parameter Estimates

Parameter	DF	Estimate	Standard Error	t Value	Approx Pr > |t|
lNOA_LastRegion_z3	1	-0.0186	0.0576	-0.32	0.7465
lNOA_LastRegion_z4	1	-0.002846	0.0463	-0.06	0.9509
lNOA_LastRegion_z5	1	0.008389	0.0488	0.17	0.8635
lNOA_LastRegion_z6	1	0.0556	0.0542	1.02	0.3055
lNOA_LastRegion_z7	1	0.0432	0.0450	0.96	0.3372
lNOA_LastRegion_z8	1	0.0739	0.0452	1.63	0.1022
lNOA_LastRegion_z9	1	0.0840	0.0476	1.77	0.0775
lNOA_LastRegion_z10	1	0.0767	0.0472	1.63	0.1040
lNOA_LastRegion_z11	1	0.0966	0.0511	1.89	0.0587
lYFT_LastRegion_z1	1	-0.0314	0.0557	-0.56	0.5726
lYFT_LastRegion_z2	1	-0.0559	0.0403	-1.39	0.1659
lYFT_LastRegion_z3	1	-0.1544	0.0640	-2.41	0.0158
lYFT_LastRegion_z4	1	-0.0843	0.0378	-2.23	0.0257
lYFT_LastRegion_z5	1	-0.0757	0.0393	-1.93	0.0542
lYFT_LastRegion_z6	1	-0.1188	0.0600	-1.98	0.0478
lYFT_LastRegion_z7	1	-0.1050	0.0377	-2.79	0.0053
lYFT_LastRegion_z8	1	-0.0967	0.0375	-2.58	0.0098
lYFT_LastRegion_z9	1	-0.1023	0.0422	-2.43	0.0153
lYFT_LastRegion_z10	1	-0.0517	0.0403	-1.28	0.2002
lYFT_LastRegion_z11	1	-0.0907	0.0439	-2.07	0.0385
lBET_LastRegion_z1	1	-0.0329	0.0597	-0.55	0.5817
lBET_LastRegion_z2	1	0.0146	0.0379	0.39	0.6995
lBET_LastRegion_z3	1	-0.0888	0.0777	-1.14	0.2528
lBET_LastRegion_z4	1	0.0243	0.0359	0.68	0.4985
lBET_LastRegion_z5	1	0.0420	0.0382	1.10	0.2720
lBET_LastRegion_z6	1	-0.0449	0.1003	-0.45	0.6541
lBET_LastRegion_z7	1	0.0195	0.0362	0.54	0.5898
lBET_LastRegion_z8	1	0.0245	0.0354	0.69	0.4884
lBET_LastRegion_z9	1	-0.006969	0.0449	-0.16	0.8768

Table B Estimation Result of the SRUM for Switching Region Sets by DML and Vessel Size of Tuna Purse Seine Fleet in EPO

The MDC Procedure

Conditional Logit Estimates

DML=1 Vessel_Size=4_XLarge (1,250-1,800t)

Parameter Estimates

Parameter	DF	Estimate	Standard Error	t Value	Approx Pr > |t|
lBET_LastRegion_z10	1	-0.0487	0.0402	-1.21	0.2260
lBET_LastRegion_z11	1	0.0250	0.0472	0.53	0.5961
lSKJ_LastRegion_z1	1	0.0448	0.0479	0.94	0.3495
lSKJ_LastRegion_z2	1	0.0514	0.0437	1.18	0.2393
lSKJ_LastRegion_z3	1	0.0741	0.0474	1.56	0.1178
lSKJ_LastRegion_z4	1	0.0355	0.0411	0.86	0.3880
lSKJ_LastRegion_z5	1	0.009603	0.0434	0.22	0.8249
lSKJ_LastRegion_z6	1	0.0449	0.0490	0.92	0.3589
lSKJ_LastRegion_z7	1	0.0513	0.0404	1.27	0.2041
lSKJ_LastRegion_z8	1	0.0114	0.0409	0.28	0.7813
lSKJ_LastRegion_z9	1	0.0195	0.0435	0.45	0.6541
lSKJ_LastRegion_z10	1	0.0555	0.0433	1.28	0.1993
lSKJ_LastRegion_z11	1	0.0125	0.0466	0.27	0.7887
SST_DOL_L	1	-0.001017	0.000656	-1.55	0.1209
SST_DOL_H	1	-0.001134	0.001464	-0.77	0.4385
O2_DOL_L	1	-0.0667	0.0248	-2.69	0.0072
O2_DOL_H	1	-0.0201	0.004202	-4.78	<.0001
SSH_DOL_L	1	-0.004330	0.001333	-3.25	0.0012
SSH_DOL_H	1	0.002667	0.000870	3.07	0.0022
MLD_DOL_L	1	0.002023	0.001973	1.03	0.3052
MLD_DOL_H	1	-0.002223	0.000567	-3.92	<.0001
CHLORO_DOL_L	1	0.001268	0.000850	1.49	0.1358
CHLORO_DOL_H	1	-0.000741	0.002178	-0.34	0.7337
MEI_z1	1	-0.2608	0.1967	-1.33	0.1849
MEI_z2	1	-0.6579	0.1632	-4.03	<.0001
MEI_z3	1	-0.2559	0.1864	-1.37	0.1698
MEI_z4	1	-0.5986	0.1315	-4.55	<.0001
MEI_z5	1	-0.1785	0.1302	-1.37	0.1705
MEI_z6	1	-0.3137	0.1808	-1.74	0.0827

Table B Estimation Result of the SRUM for Switching Region Sets by DML and Vessel Size of Tuna Purse Seine Fleet in EPO

The MDC Procedure

Conditional Logit Estimates

DML=1 Vessel_Size=4_XLarge (1,250-1,800t)

Parameter Estimates

Parameter	DF	Estimate	Standard Error	t Value	Approx Pr > |t|
MEI_z7	1	-0.4099	0.1275	-3.21	0.0013
MEI_z8	1	-0.3050	0.1298	-2.35	0.0188
MEI_z9	1	-0.4105	0.1471	-2.79	0.0053
MEI_z10	1	-0.2523	0.1391	-1.81	0.0696
MEI_z11	1	-0.1370	0.1568	-0.87	0.3824
