# Supplementary material for: Modeling the Spatial Dynamics of International Tuna Fleets
Source: PLoS One. 2016 Aug 18;11(8):e0159626. doi: 10.1371/journal.pone.0159626 (PMC4990267; doi:10.1371/journal.pone.0159626)
Supplement: S3 Appendix — Fig A. Observed and predicted choices of first set location model for each region. (PDF) [file pone.0159626.s003.pdf]

Period=1997-2011 DML=0 Vessel_Size=1_Small (363-700t)

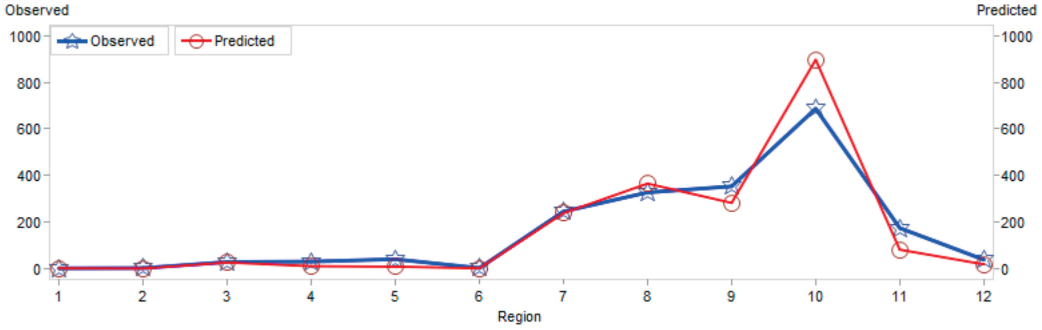

Period=1997-2011 DML=0 Vessel_Size=2_Median (700-1,050t)

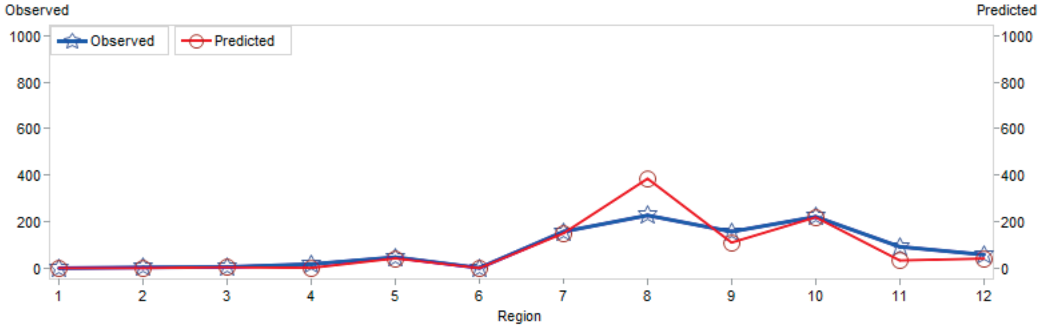

Period=1997-2011 DML=0 Vessel_Size=3_Large (1,050-1,250t)

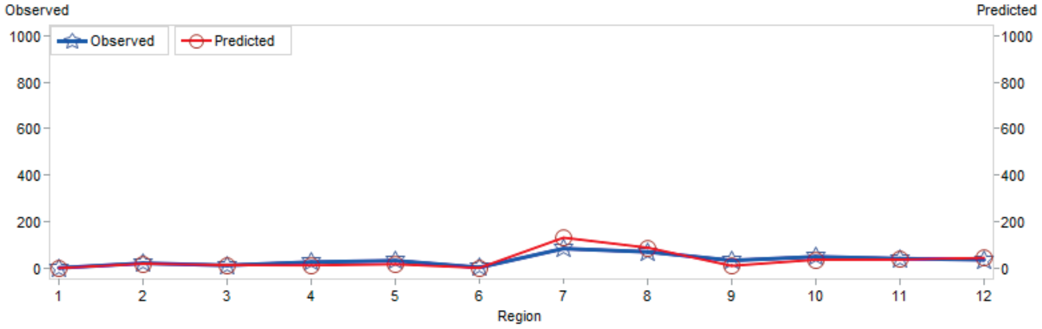

Period=1997-2011 DML=0 Vessel_Size=4_XLarge (1,250-1,800t)

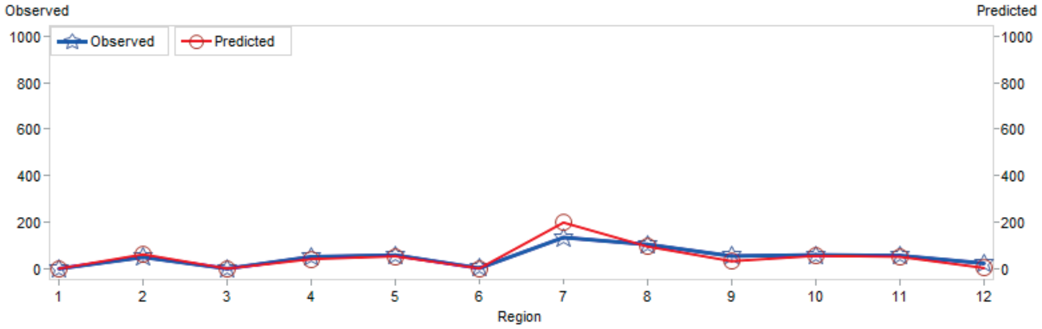

Appendix Figure A. Observed and predicted choices of first set location choice model for each region.

Period=1997-2011 DML=1 Vessel_Size=1_Small (363-700t)

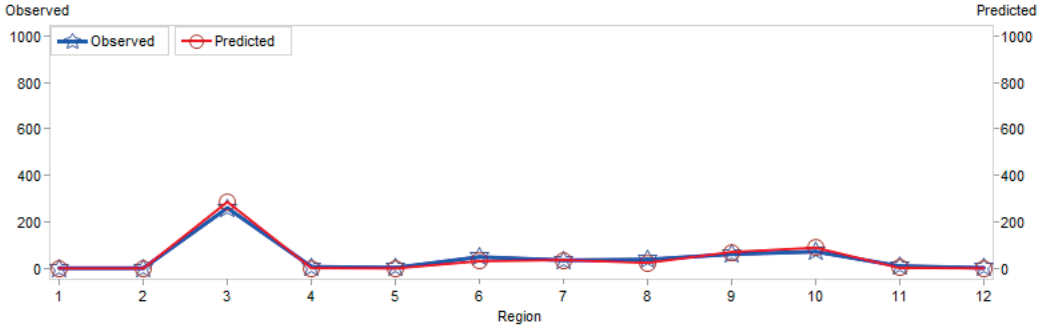

Period=1997-2011 DML=1 Vessel_Size=2_Median (700-1,050t)

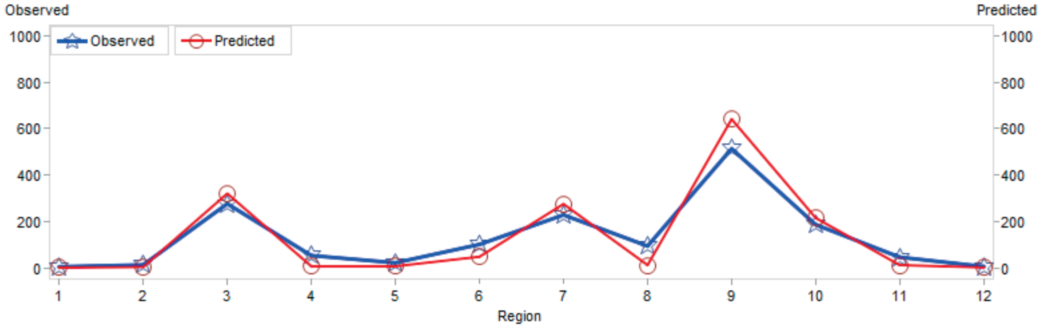

Period=1997-2011 DML=1 Vessel_Size=3_Large (1,050-1,250t)

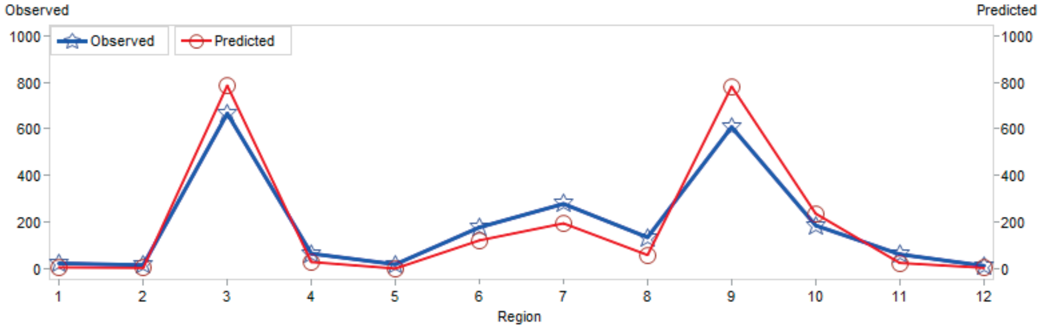

Period=1997-2011 DML=1 Vessel_Size=4_XLarge (1,250-1,800t)

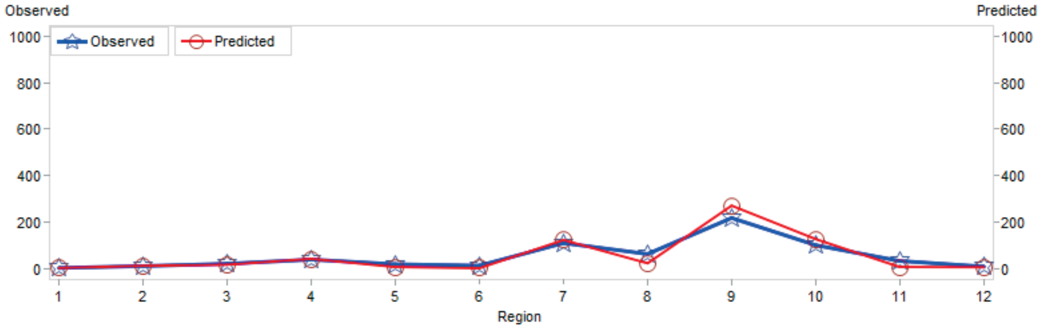

Appendix Figure A. Observed and predicted choices of first set location choice model for each region.

Period=2012 DML=0 Vessel_Size=1_Small (363-700t)

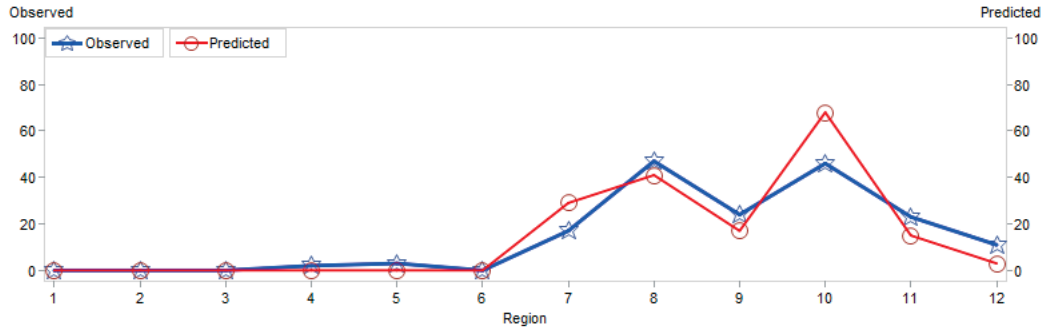

Period=2012 DML=0 Vessel_Size=2_Median (700-1,050t)

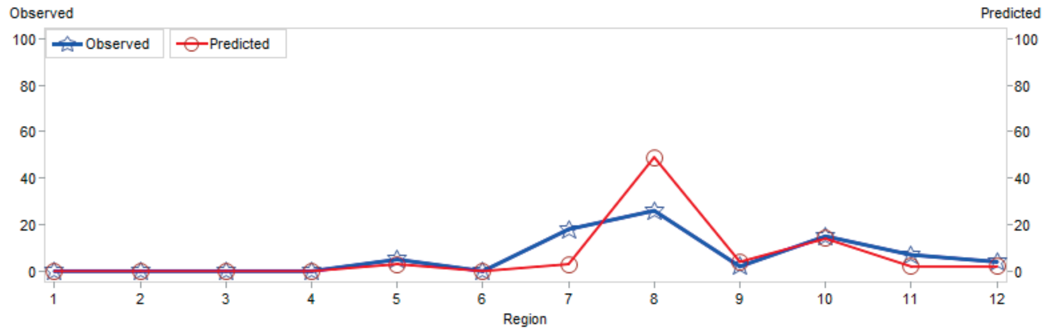

Period=2012 DML=0 Vessel_Size=3_Large (1,050-1,250t)

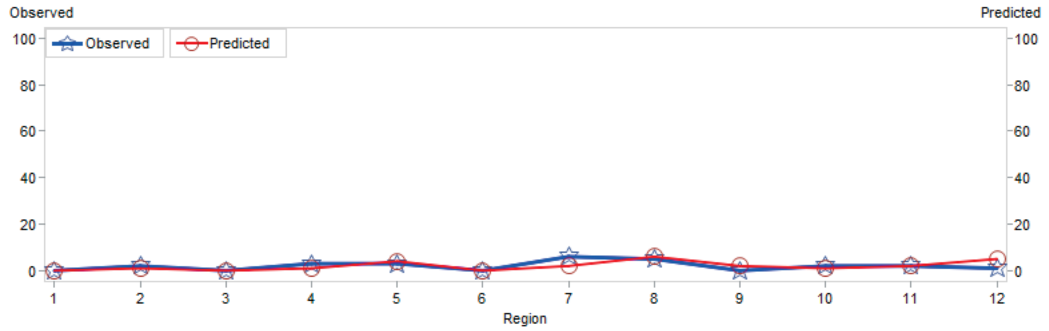

Period=2012 DML=0 Vessel_Size=4_XLarge (1,250-1,800t)

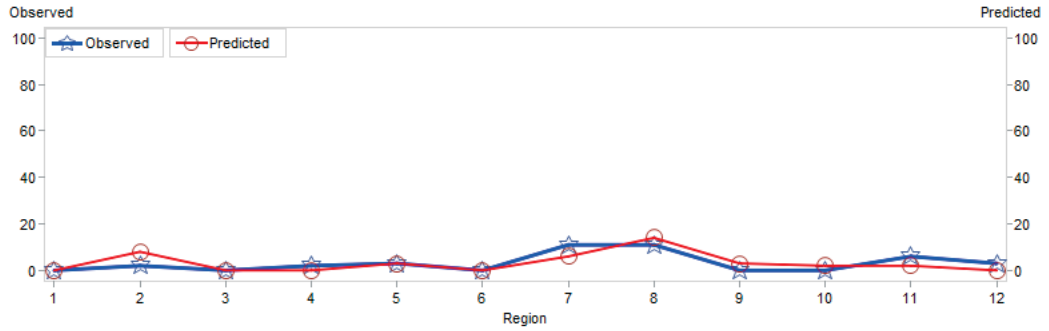

Appendix Figure A. Observed and predicted choices of first set location choice model for each region.

Period=2012 DML=1 Vessel_Size=1_Small (363-700t)

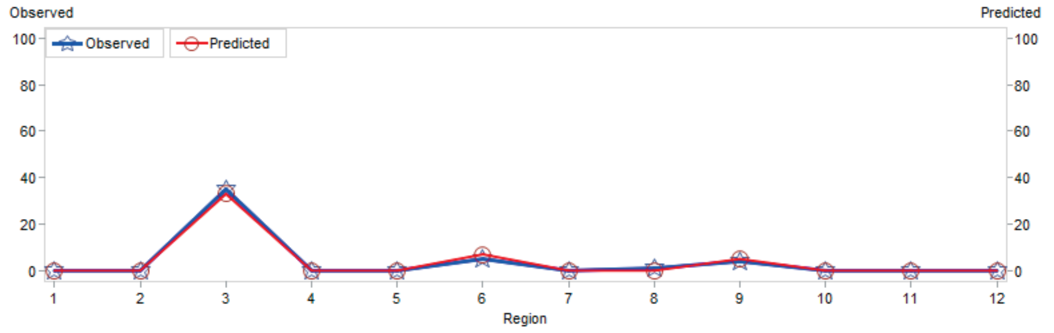

Period=2012 DML=1 Vessel_Size=2_Median (700-1,050t)

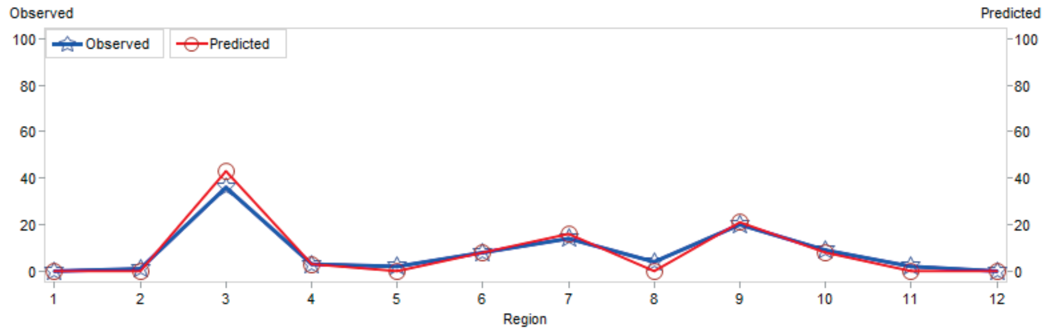

Period=2012 DML=1 Vessel_Size=3_Large (1,050-1,250t)

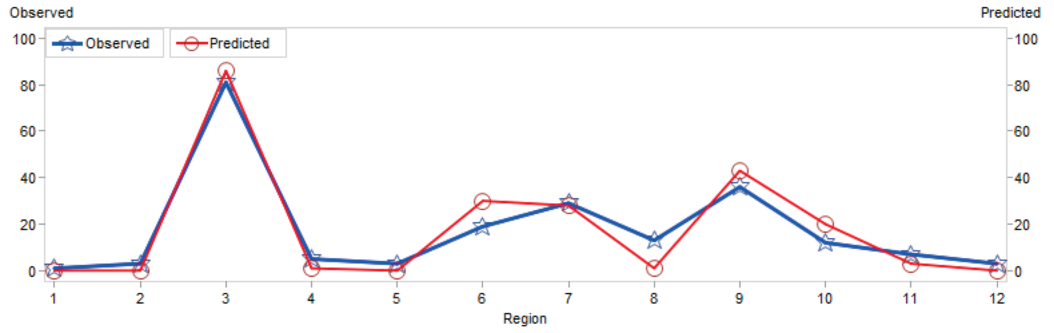

Period=2012 DML=1 Vessel_Size=4_XLarge (1,250-1,800t)

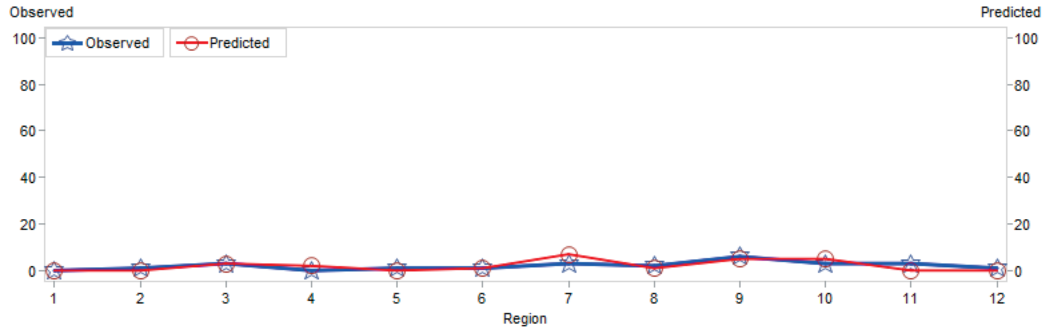

Appendix Figure A. Observed and predicted choices of first set location choice model for each region.
